# Lumenal Galectin-9-Lamp2 interaction regulates lysosome and autophagy to prevent pathogenesis in the intestine and pancreas

**DOI:** 10.1038/s41467-020-18102-7

**Published:** 2020-08-27

**Authors:** Janaki N. Sudhakar, Hsueh-Han Lu, Hung-Yu Chiang, Ching-Shu Suen, Ming-Jing Hwang, Sung-Yu Wu, Chia-Ning Shen, Yao-Ming Chang, Fu-An Li, Fu-Tong Liu, Jr-Wen Shui

**Affiliations:** 1 Institute of Biomedical Sciences, Academia Sinica, Taipei, Taiwan; 2 Genomics Research Center, Academia Sinica, Taipei, Taiwan

**Keywords:** Cell biology, Immunology, Diseases, Gastroenterology, Pathogenesis

## Abstract

Intracellular galectins are carbohydrate-binding proteins capable of sensing and repairing damaged lysosomes. As in the physiological conditions glycosylated moieties are mostly in the lysosomal lumen but not cytosol, it is unclear whether galectins reside in lysosomes, bind to glycosylated proteins, and regulate lysosome functions. Here, we show in gut epithelial cells, galectin-9 is enriched in lysosomes and predominantly binds to lysosome-associated membrane protein 2 (Lamp2) in a Asn(N)-glycan dependent manner. At the steady state, galectin-9 binding to glycosylated Asn^175^ of Lamp2 is essential for functionality of lysosomes and autophagy. Loss of N-glycan-binding capability of galectin-9 causes its complete depletion from lysosomes and defective autophagy, leading to increased endoplasmic reticulum (ER) stress preferentially in autophagy-active Paneth cells and acinar cells. Unresolved ER stress consequently causes cell degeneration or apoptosis that associates with colitis and pancreatic disorders in mice. Therefore, lysosomal galectins maintain homeostatic function of lysosomes to prevent organ pathogenesis.

## Abstract (web summary)

Galectins are carbohydrate binding proteins previously implicated in sensing and repairing damaged lysosomes. Here, the authors show that galectin-9 has specific lysosomal roles in autophagy and contributes to cell degeneration and apoptosis in colitis and pancreatitis in mice.

## Introduction

Susceptibility to lysosomal membrane permeabilization (LMP) is associated with lysosome dysfunction, cell death, and disease pathogenesis^1,2^. One consequence of LMP is the leakage of lysosomal hydrolases into the cytosol, posing a risk for lysosomal cell death^2^. Importantly, lysosomal damage or dysfunction has been linked to neurodegeneration or inflammatory bowel disease (IBD) in humans and targeting lysosomes for LMP-induced cell death is considered as a potential strategy for cancer therapy^3–5^. Therefore, maintenance of lysosomal membrane integrity not only protects cells physically but also facilitates lysosome functionality to prevent intracellular stress and death^4^. The heavily glycosylated Lamp proteins were modeled and shown to constitute an ~8 nm-thick glycoprotein coat called glycocalyx, which associates with the lysosomal membrane and helps build or maintain its physical integrity^6^. Most Asn(N)-linked glycosylations are high-molecular-weight complex poly-N-acetyl-lactosamine (LacNAc) moieties which can protect the membrane from degradation by lysosomal hydrolases^7^. Removal of N-linked glycans from Lamp1/2 causes their rapid proteolysis^8^. As such, lysosome-associated membrane protein 2 (Lamp2) depletion in cells causes increased susceptibility to cell death and impaired lysosome fusion, and is involved in human pancreatitis^9,10^. In contrast, overexpression of Lamp2 can reverse stress-induced autophagic flux blockade and subsequently alleviate LMP-induced lysosomal cell death^11^. Loss of Lamp2 in humans leads to Danon disease, a lysosome and autophagy disorder with cardiomyopathy, skeletal myopathy, and mental retardation^12^. While glycosylation constitutes about 60% of the total protein mass of Lamp, little is known about whether or how glycosylation, beyond simply adding carbohydrates to the core protein, contributes to lysosomal membrane integrity, lysosome function, or pathogenesis, especially under physiological conditions.

Intracellular functions for galectins have been documented in the context of their nuclear or cytosolic ligands^13^. Due to absence of complex glycans in the cytosol, cytosolic galectins were shown, likely only after vesicle rupture, to mediate a host surveillance mechanism for detection, repair, and clearance of damaged vesicles including lysosomes^4^. Such lysosome-mediated lysophagy is believed to be aided by cytosolic galectins^4,14^, including galectin-3 (Gal-3), Gal-8, and Gal-9, which can sense sterile lysosomal damage and are rapidly recruited to damaged lysosomes via binding to exposed glycans from the lysosomal lumen^15^. At the molecular level, Gal-3 was shown to cooperate with TRIM16 and ATG16L1 to direct autophagy^16^, while Gal-8 can recruit NDP52 to initiate anti-bacterial autophagy to defend cells against infection^15^. In addition to this type of intracellular function, endogenous galectins can be secreted and endocytosed to reside in the endocytic or recycling compartments. For example, internalized Gal-3 and Gal-4 are involved in the apical endocytic-recycling of glycoproteins^17,18^. Similarly, endocytosed Gal-9 has been shown to accumulate in lysosomes and is involved in apical sorting of proteins and lipids, as well as in the regulation of cancer cell death via autophagy or ubiquitination^19–21^. Intriguingly, while galectins at the steady state via endocytosis have access to endolysosomal compartments which contain abundant lumenal glycosylated membrane proteins, it has not been explored that under the physiological conditions whether intra-organelle or endocytic galectins could physically bind to glycosylated vesicular membrane proteins or whether such lumenal interaction affects structure, conformation, maturation or functionality of endocytic compartments. In this study, we reveal that in gut epithelial cells lysosomal Gal-9 preferentially binds to N-glycosylated Lamp2 at the steady state to stabilize lysosomes, maintain lysosomal acidity, and facilitate lysosome-mediated autophagy. More importantly, we show that loss of Gal-9 in mice causes unstable lysosomes in autophagy-active intestinal Paneth cells and pancreatic acinar cells at the steady state, which consequently compromises autophagy functionality leading to increased susceptibility to disease pathogenesis.

## Results

### Gal-9-mediated lysosome function regulates Paneth cells

**Fig. 1:**
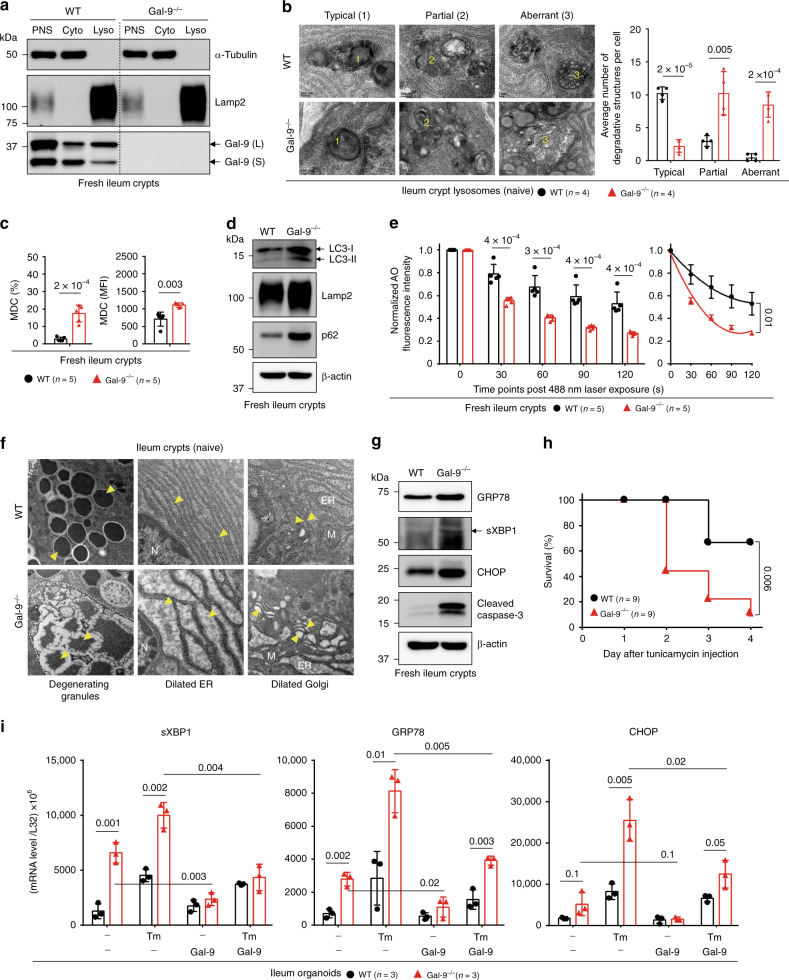
Gal-9-mediated lysosome function alleviates Paneth cell ER stress. **a** Western blot analysis of subcellular fractionation of fresh ileum crypts. PNS: post nuclear supernatant, Cyto: cytosol fraction, Lyso: lysosome fraction. **b** Transmission electron microscopy (TEM) analysis of ileum Paneth cells. Lysosomes with typical, partial, or aberrant morphology are indicated and quantified. **c** Flow cytometry analysis of ileum crypts isolated from naïve mice and stained with monodansylcadaverine (MDC) to detect autophagic vacuoles. **d** Western blot analysis of autophagy markers in fresh ileum crypts. **e** Freshly isolated crypts were labeled with acridine orange (AO) and then exposed to a 488 nm laser to induce lysosomal damage. Confocal images after laser exposure were taken at the indicated time points. Loss of lysosome stability is determined by the decrease of AO red fluorescence over time which is normalized to cells exposed at 0 s. **f** TEM analysis of Paneth cells with normal (in wild-type) or degenerating (in Gal-9^−/−^) electron-dense core secretory vesicles, indicated by arrowheads (left panels). Normal well-developed granular ER with intact lacey strands (in wild-type) or abnormally dilated ER (in Gal-9^−/−^) around the nucleus of Paneth cells is indicated (middle panels). Normal (in wild-type) or abnormally dilated Golgi complex (in Gal-9^−/−^) around the ER region of Paneth cells is indicated (right panels). N: nucleus, ER: endoplasmic reticulum, M: mitochondria. **g** Western blot analysis of ER stress and apoptosis markers in ileum crypts. **h** Survival curve of tunicamycin-injected mice. **i** Quantitative real-time PCR analysis of ER stress-associated genes in ileum organoids which were cultured with or without recombinant mouse Gal-9, stimulated with or without tunicamycin to induce ER stress. Each symbol represents organoids derived from one mouse. Data shown are representative or combined (**h**) results from two independent reproducible experiments. Statistical significance (*p* value) is indicated (**b**, **c**, **e**, **i**: Unpaired two-tailed *t*-test, **h**: Kaplan–Meier survival curves). Data are presented as mean ± SD. Source data are provided as a Source data file.

Lysosome dysfunction, unresolved endoplasmic reticulum (ER) stress, and impaired autophagy are pronounced factors for intestinal pathogenesis especially in autophagy-active Paneth cells^5,22,23^. As Gal-9 was previously identified as an autophagy-sensitive factor that affects anti-mycobacterial response and therefore reported as a risk factor for Crohn’s disease (CD)^24,25^, we investigated the expression of Gal-9 in the intestine. Quantitative real-time PCR and immunofluorescence analyses showed Gal-9 is predominantly expressed in gut epithelial cells located at the crypts. Specifically, Gal-9 is weakly expressed in Lgr5^+^ epithelial stem cells but highly enriched in MMP7^+^ Paneth cells (Supplementary Fig. 1a–c). Similar to mouse tissues, Gal-9 is also abundantly expressed in colon crypts in healthy human tissues but significantly lower in patients with colorectal cancer (CRC), inflammation (IBD), ulcerative colitis (UC), and CD (Supplementary Fig. 1d, e), especially in CD patients which might suggest why Gal-9 is a risk factor^25^. Furthermore, in the same CRC patient, Gal-9 expression was frequently lower in the tumor region, compared to non-tumor region (Supplementary Fig. 1f), suggesting Gal-9 could be involved in cancer development^20,26^. Intracellularly, we found in fresh crypts, Gal-9, in intestine-specific long form and common short form, is highly enriched in endolysosomes/lysosomes (Fig. 1a), which are known to contain extensively glycosylated lysosomal membrane proteins such as Lamp1/2, Limp1/2, and NPC1^7,27^. Under transmission electron microscope (TEM), loss of Gal-9 in mice led to increased accumulation of aberrant degradative lysosomes (Fig. 1b), or monodansylcadaverine (MDC)-positive autophagic vacuoles (Fig. 1c)^28^, indicative of autophagy blockade^5^. Indeed, fresh Gal-9^−/−^ crypts showed increased accumulation of LC3, Lamp2, and p62, the hallmark of impaired autophagy (Fig. 1d). As defective autophagy contributes to LMP leading to lysosome dysfunction or cell death^11,29^, we analyzed LMP in acridine orange (AO)-labeled crypts by real-time imaging of lysosomal membrane integrity, which was assayed by measuring the reduction of AO intensity (shown as red puncta in acidic lysosomes) over time post photo-oxidation^30^. Notably, Gal-9^−/−^, but not Gal-1^−/−^ or Gal-3^−/−^ crypts, showed increased LMP after challenge (Fig. 1e, Supplementary Fig. 2a), indicating Gal-9 protected lysosomes against intracellular stress. As autophagy, which can be triggered by ER stress, serves as a protective mechanism to resolve ER stress^23,31^, we examined whether loss of Gal-9 is associated with increased ER stress. Indeed, examination of Gal-9^−/−^ crypts by TEM revealed massive granule degeneration and dilated ER and Golgi structures within Paneth cells (Fig. 1f), which are typical characteristics of autophagy impairment that associates with unresolved ER stress^23,32^. Correlated to this, Gal-9^−/−^ crypts, especially at the crypt base where Paneth cells are located, showed enhanced unfolded protein response (UPR)^33^, as evidenced by elevated basal levels of ER stress markers, including calreticulin, GRP78, sXBP1, and CHOP, as well as increased cell apoptosis (Fig. 1g, Supplementary Fig. 2b–d). As a result, Gal-9^−/−^ mice were more susceptible to tunicamycin (Tm)-induced ER stress in vivo (Fig. 1h, Supplementary Fig. 2d, e). We next established primary organoids from crypts to ask whether Gal-9-mediated ER stress is cell-intrinsic to epithelial cells. Notably, Gal-9^−/−^ organoids recapitulated those phenotypes observed in fresh crypts, showing higher basal or Tm-induced ER stress, compared to wild-type organoids (Fig. 1i). Furthermore, exogenous recombinant Gal-9 can effectively reduce basal or Tm-induced ER stress in Gal-9^−/−^ organoids. As activation of UPR and accumulation of ER stress might create a tumor-supporting microenvironment for cancer development^34^, our results therefore support why reduced Gal-9 expression in colon tissues might be associated with CRC development. Taken together, by promoting lysosome-mediated autophagy, Gal-9 targets autophagy-active Paneth cells in the intestine to maintain their homeostasis and help resolve ER stress.

### Gal-9 regulates autophagy in Paneth cells to prevent colitis

**Fig. 2:**
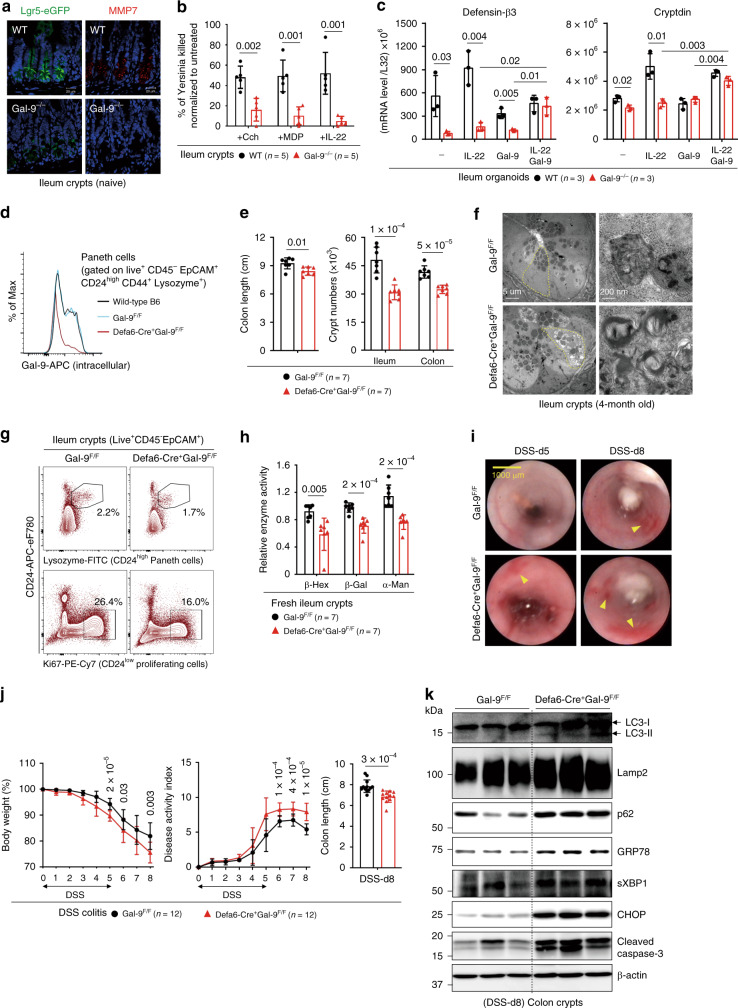
Gal-9 regulates lysosomes in Paneth cells to prevent inflammation. **a** Immunofluorescence analysis of stem-cell marker Lgr5 (with Lgr5-eGFP reporter mice) (left panels) and Paneth cell marker MMP7 (right panels) in ileum crypts. **b** Freshly isolated ileum crypts were stimulated with carbachol (Cch), muramyl dipeptide (MDP), or IL-22 to induce release of anti-microbial contents. The supernatants were then incubated with live *Yersinia* and the percentage of bacterial killing was calculated after normalized to unstimulated crypts. **c** Quantitative real-time PCR analysis of anti-microbial peptides in ileum organoids which were cultured with recombinant mouse Gal-9, stimulated with IL-22, or both. Each symbol represents organoids derived from one mouse. **d** Flow cytometry analysis of intracellular Gal-9 levels in the gated Paneth cells in ileum crypts. **e** Colon length was measured and freshly isolated crypts from naïve mice were counted under phase-contrast microscopy and quantified. **f** Electron microscopy images of ileum crypts with Paneth cells outlined in yellow (left panels). Vacuoles containing concentric multi-lamellar (fingerprint-like) membrane structures, indicative of impaired autophagy, were observed in Defa6-Cre^+^Gal-9^F/F^ mice (lower right panel). **g** Flow cytometry analysis of CD24^high^ Lysozyme^+^ Paneth cells and CD24^low^ Ki67^+^ proliferating cells in ileum crypts from naïve mice. **h** Lysosomal hydrolase activity of freshly isolated ileum crypts was determined by specific substrates. **i** DSS-treated mice at day-5 or day-8 were analyzed for colon internal bleeding (indicated by yellow arrowheads) by endoscopy. **j** Percentage of body weight, disease activity index (combined scores of weight loss, rectal bleeding, and stool consistency), and colon length in DSS-treated mice were measured. **k** Western blot analysis of autophagy, ER stress, and apoptosis markers in fresh colon crypts isolated from DSS-treated mice at day-8. Data shown are representative results from two independent reproducible experiments. Statistical significance (*p* value) is indicated (**b**, **c**, **e**, **h**, **j**: Unpaired two-tailed t-test). Data are presented as mean ± SD. Source data are provided as a Source data file.

As Gal-9 is predominantly expressed at the crypt base, we asked whether Gal-9 controls crypt homeostasis, which is mainly governed by the number of epithelial stem cells and Paneth cells located at this position^35^. Notably, under the physiological conditions, total crypt numbers in the ileum and colon were significantly reduced in Gal-9^−/−^ mice (Supplementary Fig. 2f), suggesting Gal-9 regulates epithelial homeostasis in vivo. As unresolved ER stress might cause loss of epithelial stem cells or Paneth cells leading to enteritis^22,36^, it is possible increased ER stress in Gal-9^−/−^ crypts might compromise the survival and/or function of stem cells and Paneth cells. Indeed, there were fewer Lgr5^+^ epithelial stem cells or MMP7^+^ Paneth cells in Gal-9^−/−^ crypts (Fig. 2a). Functionally, the percentage of CD24^low^ Ki67^+^ proliferating transit-amplifying (TA) cells and in vivo Edu incorporation in Gal-9^−/−^ crypts were both significantly decreased (Supplementary Fig. 2g, h)^37^, indicative of reduced regeneration capacity. CD24^+^ Lysozyme-producing Paneth cells were also significantly reduced in Gal-9^−/−^ crypts (Supplementary Fig. 2i)^37^. Regarding to immunity, we found Paneth-cell defect results in reduced bacteria killing by fresh crypts, which was assayed by anti-microbial contents directly released from stimulated crypts (Fig. 2b)^38^. Using primary organoids, we confirmed loss of Gal-9 compromised the percentage of Lgr5^+^ stem cells and Lysozyme^+^ Paneth cells, while exogenous recombinant Gal-9 effectively rescued the defects of growth and differentiation (Supplementary Fig. 2j). When immunity was tested, we found Gal-9^−/−^ organoids showed less basal and IL-22-induced production of anti-microbial peptides (AMP), which could also be restored by exogenous recombinant Gal-9 (Fig. 2c). Intriguingly, while Gal-9^−/−^ crypts showed reduced regeneration capability, slightly increased apoptosis, and reduced anti-microbial immunity at the steady state, Gal-9^−/−^ mice surprisingly do not develop spontaneous colitis at least at the age of 8 months.

To gain more insights whether Gal-9 predominantly targets Paneth cells in vivo, we generated Paneth cell-specific (Defa6-Cre^+^Gal-9^flox/flox^) Gal-9 conditional knockout mice. Defa6-Cre mice drives Cre expression via the α-defensin promoter which is specific to Paneth cells^22^. We first analyzed and confirmed Gal-9 deletion in Paneth cells by gating on CD24^high^ Lysozyme-producing crypt cells (Fig. 2d)^37^. Reproducibly, conditional Gal-9 deletion caused colon injury, a decrease in total crypt numbers, and autophagy blockade that likely associate with Paneth cell degeneration (Fig. 2e, f)^23^. Functionally, while there were fewer CD24^high^ Lysozyme-producing Paneth cells (Fig. 2g, upper panels), CD24^low^ Ki67^+^ proliferating transit-amplifying or stem cells were also reduced when Gal-9 was conditionally ablated in Paneth cells (Fig. 2g, lower panels). The stem-cell defect was likely due to disrupted niche regulation between Paneth cells and stem cells^35,39^, where Gal-9^−/−^ Paneth cells might not produce sufficient niche factors to support nearby stem cells. Notably, fresh crypts also showed reduced lysosomal hydrolase activity (Fig. 2h), indicative of lysosome dysfunction in Gal-9^−/−^ Paneth cells. Similar to global knockout mice, Paneth cell-specific Gal-9 conditional knockout mice were more susceptible to dextran sulfate sodium (DSS)-induced colitis, showing increased colon internal bleeding, more body weight loss, higher disease activity index, and enhanced colon injury (Fig. 2i, j). Furthermore, there was increased accumulation of LC3, Lamp2 and p62 in crypts (Fig. 2k), indicative of autophagy blockade in Gal-9^−/−^ Paneth cells. As secretory Paneth cells have high autophagic activity, as a result, they are prone to ER stress that could associate with increased apoptosis^22^. Indeed, analysis of crypts in DSS-treated mice showed specific loss of Gal-9 in Paneth cells not only caused autophagy block, but also increased ER stress and apoptosis (Fig. 2k). Together these genetic evidence indicate Gal-9-mediated autophagy in Paneth cells is needed to protect mice against colitis.

### Gal-9 regulates autophagy flux to prevent ER stress and LMP

**Fig. 3:**
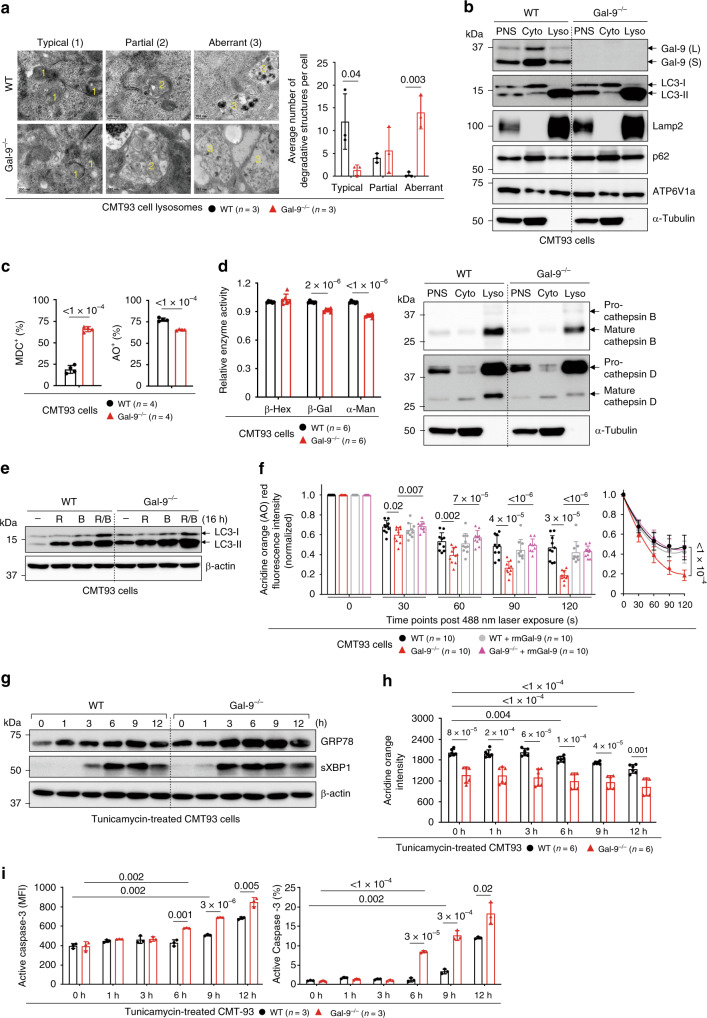
Gal-9 regulates lysosomes to protect cells from ER stress-induced apoptosis. **a** Electron microscopy analysis of lysosomes in CMT93 cells. Lysosomes with typical, partial or aberrant morphology were indicated and quantified. **b** Western blot analysis of subcellular fractionations of CMT93 cells. PNS: post nuclear supernatant, Cyto: cytosol fraction, Lyso: lysosome fraction. **c** Flow cytometry analysis of CMT93 cells, stained with monodansylcadaverine (MDC) to detect autophagic vacuoles or acridine orange (AO) to detect acidic compartments. **d** Lysosomal hydrolase activity of CMT93 cells was determined by specific substrates (left panel) or protein levels by Western blot analysis of subcellular fractionations (right panel). **e** Western blot analysis of autophagy flux by the LC3 marker in CMT93 cells, treated with rapamycin (R), bafilomycin (B), or both (R/B) for 16 h. **f** The indicated AO-labeled CMT93 cells were exposed to a 488 nm laser to induce lysosomal damage. Confocal images after laser exposure were taken at the indicated time points. Loss of lysosome stability was determined by the decrease of AO red fluorescence over time which was normalized to cells exposed at 0 s. **g** Western blot and time-course analysis of ER stress markers in tunicamycin-treated CMT93 cells. **h** Flow cytometry and time-course analysis of LMP response in tunicamycin-treated CMT93 cells. **i** Flow cytometry and time-course analysis of active caspase-3 in tunicamycin-treated CMT93 cells. Data shown are representative or combined (**d**, **h**) results from two independent reproducible experiments. Statistical significance (*p* value) is indicated (**a**, **c**, **d**, **f**, **h**, **i**: Unpaired two-tailed *t*-test). Data are presented as mean ± SD. Source data are provided as a Source data file.

To gain more insights about Gal-9-mediated autophagy, we abolished two forms of Gal-9 in colon epithelial cell CMT93 by CRISPR/Cas9 (Supplementary Fig. 3a). Similar to crypts, Gal-9^−/−^ CMT93 cells showed more aberrant lysosomes with partially digested materials^5^, increased accumulation of LC3 and Lamp2, more MDC^+^ autophagic vacuoles, and higher lysosomal pH that was associated with reduced lysosomal hydrolase activity (Fig. 3a–d). Furthermore, not only there was increased accumulation of Lamp2^+^ lysosomes in Gal-9^−/−^ cells, but those lysosomes were mostly peripherally localized in a manner reminiscent of Lamp1/2^−/−^ cells (Supplementary Fig. 3b, lower left panels)^40^. This indicates that Gal-9 might regulate autophagosome maturation or lysosome perinuclear mobilization needed for fusion^41^. To test this hypothesis, we exploited a lysosome rupture model induced by L-Leucyl-L-leucine methyl ester (LLOMe), a lysosomotropic compound known to induce lysosomal damage and facilitate perinuclear autophagosome formation to sequester damaged lysosomes^42,43^. By time-course (0–6 h) chasing of maturation and perinuclear formation or mobilization of Rab7^+^ CD63^+^ late endosomes or Rab7^+^ Lamp2^+^ endolysosomes post LLOMe (Supplementary Fig. 3c, both shown in large yellow perinuclear puncta)^28,43^, we confirmed that loss of Gal-9 indeed cause impaired perinuclear positioning of Lamp2^+^ Cathepsin-D^+^ lysosomes or Lamp2^+^ LC3^+^ autophagosomes during lysosomal damage (Supplementary Fig. 3b, d). The absence of perinuclear puncta was specifically caused by Gal-9 deficiency, as addition of exogenous recombinant Gal-9, but not recombinant Gal-3 or presence of other endogenous galectins, effectively restored puncta formation (Supplementary Fig. 3b, d, e). Reproducibly, only exogenous recombinant human Gal-9 can reduce accumulation of MDC^+^ autophagic vacuoles in human HT-29 Gal-9 knockdown (HT-29^KD^) epithelial cells (Supplementary Fig. 3f).

Next, we asked whether autophagy blockade in the absence of Gal-9 contributes to autophagy flux, which measures autophagic degradation and can be assayed by treating cells with autophagy-inducing rapamycin, with or without fusion-blocking bafiomycin^44^. Notably, there was more basal and rapamycin-induced accumulation of LC3-II in Gal-9^−/−^ cells, addition of bafilomycin further blocked LC3-II degradation (Fig. 3e), suggesting impaired autophagy flux which was also supported by increased p62 accumulation (Fig. 3b). To further confirm this, we analyzed endocytosis of exogenous red fluorochrome-labeled EGF and monitored perinuclear maturation and mobilization of green dye-labeled endosomes or blue dye-labeled lysosomes at the steady state by live-cell imaging during autophagic degradation^45^. Perinuclear localization of endolysosomes with endocytosed EGF (shown in white puncta) was clearly observed in wild-type cells 1 hr after EGF addition, but was absent in Gal-9^−/−^ CMT93 cells (Supplementary Fig. 3g), indicating loss of Gal-9 caused impaired lysosome perinuclear motility prior to fusion-degradation. Notably, addition of recombinant Gal-9 in Gal-9^−/−^ cells effectively restored defective cargo degradation (Supplementary Fig. 3g, lower right panels). Next, similar to crypts, impaired autophagy in Gal-9^−/−^ CMT93 cells also resulted in increased LMP response after photo-oxidation challenge (Fig. 3f)^30^, which can be restored by exogenous recombinant Gal-9. We next asked whether autophagy blockade in Gal-9^−/−^ CMT93 cells would cause more accumulated or unresolved ER stress leading to increased LMP and apoptosis^46,47^. By time-course (0–12 h) chasing of treated cells, we found tunicamycin-induced ER stress as early as 3 h, detectable LMP after 6 h, and apoptosis around 9 h in wild-type cells (Fig. 3g–i)^46^. However, in all cases, impaired autophagy due to loss of Gal-9 rendered cells more susceptible to ER stress at earlier time points, consequently resulting in increased LMP and apoptosis. Together, we conclude that Gal-9-mediated lysosome function promotes autophagy flux which reduces susceptibility of cells to ER stress, LMP and apoptosis.

### Gal-9 binds to Asn^175^ of Lamp2 to maintain lysosome function

**Fig. 4:**
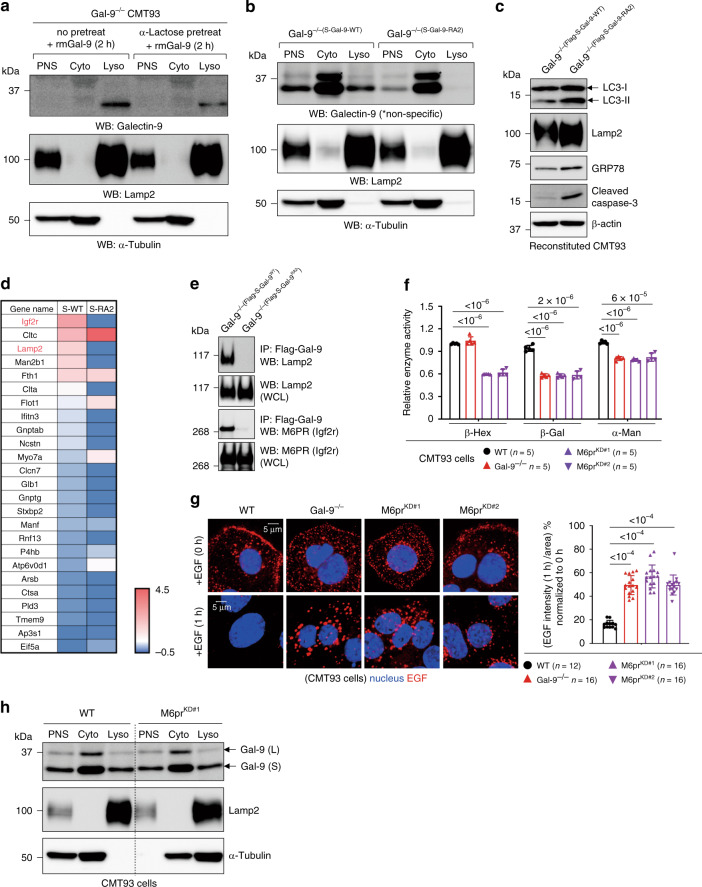
Gal-9 predominantly binds to endolysosomal/lysosomal M6PR and Lamp2. **a** Western blot analysis of subcellular fractionations for endocytosis and localization of exogenous recombinant mouse Gal-9 (rmGal-9) in rmGal-9-treated (for 2 h) Gal-9^−/−^ CMT93 cells, with or without prior α-lactose pre-treatment for 2 h. PNS: post nuclear supernatant, Cyto: cytosol fraction, Lyso: lysosome fraction. **b** Western blot analysis of subcellular fractionations for endogenously expressed Gal-9. Gal-9^−/−(S-Gal-9-WT)^ indicates Gal-9^−/−^ CMT93 cells stably reconstituted with S-form of wild-type Gal-9, while Gal-9^−/−(S-Gal-9-RA2)^ indicates Gal-9^−/−^ CMT93 cells reconstituted with S-form of glycan-binding-deficient (Arg^64/238^ to Ala^64/238^ or RA2) Gal-9. *non-specific. **c** Western blot analysis of autophagy, ER stress, and apoptosis markers in the indicated CMT93 cells. **d** Heatmap analysis showing the top 25 endolysosomal/lysosomal Gal-9-binding proteins in CMT93 cells, identified by mass spectrometry of wild-type and RA2-reconstituted Gal-9^−/−^ cells by pulling down Flag-tagged Gal-9. **e** Co-immunoprecipitation of Gal-9 and Lamp2 or M6PR in CMT93 cells, analyzed by Western blot analysis. **f** Lysosomal hydrolase activity of the indicated CMT93 cells was determined by specific substrates. **g** Immunofluorescence analysis of the indicated CMT93 cells for degradation of endocytosed fluorochrome-labeled EGF. Cells were pulsed with Alexa Flour 555-conjugated EGF for 5 min and confocal images were captured after 1 h post fixation (left panels). Percentage of EGF intensity/area was calculated and quantified at 1 h and normalized to 0 h by ZEN 2011 software (right panel). **h** Western blot analysis of subcellular fractionations for localization of Gal-9 in M6pr^KD^ CMT93 cells. PNS: post nuclear supernatant, Cyto: cytosol fraction, Lyso: lysosome fraction. Data shown are representative or combined (**f**) results from two independent reproducible experiments. Statistical significance (*p* value) is indicated (**f**, **g**: Unpaired two-tailed *t*-test). Data are presented as mean ± SD. Source data are provided as a Source data file.

As loss of Gal-9 affects various aspects of endolysosome/lysosome function at the steady state, we reason that those defects are unlikely mediated by cytosolic Gal-9 because under physiological conditions the cytosol is mostly devoid of complex glycans^15^. As galectins can be secreted and uptaken via endocytosis^13^, we tested in CMT93 cells and found that uptake of exogenous recombinant Gal-9 largely depends on its glycan-binding on the cell surface, as α-lactose pre-treatment of cells significantly abolished the uptake and endolysosomal/lysosomal localization of Gal-9 (Fig. 4a, Supplementary Fig. 4a)^20,48^. Intriguingly, 2 h after uptake, exogenous Gal-9 was exclusively localized in endolysosomes/lysosomes and almost undetectable in the cytosol. We next asked whether glycan-binding capability of Gal-9 is critical for its endolysosomal/lysosomal localization or regulatory function when Gal-9 is intrinsically expressed. To this end, Gal-9^−/−^ cells were stably reconstituted with either wild-type or glycan-binding-deficient mutants of Gal-9 (Gal-9^R64/269A^ and Gal-9^R64/238A^, or abbreviated as Gal-9-RA2, (Supplementary Fig. 4b, c)^49^. Indeed, we found glycan-binding capability of Gal-9 is required for its endolysosomal/lysosomal localization when Gal-9 is endogenously expressed (Fig. 4b). Loss of glycan-binding in Gal-9 also led to increased accumulation of LC3 and Lamp2, more MDC^+^ autophagic vacuoles, higher lysosomal pH, increased LMP response, elevated ER stress, and increased basal or stress-induced apoptosis (Fig. 4c, Supplementary Fig. 4d–g). These results indicate that Gal-9 binding to glycosylated proteins in the endolysosomal/lysosomal lumen is critical for its lysosomal localization and regulatory function. Next, to identify Gal-9-binding proteins in endolysosomes/lysosomes, mass spectrometry of wild-type or Gal-9-RA2-reconstituted Gal-9^−/−^ CMT93 cells at the steady state was performed by pull-down of Flag-tagged Gal-9. Notably, the volcano plot identified and showed differential Gal-9-binding proteins between glycan-binding sufficient (Gal-9-WT) and deficient (Gal-9-RA2) cells with adjusted p-value significance (Supplementary Fig. 4h). Next, the endolysosomal/lysosomal protein coding genes, based on a database, were selected from the LC-MS/MS result and a heatmap with a high cut-off threshold (score ≥ 300) was generated (Fig. 4d). The map shows the top 25 endolysosomal/lysosomal Gal-9-binding targets in wild-type versus glycan-binding deficient cells. Among the list, Igf2r (Insulin-like growth factor 2 receptor or cation-independent mannose-6-phosphate receptor, CI-M6PR) and Lamp2 were identified as the predominant Gal-9-binding proteins in endolysosomes/lysosomes. N-glycan-dependent Gal-9-M6PR and Gal-9-Lamp2 interactions were further confirmed by co-immunoprecipitation (Fig. 4e).

**Fig. 5:**
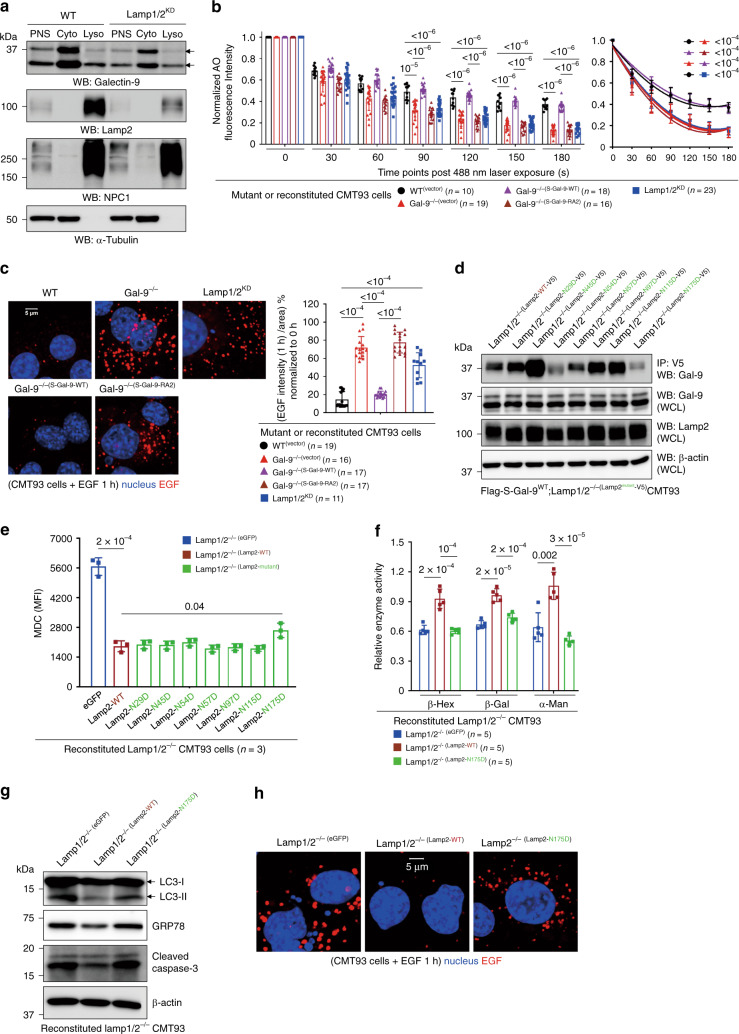
Gal-9 binding to glycosylated Asn^175^ in Lamp2 is critical for lysosome function. **a** Western blot analysis of subcellular fractionations for localization of endogenous Gal-9 in Lamp1/2^KD^ CMT93 cells. PNS: post nuclear supernatant, Cyto: cytosol fraction, Lyso: lysosome fraction. **b** The indicated acridine orange (AO)-labeled CMT93 cells were exposed to a 488-nm laser to induce lysosomal damage. Confocal images after laser exposure were taken at the indicated time points. Loss of lysosome stability was determined by the decrease of AO red fluorescence over time which was normalized to cells exposed at 0 s. **c** Immunofluorescence analysis of the indicated CMT93 for degradation of endocytosed fluorochrome-labeled EGF. Cells were pulsed with Alexa Flour 555-conjugated EGF for 5 min and confocal images were captured after 1 h post fixation (left panels). Percentage of EGF intensity/area was calculated and quantified at 1 h and normalized to 0 h (right panel). **d** Co-immunoprecipitation of Gal-9 and Lamp2 in Lamp1/2^−/−^ CMT93 cells, reconstituted with Flag-tagged Gal-9 and V5-tagged Lamp2 mutants. **e** Flow cytometry analysis of the indicated Lamp2 mutant-reconstituted Lamp1/2^−/−^ CMT93 cells, stained with monodansylcadaverine (MDC) to detect autophagic vacuoles. **f** Lysosomal hydrolase activity of the indicated CMT93 cells was determined by specific substrates. **g** Western blot analysis of autophagy, ER stress, and apoptosis markers in Lamp2-reconstituted CMT93 cells. **h** Immunofluorescence analysis of Lamp2-reconstituted CMT93 cells for degradation of endocytosed fluorochrome-labeled EGF by the method shown in (**c**). Data shown are representative results from two independent reproducible experiments. Statistical significance (*p* value) is indicated (**b**, **c**, **e**, **f**: Unpaired two-tailed *t*-test). Data are presented as mean ± SD. Source data are provided as a Source data file.

**Fig. 6:**
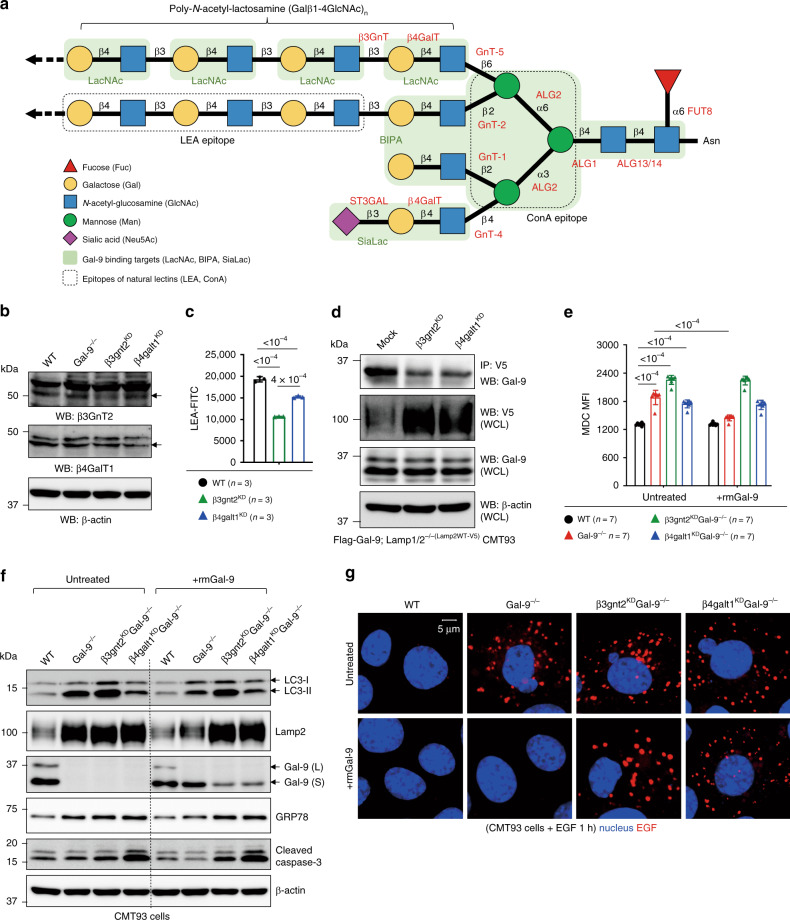
Interaction of Gal-9 with poly-LacNAc is critical for functionality of lysosome. **a** Schematic illustration of N-glycosylated moieties with the indicated colored carbohydrates. Specific enzymes required for each N-glycan synthesis step are shown in red. Gal-9 binding targets are indicated by shaded green area, while epitopes for natural lectin binding are indicated by dotted rectangle. β-1,3 N-acetyl-glucosaminyl-transferase (β3GnT) and β-1,4 galactosyl-transferase (β4GalT), key enzymes for the synthesis of poly-N-acetyl-lactosamine (poly-LacNAc), are indicated. **b** Western blot analysis of β3GnT2 and β4GalT1 proteins in gene-knockdown (KD) CMT93 cells. **c** Flow cytometry analysis of tomato lectin (LEA) binding efficiency to LacNAc in β3gnt2^KD^ and β4galt1^KD^ CMT93 cells. **d** Co-immunoprecipitation of Gal-9 and Lamp2 in β3gnt2 or β4galt1 gene-knockdown CMT93 cells. **e** Flow cytometry analysis of monodansylcadaverine (MDC^+^) autophagic vacuoles in the indicated CMT93 cells, cultured with or without exogenous recombinant mouse Gal-9 (rmGal-9). **f** Western blot analysis of autophagy, ER stress, and apoptosis markers in the indicated CMT93 cells, cultured with or without exogenous rmGal-9. **g** Immunofluorescence analysis of the indicated CMT93 cells, cultured with or without exogenous rmGal-9, for degradation of endocytosed fluorochrome-labeled EGF. Cells were pulsed with Alexa Flour 555-conjugated EGF for 5 min and confocal images were captured after 1 h post fixation. Data shown are representative or combined (**e**) results from two independent reproducible experiments. Statistical significance (*p* value) is indicated (**c**, **e**: Unpaired two-tailed *t*-test). Data are presented as mean ± SD. Source data are provided as a Source data file.

In the trans-Golgi network, newly synthesized lysosomal hydrolases are tagged with mannose-6-phosphate (M6P) groups exclusively to N-linked glycans in hydrolases. These M6P-tagged hydrolases are recognized by M6P receptor (M6PR) in the lumen of transport vesicles budding from the trans-Golgi network^50^. In this way M6P-tagged hydrolases are accurately transported to endolysosomal compartments, with the M6PR being trafficking in vesicles between the trans-Golgi network and these specialized compartments (Supplementary Fig. 5a)^51^. It was proposed that lysosomal enzymes are released from the receptor in the acidic lumen of these specialized M6PR/Lamp-enriched endosomes (so-called late endosomes or prelysosomes) and then subsequently packaged into mature lysosomes^51,52^. By density gradient ultra-centrifugation for subcellular fractionation of CMT93 cells^53^, we found M6PR is more localized in EEA1-enriched early endosomes but significantly less in Lamp2-enriched late endosomes/lysosomes (Supplementary Fig. 5b, c). As loss of M6PR has been associated with disrupted endolysosomal transport of hydrolases, decreased lysosome function, and impaired autophagy in cervical cancer cells^54^, we tested in CMT93 cells and found knockdown of M6PR (M6pr^KD^) indeed cause reduced activity of lysosomal hydrolases and impaired cargo degradation (Fig. 4f, g, Supplementary Fig. 5d), which were also found in Gal-9^−/−^ cells. However, while Gal-9 co-immunoprecipitates with M6PR, knockdown of M6PR does not affect lysosomal localization of Gal-9 (Fig. 4h), suggesting Gal-9 might indirectly associate with M6PR and is likely not involved in M6PR-mediated endolysosomal transport and cargo degradation. Due to technical difficulty in performing M6PR (>300 kDa) mutagenesis study in CMT93 cells (Supplementary Fig. 5e), we focused on Lamp2 and asked whether loss of Lamp1 and Lamp2 (Lamp1/2), two most abundant and heavily glycosylated lysosomal membrane proteins^7^, contributes to functionality of lysosome or autophagy. Similar to Gal-9^−/−^ but different from M6pr^KD^ cells, knockdown of Lamp1/2 (Lamp1/2^KD^) caused reduced Gal-9 endolysosomal/lysosomal localization (Fig. 5a, Supplementary Fig. 6a), increased LMP response (Fig. 5b), defective maturation and perinuclear mobilization of Rab7^+^CD63^+^ endosomes (Supplementary Fig. 6b), higher basal and tunicamycin-induced ER stress (Supplementary Fig. 6c), and impaired cargo degradation (Fig. 5c). These results indicate that binding of Gal-9 to glycosylated Lamp1/2 is essential for functionality of lysosome and autophagy. To gain more insights about how Gal-9-Lamp2 interaction regulates endolysosome/lysosome function at the molecular level, mutagenesis study was performed to abolish N-glycosylation by introducing single Asn(N)-to-Asp(D) mutation in each N-glycosylation site located in the N-terminal subdomain of Lamp2 (Supplementary Fig. 6d), which appears more accessible to Gal-9^6^. To this end, Lamp1/2 double knockout (Lamp1/2^−/−^) CMT93 cells were generated and then stably reconstituted with Flag-tagged Gal-9 and each of V5-tagged Lamp2 mutants (Fig. 5d, Supplementary Fig. 6e). While mutation of each N-glycosylation site in Lamp2 did not cause its overall protein instability (Supplementary Fig. 6f), binding of Gal-9 to Lamp2 was significantly reduced in Lamp2^N54D^ and Lamp2^N175D^ mutants (Fig. 5d). Notably, Lamp2^N175D^-reconstituted Lamp1/2^−/−^ cells also showed increased accumulation of MDC^+^ autophagic vacuoles (Fig. 5e)^28^, decreased binding to tomato lectin (LEA) which also binds to poly-LacNAc (Fig. 6a, Supplementary Fig. 6g)^55,56^, reduced lysosomal hydrolase activity, autophagy blockade, increased ER stress and apoptosis, as well as impaired cargo degradation (Fig. 5f–h). While Lamp2^WT^-reconstituted Lamp1/2^−/−^ CMT93 cells restored above defects identified in its parental Lamp1/2^−/−^ cells, Lamp2^N175D^-reconstituted cells failed to rescue them (Fig. 5f–h). Together, these results indicate that by binding to glycosylated moieties attached to Asn^175^ in Lamp2, Gal-9 maintains lysosome function to facilitate autophagy and prevent ER stress and associated cell apoptosis (summarized in Supplementary Fig. 5a).

### Gal-9 binding to poly-LacNAc moieties regulates autophagy

By X-ray structural analysis, Gal-9 was shown to bind to LacNAc, the biantennary pyridylaminated oligosaccharide (BIPA), and α2-3-sialyl-lactose (SiaLac) with high affinity (Fig. 6a)^57,58^. As aberrant poly-LacNAc expression is involved in gastric carcinoma or CRC,^59,60^, we asked whether in CMT93 cells, binding of Gal-9 to poly-LacNAc chains confers autophagy functionality. To this end, β1,3-N-acetyl-glucosaminyl-transferase (β3GnT2) and β1,4-galactosyl-transferase (β4GalT1)^56,61^, two key enzymes responsible for the synthesis of poly-LacNAc chains, were knockdowned (KD) by RNA interference (Fig. 6a, b). In β3gnt2^KD^ and β4galt1^KD^ CMT93 cells, binding of tomato lectin (LEA) to LacNAc and binding of Gal-9 to Lamp2 were indeed decreased (Fig. 6c, d)^56^, which likely led to autophagy blockade, as evidenced by increased accumulation of Lamp2 and MDC^+^ autophagic vacuoles (Fig. 6d, e). Notably, while exogenous Gal-9 effectively reduced MDC^+^ autophagic vacuoles in Gal-9^−/−^ cells, it failed to do so in β3gnt2^KD^ or β4galt1^KD^ cells (Fig. 6e), suggesting the interaction of Gal-9 with poly-LacNAc chains is functionally linked to autophagy. Similarly, exogenous Gal-9 also failed to rescue defects of autophagy block (measured by LC3, Lamp2), ER stress (measured by GRP78), apoptosis (measured by cleaved caspase-3), and cargo degradation in β3gnt2^KD^ or β4galt1^KD^ CMT93 cells (Fig. 6f, g). Notably, similar to α-lactose pre-treatment, endocytosed recombinant Gal-9 within cells was significantly decreased in the absence of β3GnT2 or β4GalT1 (Fig. 6f). Together we conclude binding of Gal-9 to poly-LacNAc glycan chains contributes to lysosomal function of Gal-9 and confers lysosome-mediated autophagy and cargo degradation.

### Gal-9 regulates lysosome function to prevent pancreatiits

**Fig. 7:**
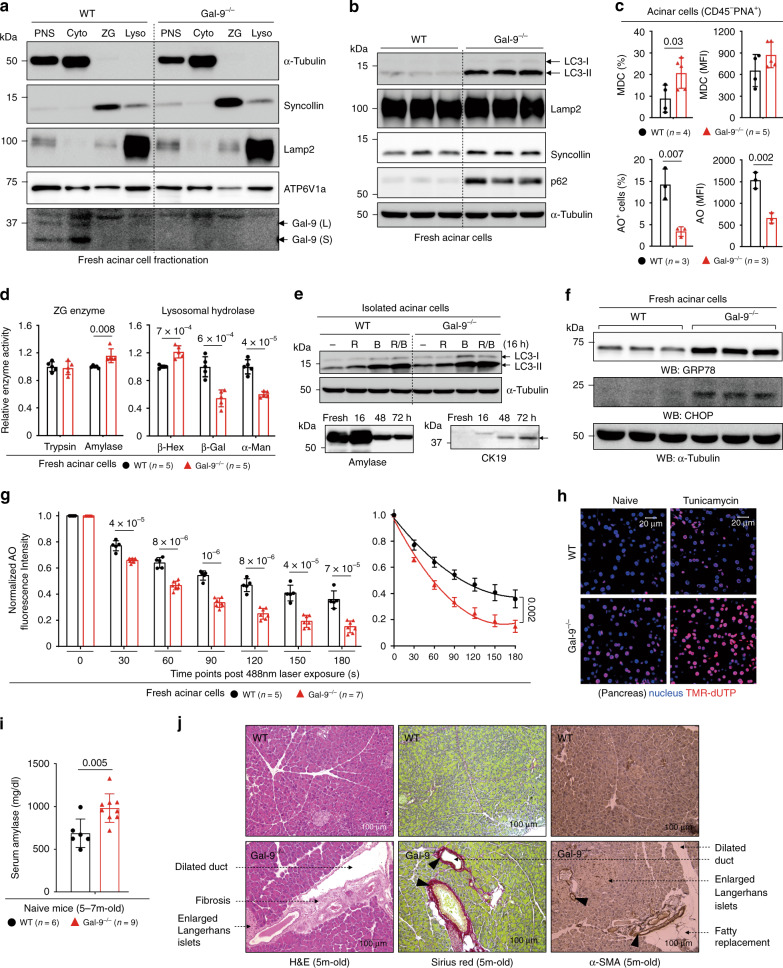
Gal-9 regulates lysosomes in acinar cells to prevent pancreatic disorders. **a** Western blot analysis of subcellular fractionations for localization of Gal-9 in fresh acinar cells. Cytosol marker α-tubulin, ZG marker syncollin, and lysosome marker Lamp2 are indicated. PNS: post nuclear supernatant, Cyto: cytosol fraction, Lyso: lysosome fraction. **b** Western blot analysis of autophagy markers in fresh acinar cells. **c** Flow cytometry analysis of fresh acinar cells, stained with monodansylcadaverine (MDC) to detect autophagic vacuoles or with acridine orange (AO) to detect acidic compartments. **d** Activity of ZG digestive enzymes or lysosomal hydrolases in fresh acinar cells was determined by specific substrates. **e** Western blot analysis of autophagy flux by the LC3 marker in acinar cells, treated with rapamycin (R), bafilomycin (B), or both (R/B) for 16 h. **f** Western blot analysis of ER stress markers in fresh acinar cells. **g** The indicated AO-labeled acinar cells were exposed to a 488 nm laser to induce lysosomal damage. Confocal images after laser exposure were taken at the indicated time points. Loss of lysosome stability was determined by the decrease of AO red fluorescence over time which was normalized to cells exposed at 0 s. **h** Immunofluorescence of pancreatic tissue sections from naïve or tunicamycin-injected mice at day-2 for TUNEL (TMR-dUTP) analysis. **i** Serum amylase levels were determined by Fuji Dri-Chem Clinical Chemistry Analyzer FDC 4000i.m. **j** Pancreatic tissues from aged mice were analyzed by H&E (left panels) for acini injury, and by Sirius red (middle panels) and α-SMA staining (right panels) for fibrosis. Data shown are representative or combined (**d**, **i**) results from two independent reproducible experiments. Statistical significance (*p* value) is indicated (**c**, **d**, **g**, **i**: Unpaired two-tailed *t*-test). Data are presented as mean ± SD. Source data are provided as a Source data file.

Dysregulation of lysosome function, autophagy, and ER stress in acinar cells has been linked to pancreatic inflammation or fibrosis^10,31,62^. As Gal-9 is involved in pancreatic carcinoma and Lamp2 is also associated with pancreatitis^10,26,63^, we investigated whether Gal-9 has a function in the homeostasis of pancreatic acinar cells, which, similar to intestinal Paneth cells, are an autophagy-active secretory cell type. By acinar cell fractionation and immunofluorescence (Fig. 7a, Supplementary Fig. 7a)^64^, we found under the physiological condition, Gal-9 is weakly expressed in amylase-expressing acini and is detectable in Lamp2-enriched lysosomes but not in syncollin-enriched zymogen granules (ZG). Similar to ileum crypts, Gal-9^−/−^ acinar cells exhibited increased accumulation of LC3, Lamp2, p62, and MDC^+^ autophagic vacuoles, as well as higher lysosomal pH with decreased AO/LysoTracker staining (Fig. 7b, c, Supplementary Fig. 7b), indicative of lysosome dysfunction and autophagy blockade. Impairment of lysosome-associated autophagy in Gal-9^−/−^ acinar cells was further supported by decreased lysosomal hydrolase activity (Fig. 7d) and impaired autophagy flux (Fig. 7e)^44^. Lysosome dysfunction was also observed under transmission electron microscopy (Supplementary Fig. 7c), showing aberrant accumulation of large autophagic vacuoles with partially digested materials which is reminiscent of Lamp1/2^−/−^ mice^65^. Notably, levels of amylase and trypsinogen in ZG were not altered in Gal-9^−/−^ acinar cells (Supplementary Fig. 7d)^64^, suggesting it was not over-activation of amylase or trypsin that caused pancreas injury. Similar to ileum crypts, Gal-9^−/−^ acinar cells also showed increased ER stress at the steady state and higher LMP response after photo-oxidation challenge (Fig. 7f, g)^30^, which were likely leading to increased basal or tunicamycin-induced apoptosis (Fig. 7h, Supplementary Fig. 7e)^46^, and eventually pancreas injury with elevated serum amylase, ductal dilation, enlarged islets of Langerhans, acini vacuolization, and fibrosis in mice (Fig. 7i, j, Supplementary Fig. 7f, g)^31,66,67^. Therefore, in addition to intestinal Paneth cells, Gal-9 is essential for maintenance of functional lysosomes and autophagy in pancreatic acinar cells, which is required to alleviate ER stress and prevent tissue injury or aberrant induction of fibrosis.

## Discussion

Our work here reveal a fundamental and unexplored function of Gal-9 in lysosomal lumen at the steady state in promoting autophagy and protecting cells from ER stress-associated LMP and cell death, especially in autophagy-active secretory cells. At the molecular level, we identified N-glycosylated M6PR and Lamp2 are major lysosomal binding proteins of Gal-9 in gut epithelial cells. It appears that the Gal-9-Lamp2 interaction contributes to endolysosome/lysosome function and cargo degradation. We also reveal that binding of Gal-9 to N-glycosylated Asn^175^ in Lamp2 is critical for functionality of autophagy under the physiological conditions without endolysosomal/lysosomal damage^15^. We also provide evidence that Gal-9-mediated autophagy is indeed through its binding to poly-LacNAc chains in N-glycosylated targets. At the cellular level, we found Gal-9 preferentially targets intestinal Paneth cells and pancreatic acinar cells, and promotes autophagic degradation to resolve their ER stress and prevent cell death. At the global level in mice, we found lysosome dysfunction, due to loss of Gal-9, could serve as a shared cell-intrinsic defect in the intestine and pancreas that renders mice more susceptible to stress or organ pathogenesis. Therefore, highly secretory cells are homeostatically being protected from ER stress and apoptosis via Gal-9-mediated autophagy that otherwise could lead to tissue inflammation or injury^31^. Our study not only provides clues why Gal-9, via targeting and regulating autophagy in Paneth cells, is a contributing risk factor for CD, but also supports that IBD and extraintestinal manifestations (EIMs), such as pancreatitis in this study, are pathologically linked^25,68^. We therefore propose that lysosome and autophagy are cross-regulated, so patients with IBD or lysosomal/glycogen storage disease (LSD/GSD) are prone to have clinical manifestations simultaneously in multiple tissues or organs^1,5,69–71^.

One key finding in our study is to reveal how a single N-glycosylation site (Asn^175^) in Lamp2 could have a predominant role in affecting lysosome function, autophagy, and ER stress. Unexpectedly, mutation of N-glycosylation sites in Lamp2 could cause increased, decreased, or unaltered binding of Lamp2 to Gal-9, suggesting in Lamp2 each N-glycosylation chain, either bound or unbound by Gal-9, might have a unique role in maintaining or affecting structural conformation that is consequently associated with protein-protein (Gal-9-Lamp2) interaction or functionality of Lamp2 in lysosome and autophagy. That is, the overall contribution of N-glycosylation to functionality of Lamp2 is the result of delicate and complicated structural positioning of each glycosylated chain, which will also be further affected by Gal-9 binding. As such, loss of one N-glycosylation site, while not affecting the overall binding of galectin, may still cause conformational change leading to protein dysfunction. In the case of Lamp2, Asn^175^ is close to the linker region (amino acid 189-233) which connects N- and C-terminal subdomains of Lamp2 (Supplementary Fig. 6d)^6^. It is possible that loss of N-glycosylation in Asn^175^ affects the hinge function of linker peptides that consequently causes an unfavorable conformational change between two subdomains leading to Lamp2 dysfunction. Likewise, Asn^175^ in Lamp2 may affect the formation of Cys^149^-Cys^185^ disulfide bond, which was shown to stabilize the conserved β-prism fold found in the subdomains of Lamp protein family^72^. Furthermore, it was proposed that not all glycosylation sites in Lamp2 can be accessible to β1,3-N-acetyl-glucosaminyl-transferase for glycan modification^73^. As structural features might determine the addition of poly-LacNAc glycans which then serve as binding targets for Gal-9, it is therefore difficult to distinguish whether it is the poly-LacNAc chain itself or Gal-9-bound poly-LacNAc chain that actually contributes to the functionality of Lamp2. In addition, how many LacNAc moiety will be synthesized and added to a single N-glycosylation site is completely unclear. However, in our study, we show that poly-LacNAc glycans are indeed required for functionality of Gal-9, as exogenous recombinant Gal-9 fails to rescue autophagy defects in Gal-9^−/−^ cells in the absence of β3GnT2 or β4GalT1 transferase.

It is technically challenging to prove that it is solely the Gal-9-Lamp2 interaction that regulates lysosome function, as the binding of Gal-9 to N-glycans in target proteins has no sequence specificity. Correlated to this, it is still unclear why in CMT93 cells other endogenous galectins, whose expression is more abundant than Gal-9, could not functionally rescue those defects in Gal-9^−/−^ cells. However, our mass spectrometry analysis reveals that at least in CMT93 colon epithelial cells, Lamp2 is the predominant regulatory target for Gal-9 with respect to endolysosome/lysosome function and our results support this conclusion. Phenotypically, similar to Lamp2^−/−^ mice^63,65^, Gal-9^−/−^ mice also exhibited large autophagic vacuoles with partially digested materials in pancreas, decreased acinar cell homeostasis, and spontaneous pancreatitis. In Gal-9^−/−^ and Lamp2^−/−^ CMT93 cells, endolysosome/lysosome function and cargo degradation were impaired, indicating a causual effect mediated by the specific interaction of Gal-9 to Lamp2. In humans, while Gal-9 polymorphism is associated with CD^25^, Lamp2 deficiency causes Danon disease with cardiomyopathy^12^. It is possible that differential expression patterns of Gal-9 and Lamp2 in different cell types offset the requirement of their functions or there are compensatory mechanisms for Lamp2-mediated function when endolysosomal/lysosomal Gal-9 levels are low in certain cells. Interestingly, while intestinal disorders in either spontaneous or experimental models have not been reported in Lamp2^−/−^ mice, impaired phagosome maturation and acidification in Lamp2^−/−^ neutrophils were shown to cause periodontitis in mice^74^, an oral inflammatory EIMs commonly reported in IBD patients^75^. Therefore, phenotypical correlations in Gal-9 and Lamp2 deficiency with respect to IBD, pancreatitis, and EIMs might support Gal-9-Lamp2 axis as a potential therapeutic target for these disorders and therefore is worth further investigation in animal models or human subjects.

## Methods

### Animals

*Galectin-9*^−/−^ mice were obtained from Dr. Liu, Fu-Tong, Institute of Biomedical Sciences, Academia Sinica, Taiwan. *Galectin-9*^−/−^ mice were in C57BL/6 background and we have further backcrossed *Galectin-9*^−/−^ mice to C57BL/6 mice for two more generations before intercrossing them to have littermates for our experiments. *Lgr5-eGFP-ires-creERT2* mice were obtained from Jackson Laboratory. *Lgr5-eGFP* mice were bred with *Galectin-9*^−/−^ mice to obtain *Lgr5-eGFP*^*+*^*Galectin-9*^−/−^ mice. *Galectin-9* conditional knockout (*Galectin-9*^*flox/flox*^) mice in C57BL/6 background were generated via Transgenic Mouse Core Facility in National Taiwan University by the CRISPR/Cas9. To minimize off-target effects in CRISPR mice, *Galectin-9*^*flox/flox*^ mice were first backcrossed to C57BL/6 mice for four generations before breeding into Paneth cell-specific *Galectin-9* conditional knockout mice (*Defa6-Cre*^+^*Galectin-9*^*flox/flox*^). The *Defa6-Cre* mice were obtained from Dr. Richard S. Blumberg, Division of Gastroenterology, Department of Medicine, Brigham and Women’s Hospital, Harvard Medical School. Animals were maintained in a specific-pathogen-free (SPF) facility at a relative humidity 50 ± 10%, 20–26 °C, and in 12 h dark/light cycles (08:00–20:00 light). Experiments were performed on mature animals (age, 8–12 wk) in both male and female mice, unless otherwise indicated. Animal care and experimental protocols (Protocol ID: 15-12-908) have been approved by the Institutional Animal Care and Use Committee (IACUC) at the Institute of Biomedical Sciences, Academia Sinica. Ethical compliance has been observed in the animal study. Dr. John T. Kung is the chair of IACUC and Ethics Committee in Academia Sinica.

### Antibodies

The following primary antibodies were used for immunofluorescence, immunochemistry or immunoblotting: rabbit anti-Amylase [aa484-511] (LSBio LS-B11116) (IB: 1:500; IF: 1:200), rabbit anti-ATP6V1A [EPR19270] (Abcam #199326) (IB: 1:2000), mouse anti-β-actin [C4] (Santa Cruz #sc-47778) (IB: 1:1000), rabbit anti-Calreticulin [EPR3924] (Abcam #ab92516) (IB: 1:5000, IF: 1:100), rabbit anti-Syncollin [EPRR13148] (Abcam #ab178415) (IB: 1:2000), rabbit anti-Cathepsin B [EPR21033] (Abcam #ab214428) (IB: 1:1000), rabbit anti-Cathepsin D [EPR3057Y] (Abcam #ab75852) (IB: 1:2000; IF: 1:100), rat anti-CD63 [NVG-2] (BioLegend #143902) (IF: 1:100), rat anti-Galectin-9 [108A2] (BioLegend #137901) (IB: 1:1000; IF: 1:100), rabbit anti-GFP [D5.1] (Cell Signaling Technology #2956) (IF: 1:100), rabbit anti-GRP78 (Abcam #ab21685) (IB: 1:3000; IF: 1:800), rabbit anti-Ki67 [SP6] (Abcam #ab16667) (IF: 1:250), rat anti-Lamp1 [1D4B] (Abcam #ab25245) (IB: 1:1000), rat anti-Lamp2 [ABL-93] (Abcam #ab25339) (IB: 1:1000; IF: 1:100), rabbit anti-Lamp2 (Thermo Fisher #PA1-655) (IF: 1:200), rabbit anti-LC3B [D11] (Cell Signaling Technology #3868) (IB: 1:1000; IF: 1:200), mouse anti-Lysozyme [BGN/06/961] (Abcam #ab36362) (IF: 1:100), rabbit anti-MMP7 (Abcam #ab5706) (IF: 1:100), mouse anti-p62 (Abcam #ab56416) (IB: 1:2000), rabbit anti-Rab7 [D95F2] (Cell Signaling Technology #9367) (IF: 1:200), rabbit anti-α-tubulin (Cell Signaling Technology #2144) (IB: 1:1000), mouse anti-Chop [L63F7] (Cell Signaling Technology #2895) (IB: 1:1000), rabbit anti-Cleaved Caspase-3 (Asp175) [5A1E] (Cell Signaling Technology #9664) (IB: 1:1000), rabbit anti-XBP1 [EPR22004] (Abcam #ab220783) (IB: 1:1000), mouse anti-Flag [M2] (Sigma-Aldrich #F1804) (IP: 5ug antibody/1 mg dynabeads), mouse anti-V5 tag [SV5-Pk1] (Abcam #ab27671) (IP: 5ug antibody/1 mg dynabeads; IB: 1:1000), goat anti-β3gnt2 (MyBiosource #MBS421555) (IB: 1:1000), rabbit anti-β4galt1 (MyBiosource #MBS8242969) (IB: 1:1000), goat anti-human galectin-9 (R&D Systems #AF2045) (IB: 1:1000), rabbit anti-human galectin-9 (Sigma #HPA046876) (IHC: 1:100), rabbit anti-M6PR (cation independent) [EPR6599] (Abcam #ab124767) (IB: 1:10,000), and rabbit anti-Niemann Pick C1 antibody [EPR5209] (Abcam #ab134113) (IB: 1:2000), goat anti-CK19 (Santa Cruz #sc-33111) (IB: 1:1000; IHC: 1:1000), rabbit anti-α-SMA (Abcam #ab32575) (IHC: 1:200), rabbit anti-EEA1 (C45B10) (Cell Signaling Technology #3288) (IB: 1:1000), rabbit anti-Syntaxin 6 antibody [EP7665] (Abcam #ab140607) (IB: 1:5000), rabbit Anti-Rab5 antibody [EPR21801] (Abcam #ab218624) (IB: 1:1000). Fluorophore-conjugated secondary antibodies (all 1:300 dilution used) for immunofluorescence were purchased from Cell Signaling Technology unless otherwise mentioned. The following secondary antibodies were used: anti-mouse Alexa Fluor-594 (#8890), anti-rabbit Alexa Fluor-488 (#4412), anti-rabbit Alexa Fluor-594 (#8889), anti-rat Alexa Fluor-488 (#4416), and anti-rat Alexa Fluor-594 (Thermo Scientific #A-11007). Horseradish peroxidase-conjugated secondary antibodies (all 1:10,000 dilution used) for immunoblotting were purchased from Jackson ImmunoResearch Laboratories, Inc. The following secondary antibodies were used: anti-mouse-HRP (#115-035-003), anti-rabbit-HRP (#111-035-003), and anti-rat-HRP (#112-035-003). The following antibodies were used for flow cytometry: rat anti-CD24 [M1/69] APC-eFluor 780 (eBioscience #47-0242-82) (1:200), rat anti-CD45 [30-F11] Alexa Fluor-700 (eBioscience #56-0451-82) (1:400), rat anti-CD326 (EpCAM) [G8.8] eFluor 450 (eBioscience #48-5791-82) (1:400), rat anti-CD44 [IM7] PECy7 (BioLegned #103029) (1:200), rabbit anti-active Caspase-3 [C92-605] Alexa Fluor-647 (BD Biosciences #560626) (5ul/test), anti-Ki67 [SolA15] PeCy7 (eBioscience #25-5698-82) (1:200), rabbit anti-Lysozyme (EC 3.2.1.17) FITC (1:100, Dako #F037201-1) (1:100), rat anti-mouse Galectin-9 [RG9-35] APC (BioLegned #136110) (1:200), anti-PNA (Vector Laboratories #FL-1071) (1:200), and DyLight 488 Labeled Lycopersicon Esculentum (Tomato) Lectin (Vector Laboratories #DL-1174-1) (5ug/ml). Flow cytometry data were collected by BD LSRII with BD FACS DIVA Software (v6.1.3) and analyzed by FlowJo (v10.0.7).

### Intestinal crypts isolation

To isolate the crypts, mice (8-12 weeks old) were euthanized with CO_2_ and the abdomen was cut open to separate ~12 cm of distal small intestine (whole Ileum) and ~5 cm of distal large intestine (distal colon). The Peyer’s patches and fat tissue were removed and organs were cut opened longitudinally and washed with PBS. The colon was scratched with cover slip several times to remove the epithelial surface. The intestines were cut into 2 cm pieces and then placed into 50 ml falcon tubes and washed vigorously by shaking in Hank’s Balanced Salt Solution (HBSS) until the supernatant was clear. To dissociate the crypts, intestinal fragments were further cut into 5 mm pieces and then placed into 50 ml tube containing 2 mM EDTA (for ileum) or 5 mM EDTA (for colon) in chelation buffer [5.6 mM Na_2_HPO_4_, 8.0 mM KH_2_PO_4_, 96.2 mM NaCl, 1.6 mM KCl, 43.4 mM Sucrose, 54.9 mM D-Sorbitol, 0.5 mM DL-Dithiothreitol (DTT), 25 mM HEPES, 100 U/ml penicillin/100 μg/ml streptomycin, and 50 μg/ml Gentamicin] and shaked at 100 rpm in room temperature for 5 min. The chelation buffer was aspirated and replaced with cold wash buffer [1× PBS pH 7.4, 1× penicillin/streptomycin, 50 μg/ml Gentamicin, and 0.1% bovine serum albumin (BSA)] and shaked vigorously with hand twenty times to remove villi and epithelial debris. This step was repeated and then intestinal fragments were again transferred to a fresh 50 ml falcon tube containing EDTA in cold chelation buffer and incubated at 4 °C on a benchtop roller with 25 rpm shaking for 30~40 min. Later, the supernatant was aspirated and then 10 ml cold wash buffer was added to the intestinal fragments and vortexed for 10 s. The supernatant containing crypts was collected and transferred to a fresh 50 ml falcon tube pre-coated with 5% FBS. This step was repeated and each successive fraction was collected and examined underneath the phase-contrast microscope for the presence of intact intestinal crypts and lack of villi. The fractions with intact ileal crypts were pooled together and filtered by passing through 70 μm cell strainer (BD Biosciences #352350) into 50 ml falcon tube pre-coated with 5% FBS to remove any debris and this step was not required for the colon crypts. The crypts were pelleted by centrifuge at 300 × *g* for 5 min at 4 °C. To remove single cell contamination from the heavier epithelial crypts, the supernatant was aspirated and crypts pellet was again resuspended in 10 ml cold wash buffer and centrifuged at 20 × *g* for 5 min at room temperature. The supernatant was again aspirated and crypt pellet was resuspended in 1~2 ml wash buffer, 100 μl was placed on a petri dish and the crypts were counted under phase-contrast microscope.

### Ileal organoid culture

To culture mouse ileal organoids, a total of ~500 freshly isolated ileal crypts per well were mixed with 50 μl basement membrane matrix growth factor reduced Matrigel (BD Biosciences #356231) containing 1 μM Jagged-1 peptide (Notch Agonist) (AnaSpec #AS-61298) and plated in 24-well plates. The Matrigel was polymerized for 10 min at 37 °C incubator, and 500 μl ENR growth media was added on top of the Matrigel. The ENR growth medium was prepared by mixing basal culture medium (BCM) [advanced DMEM/F-12 supplemented with 100 U/ml penicillin/100 μg/ml streptomycin, 50 μg/ml Gentamicin, 2 mM GlutaMAX, 10 mM HEPES (Gibco #15630-080), 1 mM *N*-acetyl-L-cysteine (Sigma #A9165), 1× B-27 Supplement (Gibco #17504-044), 1× N-2 Supplement (Gibco #17502-048), and 1% BSA (Gibco #15260-037)] with NR conditioned medium in 3:1 ratio, and 50 ng/mL murine Epidermal Growth Factor (mEGF) (Gibco #PMG8041). To maximize early growth of developing primary organoids from crypts, the ENR growth medium was supplemented with murine 5 ng/ml Wnt-3A (Peprotech #315-20), 10 μM rho-associated protein kinase (ROCK) inhibitor, Y-27632 (Sigma #Y0503), and 0.5 μM transforming growth factor (TGF)-β type I receptor inhibitor, A-83-01 (R&D Systems #2939) for the first 2 days of the culture. Media was replaced every 2 days. Along with medium changes, treatment wells received 0.5 μg/ml recombinant mouse galectin-9 protein (R&D Systems #3535-GA) for 7 days or 100 ng/ml tunicamycin (Sigma #T7765) for 16 h on day 6 or 100 ng/ml recombinant mouse IL-22 protein (eBioscience #14-8221-63) for 24 h on day 6 or either combination of galectin-9 and tunicamycin or galectin-9 and IL-22.

### Tunicamycin-induced ER stress

For in vivo tunicamycin experiments, mice were intraperitoneally injected with tunicamycin (2 mg/kg body weight) (Sigma #T7765), diluted in DMEM containing 3% fetal calf serum and 2% penicillin/streptomycin. Survival was monitored over 96 h. To examine ER stress-induced apoptosis, isolated acinar cells, ileum crypts, or tissue sections collected on day 2 after tunicamycin injection were examined by immunofluorescence or flow cytometry. Cell death analysis by TUNEL assay (In Situ Cell Death Detection Kit, TMR red, Roche #12156792910) was performed on tissue sections. In vitro tunicamycin experiments were examined using CMT93 cells. Cells were treated with 1 μg/ml tunicamycin for indicated time and ER stress, LMP, and apoptosis responses were analyzed by Western blotting or flow cytometry.

### DSS-induced colitis

For colitis studies, mice were given 2% dextran sodium sulfate (DSS, MP Biologicals, molecular mass 36–40 kDa) in drinking water for the experimental days 1–5 followed by normal drinking water until the end of the experiment (day 8). Colonoscopy images were captured on day 5 and day 8 by using endoscopy system (TESALA AVS, Olympus, Tokyo, Japan).

### EGF degradation assay

Epidermal growth factor (EGF), when binding to its receptor (EGFR), sends downstream signals and eventually the ligand-receptor complex is internalized into lysosomes through endocytosis and is digested by lysosomal enzymes. Any defects in lysosomes will result in curtailing the ligand-receptor degradation. To check the EGF degradation in CMT93 cells, cells were grown on sterile silane coated glass slides for 24~48 h with or without rmGal-9 treatment. The cells were kept on ice for 10 min and washed with cold wash solution (Live Cell Imaging Solution containing 20 mM Glucose and 1% BSA) to inhibit endocytosis. Biotinylated EGF-Alexa Fluor 555 streptavidin (Molecular probes #E35350) was loaded on to the cells at a concentration of 2 μg/ml in wash solution and incubated at 37 °C for 5 min. After that cells were washed with warm wash solution and incubated in complete DMEM for 1 h at 37 °C and then fixed with 4% formaldehyde and later imaged. For live imaging, cells were grown on µ-Dish 35 mm, high (ibidi #81156) upto 80% confluency with or without rmGal-9. To visualize lysosomes, cells were stained with 50 nM LysoTracker™ Deep Red (Molecular probes #L12492) for 15 min, followed by 50 μg/ml pHrodo™ Green Dextran (Molecular probes #P35368) for 10 min to visualize endosomes. Later, cells were loaded with EGF-Alexa Fluor 555 for 5 min and washed with PBS and changed to Live Cell Imaging Solution containing 20 mM Glucose and 1% BSA, immediately imaged with Carl Zeiss LSM 780 imaging system under a 60x oil-immersion objectives. This time point was considered as 0 h, and after 1 h the same area was imaged and considered as 1 h.

### Lysosomal stability assay

The isolated pancreatic acinar cells, ileal crypts, or cultured CMT93 cells, treated with or without 0.5 μg/ml recombinant mouse galectin-9 for 48 h, were labeled with AO in the growth medium (1:10,000 dilution from the 2% AO stock). The cells were incubate at 37 °C for 20 min and then washed twice with live imaging solution (Invitrogen #A14291DJ). Live imaging solution was added to cover the cells in a confocal dish and immediately proceeded for microscopic analysis. Groups of cells in the brightfield (eight pre-defined areas of each well) were identified in the brightfield and lysosomes (visible as black dots) were put in focus very rapidly to avoid any damage to lysosomes since the brightfield also contains blue light. The cells were immediately and continuously exposed to 489-nm light from a 100 mW diode laser while laser scanning micrographs where captured every 484 ms on a Zeiss LSM780 confocal microscope system in two channels defined by secondary bandpass filters for 495–555 nm (green) and LP650 nm (Red) light for 180 s. Loss of lysosome integrity in cells was visualized by the loss of red fluorescence, which is normalized to unexposed cells.

### Hydrolase activity assay

β-Hexosaminidase, β-galactosidase, and α-mannosidase activity was assayed by estimation of p-nitrophenol liberation of p-nitrophenyl-N-acetyl-β-D-glucosaminide (Sigma #N9376), 4-Nitrophenyl-β-D-galactopyranoside (Sigma #N1252), and 4-nitrophenyl-α-D-mannopyranoside (Sigma #N2127), respectively. The assay mixture consisted of 5 mM substrate, 50 mM sodium citrate buffer pH 4.5, 0.1 % Triton X-100, 0.4 % BSA, and enzyme solution in a final volume of 0.2 ml. For β-Hexosaminidase activity, 10 μg cell extract was added to the assay mixture and incubate for 1 h at 37 °C. Reaction was stopped by addition of 1.0 ml of 0.4 M glycine/NaOH buffer, pH 10.4. The absorbance of liberated p-nitrophenol was measured at 405 nm. For β-galactosidase and α-mannosidase activity, 200 μg cell extracts were added to the assay mixture and incubate for 16 h at 37 °C. Reactions were stopped by addition of 1.0 ml of 0.4 M glycine/NaOH buffer, pH 10.4. The absorbance of liberated p-nitrophenol was measured at 515 nm.

### Subcellular fractionation of CMT93 cells

A near confluent monolayer of CMT93 cells (3 × 10^8^ cells) were serum starved for 2 h before homogenization to increase the lysosomal mass. The cells were rinsed two times with PBS and then washed twice with wash medium (0.25 M sucrose, 10 mM Tris-HCl) at room temperature. The wash medium is replaced with ice-cold Homogenization Media (HM) containing 0.25 M sucrose, 1 mM EDTA, 10 mM Tris-HCl, 1× Protease inhibitors at pH 7.4 (*ρ* = 1.030 g/ml), and the cells were scrapped with cell lifter (Corning #3008) into HM. The monolayer was pelleted and homogenized in 1 ml of HM by using 25 strokes of the pestle of a tight-fitting Dounce homogenizer. To ascertain the degree of cell breakage, they were checked under a microscope using Trypan Blue staining solution. After 90% of cell breakage, they were centrifuged at 2600 × *g* for 10 min at 4 °C to remove large cell debris and nuclei. The supernatant is post-nuclear supernatant (PNS), transferred to a new centrifuge tube and centrifuged at 20,000 × *g* for 20 min at 4 °C to pellet down the crude lysosomal fraction, and the supernant is cytosolic fraction. The crude lysosomal fraction consisting of mitochondria, lysosomes, endosomes, and peroxisomes were washed three times with HM, and the cytosolic fraction was cleared by further centrifuging in a Ultra-Clear centrifuge tubes (Beckman Coulter #344057) at 150,000 × *g* for 1 h at 4 °C in Beckman Coulter Optima MAX-XP Ultracentrifuge using TLA 100.3 S/N. 08U 3870 rotor to obtain light membrane fraction (LM) consisting of golgi apparatus and endoplasmic reticulum. Cells pre-treated with or without 50 mM α-Lactose for 2 h are incubated with rmGal-9 for another 2 h. The fractions are lysed in 1% NP-40 lysis buffer containing 1× Protease Inhibitor Cocktail and analyzed by western blotting.

### Pancreatic acinar cell isolation and culture

To isolate pancreatic acinar cells, mice were euthanized and pancreas were collected and immediately washed with ice-cold HBSS twice. Pancreas was transferred to an eppendorf, well-minced, and then transferred into a 50 ml tube. Centrifuged for 2 min at 450 × *g*. The supernatant was discarded to remove cell debris and blood cells. 5 ml of collagenase IA solution (HBSS containing 10 mM HEPES, 200 U/ml collagenase IA (Sigma #C2674), and 0.25 mg/ml of trypsin inhibitor (Sigma #T6522) was added and incubated for 20 min at 37 °C with 200 rpm shaking. The enzymatic reaction was stopped by adding 10 ml of cold HBSS with 5% FBS and then centrifuged for 2 min at 450 × *g*, 4 °C. The supernatant was aspirated to remove collagenase IA solution. Cell pellets were resuspended, washed twice with 10 ml of cold HBSS with 5% FBS and filtered through a 100 μm strainer (Falcon) to remove non-digested materials. The cells were suspended in Waymouth’s medium and transferred to 6-well plate and placed for 24 h in a 37 °C humidified 5% CO_2_ incubator. Purity of acinar cells was examined by PNA staining (Vector Laboratories, #FL-1071) via flow cytometry as described. To avoid acinar cells undergoing acinar-to-ductal metaplasia, fresh acinar cells were used in most of the experiments. If acinar cells were to be treated for 16 h, level of ductal cell marker CK19 was measured.

### Statistics

All statistical analyses were performed using GraphPad Prism v7.1 software. The results are expressed as the mean ± S.D. Statistical significance (*p* value) is indicated. Unpaired two-tailed *t*-test is performed unless otherwise indicated. The definition of symbols or sample numbers (*n*) in each figure for statistical analysis is provided in details in the section of “Reproducibility” in Supplementary methods.

### Reporting summary

Further information on research design is available in the Nature Research Reporting Summary linked to this article.

## Supplementary information

Supplementary information

Peer Review File

Supplementary Data 1

Reporting Summary

## Data Availability

UniProtKB Mus musculus protein database (https://www.uniprot.org/) was used for mass spectrometry analysis. The heatmap (Fig. 4d) and volcano plot analysis (Supplementary Fig. 4h), based on the online dataset (mouse lysosomal protein coding gene, mLGdb v.1.2, http://lysosome.unipg.it), were created and uploaded to a public database (Accession code: MassIVE MSV000085831). Colored Fig. 6a was created and partially adapted from non-colored Fig. 1 (10.1038/s41598-018-25580-9) by Chung-Geun Lee et al. We acknowledge the original authors’ contribution and indicate that their work is published under a CC BY 4.0 license. All uncropped Western blots are provided in the Supplementary information. All data presented in this study are available to the public upon request via the corresponding author. Source data are provided with this paper.

## Source data

Source Data
