# Supplementary material for: Lumenal Galectin-9-Lamp2 interaction regulates lysosome and autophagy to prevent pathogenesis in the intestine and pancreas
Source: Nat Commun. 2020 Aug 27;11:4286. doi: 10.1038/s41467-020-18102-7 (PMC7453023; doi:10.1038/s41467-020-18102-7)

Lumenal Galectin-9-Lamp2 interaction regulates lysosome and autophagy to prevent pathogenesis in the intestine and pancreas

Supplementary Information

Sudhakar, et al.

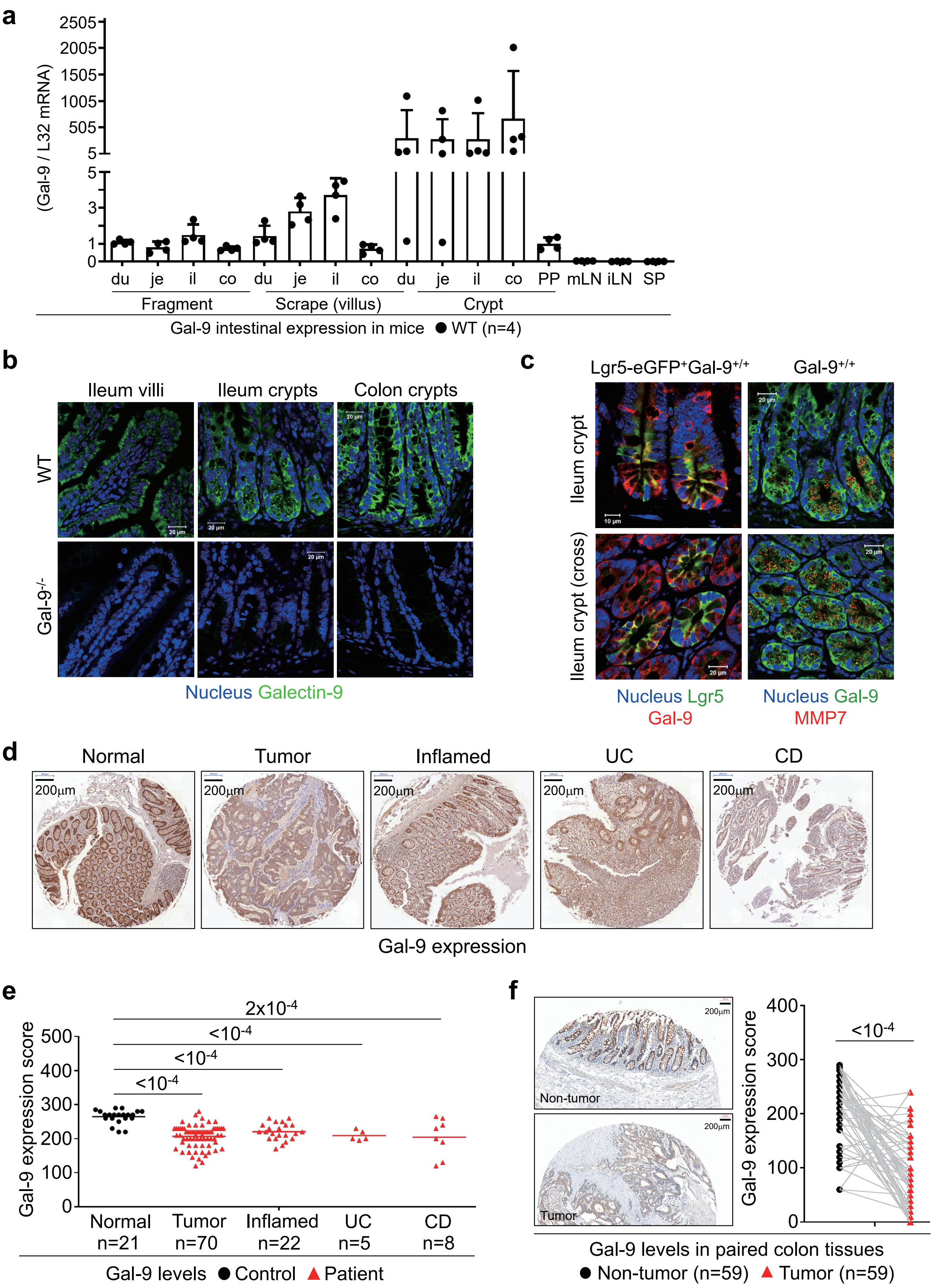
a
(Gal-9 / L32 mRNA)
2505
2005
1505
1005
505
5
5
3
1
0
du je il co du je il co du je il co PP mLN iLN SP
Fragment
Scrape (villus)
Crypt
Gal-9 intestinal expression in mice ● WT (n=4)
b
Ileum villi
Ileum crypts
Colon crypts
WT
Gal-9-/-
Nucleus Galectin-9
c
Lgr5-eGFP+Gal-9+/+
Gal-9+/+
Ileum crypt
Ileum crypt (cross)
Nucleus Lgr5
Gal-9
Nucleus Gal-9
MMP7
d
Normal
Tumor
Inflamed
UC
CD
200µm
Gal-9 expression
e
Gal-9 expression score
2x10-4
<10-4
<10-4
<10-4
Normal n=21
Tumor n=70
Inflamed n=22
UC n=5
CD n=8
Gal-9 levels ● Control ▲ Patient
f
Non-tumor
Tumor
200µm
Gal-9 expression score
<10-4
Gal-9 levels in paired colon tissues
● Non-tumor (n=59) ▲ Tumor (n=59)

Supplementary Figure 1. Gal-9 is predominantly expressed in gut Paneth cells and correlated with IBD and colorectal cancer.

(**a**) Quantitative real-time PCR analysis of Gal-9 in the indicated mouse tissues and different segments of the intestine. Gal-9 is barely detected in lymphoid compartments. Duodenum (du), jejunum (je) ileum (il), colon (co), Peyer's Patch (PP), mesenteric lymph node (mLN), inguinal lymph node (iLN), and spleen (SP) samples are indicated. Representative data from two independent and reproducible experiments are shown. (**b**) Immunofluorescence analysis of Gal-9 in ileum and colon tissue sections. In the villus, Gal-9 is mainly expressed in the apical and basolateral sides (left panels), while Gal-9 is enriched at the crypt base of ileum and colon (middle and right panels). (**c**) Immunofluorescence analysis of stem cell marker Lgr5 (with Lgr5-eGFP reporter mice) and Gal-9 (left panels) or Paneth cell marker MMP7 and Gal-9 (right panels) in intestinal tissue sections. (**d**) Immunohistochemistry (IHC) analysis of human tissue array stained with human Gal-9. Tumor: Colorectal cancer, UC: Ulcerative colitis, CD: Crohn's disease. Representative images from each sample group as indicated in (**e**) are shown. (**e**) Expression score for Gal-9 is determined by the H-score system. (**f**) Gal-9 expression in paired (non-tumor vs tumor region in the same patient) colorectal tissue sections is determined by IHC (left panels) and expression score is calculated (right panel). Statistical significance is indicated (**e**: Unpaired two-tailed t-test, **f**: Paired two-tailed t-test). Error bars indicate "means ± SD". Source data are provided as a Source Data file.

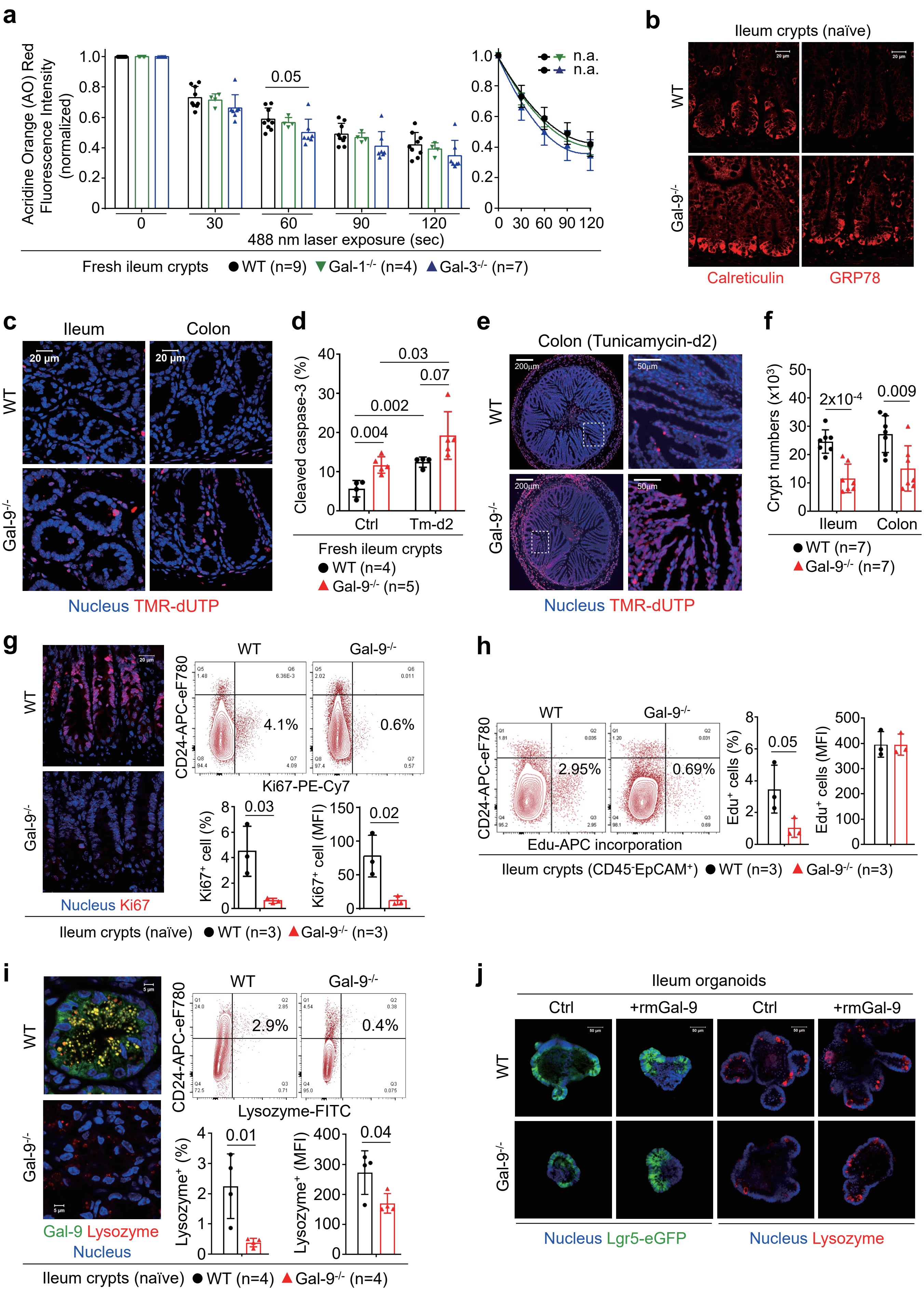
a
Acridine Orange (AO) Red Fluorescence Intensity (normalized)
0.05
488 nm laser exposure (sec)
n.a.
n.a.
Fresh ileum crypts ● WT (n=9) ▼ Gal-1-/- (n=4) ▲ Gal-3-/- (n=7)
b
Ileum crypts (naïve)
WT
Gal-9-/-
Calreticulin
GRP78
c
Ileum
Colon
20 µm
WT
Gal-9-/-
Nucleus TMR-dUTP
d
Cleaved caspase-3 (%)
0.03
0.07
0.002
0.004
Ctrl
Tm-d2
Fresh ileum crypts
● WT (n=4)
▲ Gal-9-/- (n=5)
e
Colon (Tunicamycin-d2)
WT
Gal-9-/-
200µm
50µm
Nucleus TMR-dUTP
f
Crypt numbers (x10³)
2x10-4
0.009
Ileum
Colon
● WT (n=7)
▲ Gal-9-/- (n=7)
g
WT
Gal-9-/-
Nucleus Ki67
CD24-APC-eF780
4.1%
0.6%
Ki67-PE-Cy7
Ki67+ cell (%)
Ki67+ cell (MFI)
0.03
0.02
Ileum crypts (naïve) ● WT (n=3) ▲ Gal-9-/- (n=3)
h
WT
Gal-9-/-
CD24-APC-eF780
2.95%
0.69%
Edu-APC incorporation
Edu+ cells (%)
0.05
Edu+ cells (MFI)
Ileum crypts (CD45-EpCAM+) ● WT (n=3) ▲ Gal-9-/- (n=3)
i
WT
Gal-9-/-
Gal-9 Lysozyme
Nucleus
CD24-APC-eF780
2.9%
0.4%
Lysozyme-FITC
Lysozyme+ (%)
Lysozyme+ (MFI)
0.01
0.04
Ileum crypts (naïve) ● WT (n=4) ▲ Gal-9-/- (n=4)
j
Ileum organoids
Ctrl
+rmGal-9
Ctrl
+rmGal-9
WT
Gal-9-/-
Nucleus Lgr5-eGFP
Nucleus Lysozyme

Supplementary Figure 2. Gal-9 regulates epithelial cell homeostasis in the intestine.

(**a**) Fresh ileum crypts from the indicated mice are labelled with acridine orange (AO) and then exposed to a 488nm laser to induce lysosomal damage. Confocal images after laser exposure are taken at the indicated time points. Loss of lysosome stability is determined by the decrease of AO red fluorescence over time which is normalized to cells exposed at 0 sec. (**b**) Immunofluorescence analysis of ileum tissue sections, stained with ER stress markers, Calreticulin and GRP78. (**c**) Immunofluorescence of the indicated intestinal tissue sections from naïve mice for TUNEL (TMR-dUTP) analysis. (**d**) Flow cytometry analysis of active caspase-3 for cell apoptosis in fresh ileum crypts isolated from naïve mice or mice injected with tunicamycin at day-2 (Tm-d2). (**e**) Immunofluorescence of colon tissue sections from tunicamycin-injected mice at day-2 for TUNEL analysis. (**f**) Freshly isolated crypts from naïve mice are counted under phase contrast microscopy and quantified. (**g**) Immunofluorescence analysis of Ki67 in ileum tissue sections (left panels) and flow cytometry analysis of $CD24^{low}$ $Ki67^{+}$ proliferating cells in fresh ileum crypts (right panels) from naïve mice. (**h**) Flow cytometry analysis of fresh ileum crypts isolated from mice 3 hr post Edu-dye injection for Edu incorporation to assess crypt proliferation within 3 hr. (**i**) Immunofluorescence analysis of Paneth cell marker lysozyme in ileum tissue sections (left panels) and flow cytometry analysis of $CD24^{high}$ $Lysozyme^{+}$ Paneth cells in fresh ileum crypts from naïve mice (right panels). (**j**) Confocal microscopy analysis of the stem cell marker Lgr5 (by Lgr5-eGFP reporter mice) or Paneth cell marker lysozyme in ileum organoids, cultured with or without recombinant mouse Gal-9 (rmGal-9). Results shown are representative (**d**,**g**,**h**,**i**) or combined (**a**,**f**) data from two independent and reproducible experiments. Statistical significance is indicated (**a**,**d**,**f**,**g**,**h**,**i**: Unpaired two-tailed t-test). Error bars indicate "means ± SD". Source data are provided as a Source Data file.

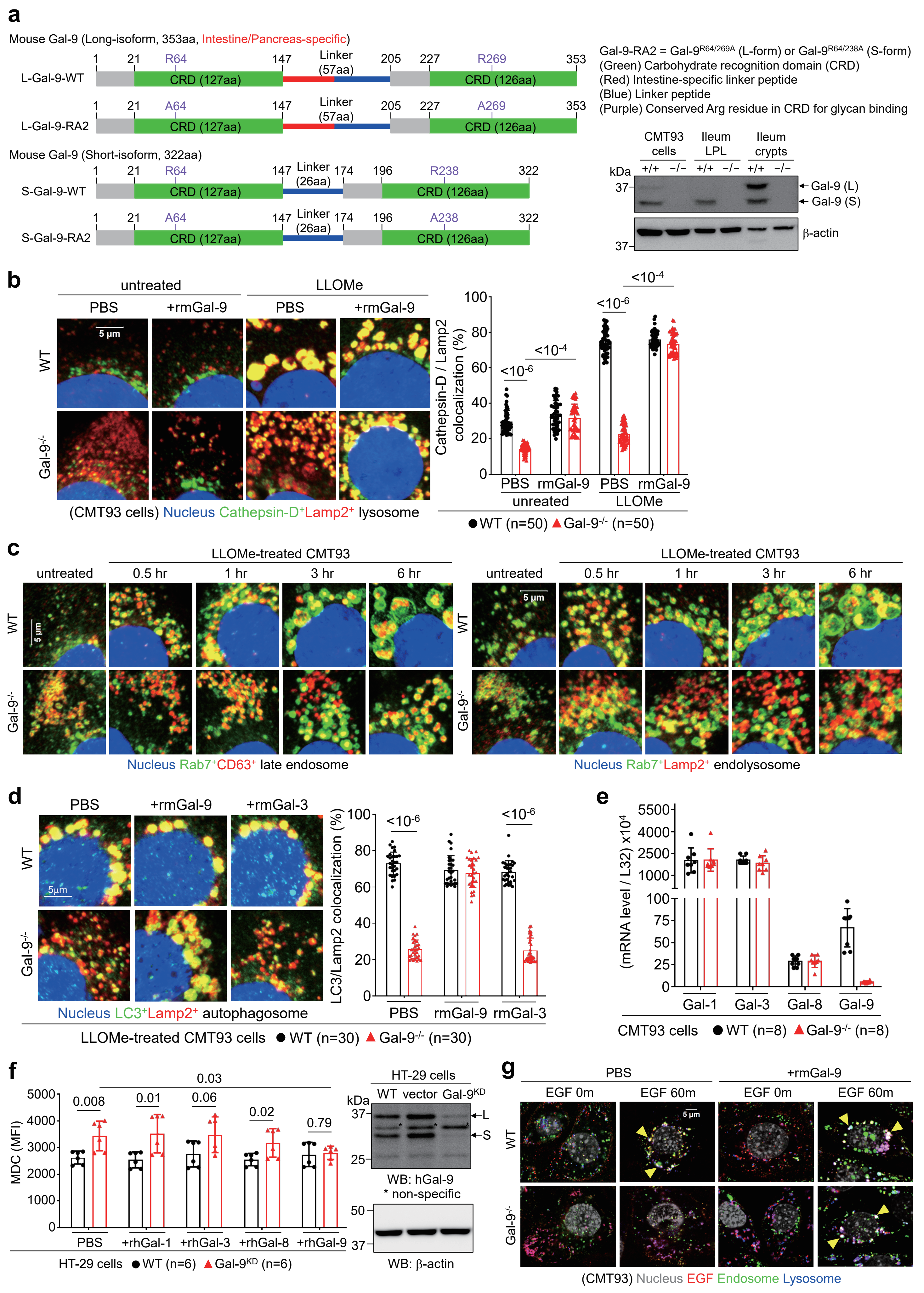
a
Mouse Gal-9 (Long-isoform, 353aa, Intestine/Pancreas-specific)
L-Gal-9-WT
L-Gal-9-RA2
CRD (127aa)
CRD (126aa)
Linker (57aa)
Mouse Gal-9 (Short-isoform, 322aa)
S-Gal-9-WT
S-Gal-9-RA2
Linker (26aa)
Gal-9-RA2 = Gal-9R64/269A (L-form) or Gal-9R64/238A (S-form)
(Green) Carbohydrate recognition domain (CRD)
(Red) Intestine-specific linker peptide
(Blue) Linker peptide
(Purple) Conserved Arg residue in CRD for glycan binding
CMT93 cells
Ileum LPL
Ileum crypts
kDa
Gal-9 (L)
Gal-9 (S)
β-actin
b
untreated
LLOMe
PBS
+rmGal-9
WT
Gal-9-/-
5 μm
Cathepsin-D / Lamp2 colocalization (%)
(CMT93 cells) Nucleus Cathepsin-D+Lamp2+ lysosome
WT (n=50) Gal-9-/- (n=50)
c
LLOMe-treated CMT93
untreated
0.5 hr
1 hr
3 hr
6 hr
Nucleus Rab7+CD63+ late endosome
Nucleus Rab7+Lamp2+ endolysosome
d
PBS
+rmGal-9
+rmGal-3
LC3/Lamp2 colocalization (%)
Nucleus LC3+Lamp2+ autophagosome
LLOMe-treated CMT93 cells WT (n=30) Gal-9-/- (n=30)
e
(mRNA level / L32) x10^4
Gal-1
Gal-3
Gal-8
Gal-9
CMT93 cells WT (n=8) Gal-9-/- (n=8)
f
MDC (MFI)
PBS
+rhGal-1
+rhGal-3
+rhGal-8
+rhGal-9
HT-29 cells WT (n=6) Gal-9KD (n=6)
HT-29 cells
WT vector Gal-9KD
WB: hGal-9
* non-specific
WB: β-actin
g
EGF 0m
EGF 60m
(CMT93) Nucleus EGF Endosome Lysosome

Supplementary Figure 3. Gal-9 regulates perinuclear puncta formation during endolysosomal /lysosomal damage and promotes homeostatic cargo degradation at the steady state.

(**a**) Schematic illustration of mouse long (L) and short (S) isoforms of Gal-9 (left panel). Wild-type or N-glycan-binding deficient mutant of Gal-9 with double substitutions of $Arg(R)^{64}$ and $Arg(R)^{269}$ in L-form (or $Arg(R)^{238}$ in S-form) with Ala (A) in each of the CRD (abbreviated as RA2) is indicated. Western blot analysis of Gal-9 in CMT93 cells, fresh ileum lamina propria lymphocyte (LPL) sample, and fresh ileum crypts from mouse (right panel). The long isoform of Gal-9 is not detected in LPL sample. CRD: carbohydrate recognition domain. (**b**) Immunofluorescence analysis of perinuclear puncta containing Cathepsin-D^+ $Lamp2^+$ lysosomes (shown in yellow) in the indicated CMT93 cells, cultured with or without recombinant mouse Gal-9 (rmGal-9), and treated with LLOMe for 3 hr to induce endolysosomal/lysosomal damage and subsequent formation of perinuclear puncta. Colocalization of Cathepsin-D and Lamp2 is quantified and shown as percentage. (**c**) Immunofluorescence and time-course (0-6 hr) analysis of perinuclear puncta containing $Rab7^+CD63^+$ late endosomes or $Rab7^+Lamp2^+$ endolysosomes (both shown in yellow) in the indicated CMT93 cells, untreated or treated with LLOMe for 3 hr to induce endolysosomal/lysosomal damage and subsequent formation of perinuclear puncta. (**d**) Immunofluorescence analysis of perinuclear puncta containing $LC3^+Lamp2^+$ autophagosomes (shown in yellow) in the indicated CMT93 cells, cultured with or without rmGal-9, or rmGal-3, and treated with LLOMe for 3 hr to induce endolysosomal/lysosomal damage and subsequent perinuclear puncta. Colocalization of LC3 and Lamp2 is quantified and shown as percentage. (**e**) Quantitative real-time PCR analysis of the indicated endogenous galectins in CMT93 cells. (**f**) Flow cytometry analysis of MDC^+ autophagic vacuoles in the indicated HT-29 cells, cultured with recombinant human Gal-1 (rhGal-1), rhGal-3, rhGal8, or rhGal-9, and stained with MDC (left panel). Western blot analysis of Gal-9 in Gal-9 gene-knockdown (KD) HT-29 cells (Gal-9^{KD}, right panel). *non-specific. (**g**) Immunofluorescence analysis of the indicated CMT93 cells, cultured with or without rmGal-9, for degradation of endocytosed fluorochrome-labeled EGF (shown in red). Cells are first pulsed with Alexa Flour 555-conjugated EGF for 5 min. To visualize endosomes and lysosomes, cells are then labeled with pHrodo™ Green Dextran and LysoTracker™ Deep Red, respectively, and are live imaged at 0 h before chase and 1 h after chase. White pseudo-color puncta (indicated by arrowheads) represent EGF^+ endolysosomes moving to the perinuclear region for fusion and degradation. Results shown are representative (**a**,**b**,**c**,**d**,**g**) or combined (**e**,**f**) data from two independent and reproducible experiments. Statistical significance is indicated (**b**,**d**,**e**,**f**: Unpaired two-tailed t-test). Error bars indicate "means ± SD". Source data are provided as a Source Data file.

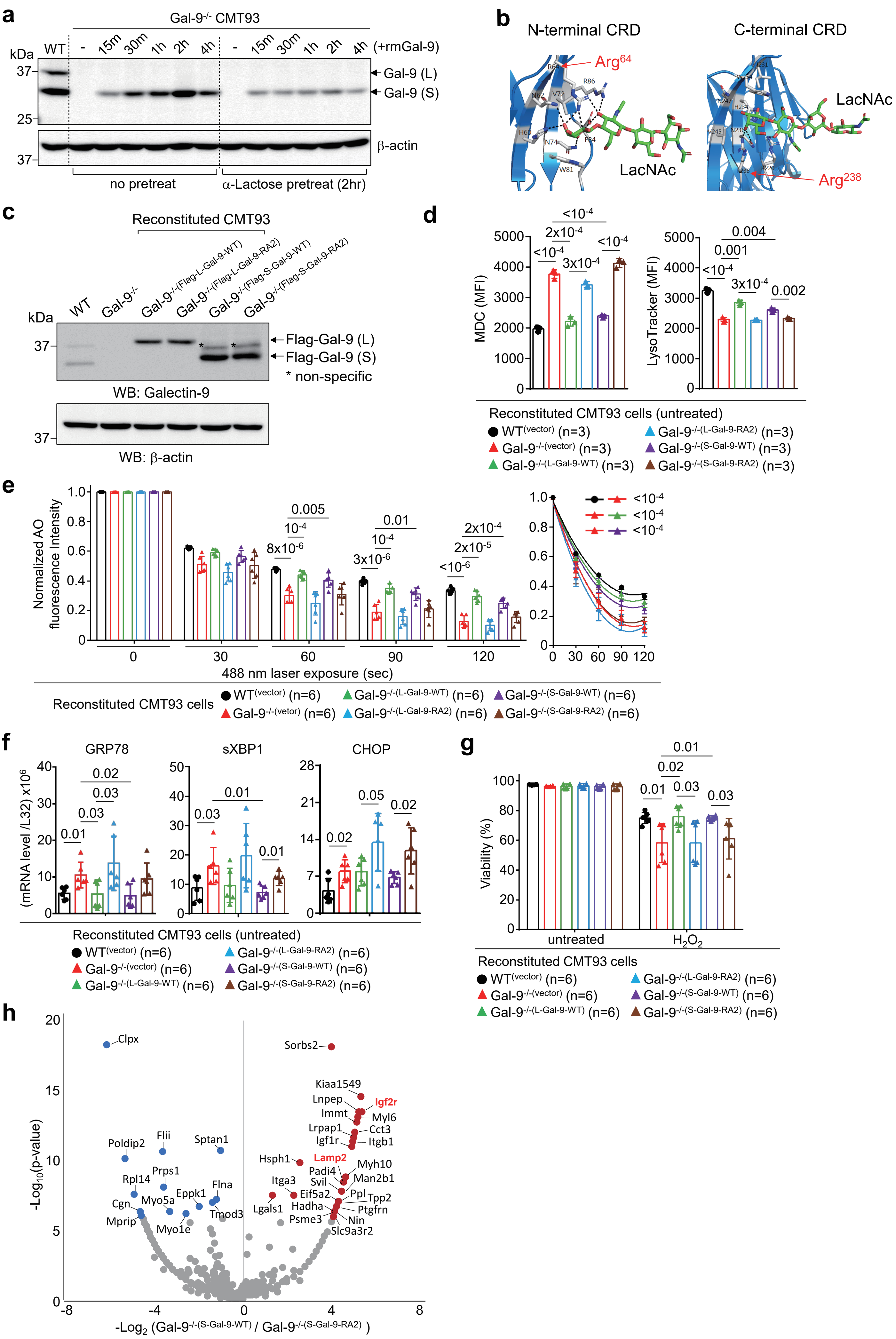
a
Gal-9-/- CMT93
kDa
WT
- 15m 30m 1h 2h 4h - 15m 30m 1h 2h 4h (+rmGal-9)
37
25
37
Gal-9 (L)
Gal-9 (S)
β-actin
no pretreat
α-Lactose pretreat (2hr)
b
N-terminal CRD
C-terminal CRD
Arg64
LacNAc
LacNAc
Arg238
c
Reconstituted CMT93
WT
Gal-9-/-
Gal-9-/-(Flag-L-Gal-9-WT)
Gal-9-/-(Flag-L-Gal-9-RA2)
Gal-9-/-(Flag-S-Gal-9-WT)
Gal-9-/-(Flag-S-Gal-9-RA2)
kDa
37
Flag-Gal-9 (L)
Flag-Gal-9 (S)
* non-specific
WB: Galectin-9
37
WB: β-actin
d
MDC (MFI)
LysoTracker (MFI)
Reconstituted CMT93 cells (untreated)
WT(vector) (n=3)
Gal-9-/-(vector) (n=3)
Gal-9-/-(L-Gal-9-WT) (n=3)
Gal-9-/-(L-Gal-9-RA2) (n=3)
Gal-9-/-(S-Gal-9-WT) (n=3)
Gal-9-/-(S-Gal-9-RA2) (n=3)
e
Normalized AO fluorescence Intensity
488 nm laser exposure (sec)
Reconstituted CMT93 cells
WT(vector) (n=6)
Gal-9-/-(vetor) (n=6)
Gal-9-/-(L-Gal-9-WT) (n=6)
Gal-9-/-(L-Gal-9-RA2) (n=6)
Gal-9-/-(S-Gal-9-WT) (n=6)
Gal-9-/-(S-Gal-9-RA2) (n=6)
f
GRP78
sXBP1
CHOP
(mRNA level /L32) x10⁶
Reconstituted CMT93 cells (untreated)
WT(vector) (n=6)
Gal-9-/-(vector) (n=6)
Gal-9-/-(L-Gal-9-WT) (n=6)
Gal-9-/-(L-Gal-9-RA2) (n=6)
Gal-9-/-(S-Gal-9-WT) (n=6)
Gal-9-/-(S-Gal-9-RA2) (n=6)
g
Viability (%)
untreated
H2O2
Reconstituted CMT93 cells
WT(vector) (n=6)
Gal-9-/-(vector) (n=6)
Gal-9-/-(L-Gal-9-WT) (n=6)
Gal-9-/-(L-Gal-9-RA2) (n=6)
Gal-9-/-(S-Gal-9-WT) (n=6)
Gal-9-/-(S-Gal-9-RA2) (n=6)
h
-Log10(p-value)
-Log2 (Gal-9-/-(S-Gal-9-WT) / Gal-9-/-(S-Gal-9-RA2))
Clpx
Sorbs2
Kiaa1549
Lnpep
Igf2r
Immt
Myl6
Lrpap1
Cct3
Igf1r
Itgb1
Poldip2
Flii
Sptan1
Hsph1
Lamp2
Myh10
Prps1
Padi4
Man2b1
Rpl14
Flna
Itga3
Svil
Eppk1
Eif5a2
Ppl
Tpp2
Cgn
Myo5a
Lgals1
Hadha
Ptgfrn
Tmod3
Psme3
Nin
Mprip
Myo1e
Slc9a3r2

Supplementary Figure 4. Arg^{64} and Arg^{269} (or Arg^{238} in S-form) in the CRD of Gal-9 mediates N-glycan binding and regulates lysosome-mediated autophagy, ER stress, and stress-associated cell viability.

(**a**) Western blot analysis of endocytosis of exogenous recombinant mouse Gal-9 (rmGal-9, S-form) in rmGal-9-treated wild-type and Gal-9$^{-/-}$ CMT93 cells, with or without prior α-lactose pre-treatment for 2 hr. (**b**) Illustration of the carbohydrate recognition domain (CRD) structures in mouse S-form Gal-9, represented by the ribbon diagrams of N-terminal (left panel) or C-terminal (right panel) CRDs binding to N-acetyl-lactosamine (LacNAc) (shown in grey and green sticks) through local hydrogen bonding (dotted black lines). The critical residues, Arg(R)^{64} and Arg(R)^{238}, important for hydrogen bonding, are shown in red. (**c**) Western blot analysis of Gal-9 expression in the indicated reconstituted CMT-93 cells. Gal-9$^{-/-(L\text{-}Gal\text{-}9\text{-}WT)}$ and Gal-9$^{-/-(S\text{-}Gal\text{-}9\text{-}WT)}$ indicate Gal-9$^{-/-}$ CMT93 cells stably reconstituted with L-form or S-form of wild-type Gal-9, respectively, while Gal-9$^{-/-(L\text{-}Gal\text{-}9\text{-}RA2)}$ and Gal-9$^{-/-(S\text{-}Gal\text{-}9\text{-}RA2)}$ indicate Gal-9$^{-/-}$ CMT93 cells reconstituted with mutant L-form (Arg$^{64/269}$ to Ala$^{64/269}$ or RA2) or S-form (Arg$^{64/238}$ to Ala$^{64/238}$) of glycan-binding-deficient Gal-9, respectively. *non-specific. (**d**) Flow cytometry analysis of autophagic vacuoles (stained with MDC) or acidic compartments (stained with LysoTracker) in the indicated reconstituted CMT93 cells at the steady state. (**e**) The indicated reconstituted CMT93 cells are labelled with AO and then exposed to a 488nm laser to induce lysosomal damage. Confocal images after laser exposure are taken at the indicated time points. Loss of lysosome stability is determined by the decrease of AO red fluorescence over time which is normalized to cells exposed at 0 sec. (**f**) Quantitative real-time PCR analysis of ER stress-associated genes in the indicated CMT93 cells at the steady state. (**g**) Flow cytometry analysis of the indicated CMT93 cells, untreated or treated with H_2O_2 to induce oxidative stress-associated cell death, and stained with fluorescent viability dye to determine cell viability. (**h**) Volcano plot analysis of Gal-9 binding proteins in CMT93 cells, identified by mass spectrometry. Dfferential Gal-9-binding proteins between glycan-binding sufficient (S form of Gal-9 WT) and deficient (S form of Gal-9-RA2) cells with adjusted p-value significance are shown in blue dots (stronger binding by Gal-9-WT) or in red dots (stronger binding by Gal-9-RA2). Results shown are representative (**a**,**c**,**d**,**h**) or combined data (**e**,**f**,**g**) from two independent and reproducible experiments. Statistical significance is indicated (**d**,**e**,**f**,**g**: Unpaired two-tailed t-test, **h**: The p-values are calculated and obtained from two-sided Fisher's exact test using Benjamini and Hochberg method for multiple testing correction). Error bars indicate "means ± SD". Source data are provided as a Source Data file.

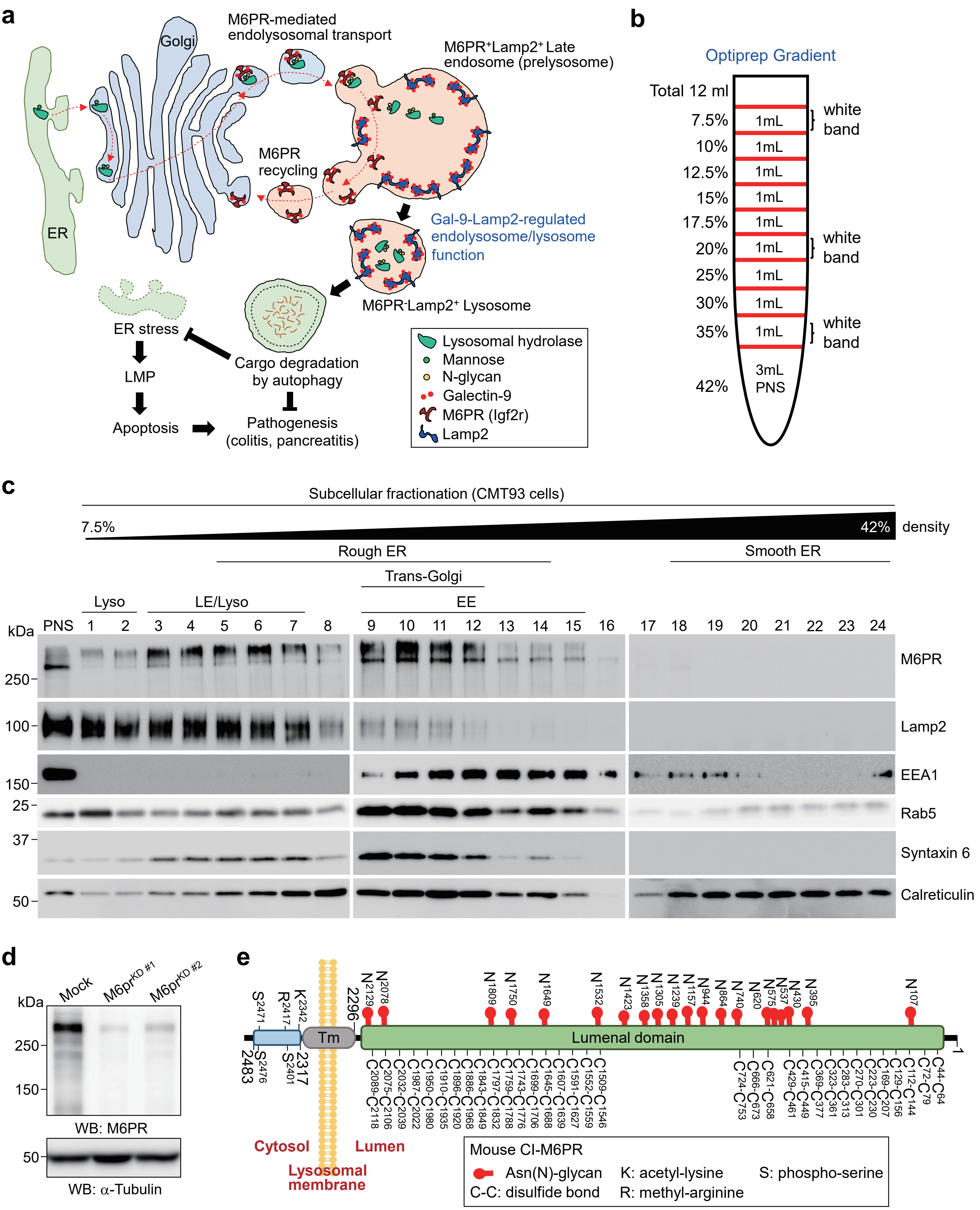

a
Golgi
M6PR-mediated endolysosomal transport
M6PR⁺Lamp2⁺ Late endosome (prelysosome)
M6PR recycling
ER
Gal-9-Lamp2-regulated endolysosome/lysosome function
M6PR⁻Lamp2⁺ Lysosome
ER stress
LMP
Apoptosis
Cargo degradation by autophagy
Pathogenesis (colitis, pancreatitis)
Lysosomal hydrolase
Mannose
N-glycan
Galectin-9
M6PR (Igf2r)
Lamp2
b
Optiprep Gradient
Total 12 ml
7.5%
10%
12.5%
15%
17.5%
20%
25%
30%
35%
42%
1mL
3mL PNS
white band
c
Subcellular fractionation (CMT93 cells)
7.5%
42%
density
Rough ER
Smooth ER
Trans-Golgi
Lyso
LE/Lyso
EE
kDa
PNS 1 2 3 4 5 6 7 8 9 10 11 12 13 14 15 16 17 18 19 20 21 22 23 24
250
100
150
25
37
50
M6PR
Lamp2
EEA1
Rab5
Syntaxin 6
Calreticulin
d
kDa
Mock
M6pr KD #1
M6pr KD #2
250
150
50
WB: M6PR
WB: α-Tubulin
e
S2471
R2417
K2342
2296
2483
S2476
S2401
2317
Tm
Lumenal domain
1
N2129 N2078 N1809 N1750 N1649 N1532 N1423 N1358 N1305 N1239 N1157 N944 N864 N740 N620 N575 N537 N430 N395 N107
C2089-C2118 C2075-C2106 C2032-C2039 C1987-C2022 C1950-C1980 C1910-C1935 C1896-C1920 C1886-C1968 C1843-C1849 C1797-C1832 C1759-C1788 C1743-C1776 C1699-C1706 C1645-C1688 C1607-C1639 C1591-C1627 C1552-C1559 C1509-C1546
C724-C753 C666-C673 C621-C658 C429-C461 C415-C449 C369-C377 C323-C361 C283-C313 C270-C301 C223-C230 C169-C207 C129-C156 C112-C144 C72-C79 C44-C64
Cytosol
Lumen
Lysosomal membrane
Mouse CI-M6PR
Asn(N)-glycan
K: acetyl-lysine
S: phospho-serine
C-C: disulfide bond
R: methyl-arginine

Supplementary Figure 5. M6PR is more enriched in early endosomes.

(**a**) Graphic illustration showing that lumenal Gal-9 interacts with Lamp2 to promote endolysosome/lysosome function and facilitate cargo degradation by autophagy, which alleviates ER stress and prevents LMP and stress-associated cell death. M6PR-mediated endolysosomal transport and M6PR recycling between the trans-Golgi network and endolysosomal compartments are indicated. (**b**) Preparation of density step-gradient for subcellular fractionation. (**c**) Western blot analysis of subcellular fractionations of CMT93 cells by density gradient ultra-centrifugation. PNS: post nuclear supernatant, LE/Lyso: late endosome/lysosome fractions. EE: early endosome fractions. Lamp2 is a marker for LE/Lyso. EEA1 and Rab5 are markers for EE. Syntaxin 6 is a marker for Trans-Golgi. Calreticulin is a marker for ER. (**d**) Western blot analysis of M6PR in gene-knockdown (KD) M6pr^{KD} CMT93 cells. (**e**) Schematic illustration of mouse (NP_034645.2) cation-independent mannose-6-phosphate receptor (CI-M6PR or Igf2r). The potential residues for Asn(N)-glycosylation and for disulfide bond formation in the lumenal domain of M6PR are indicated. Results shown are representative (**c**,**d**) from two independent and reproducible experiments.

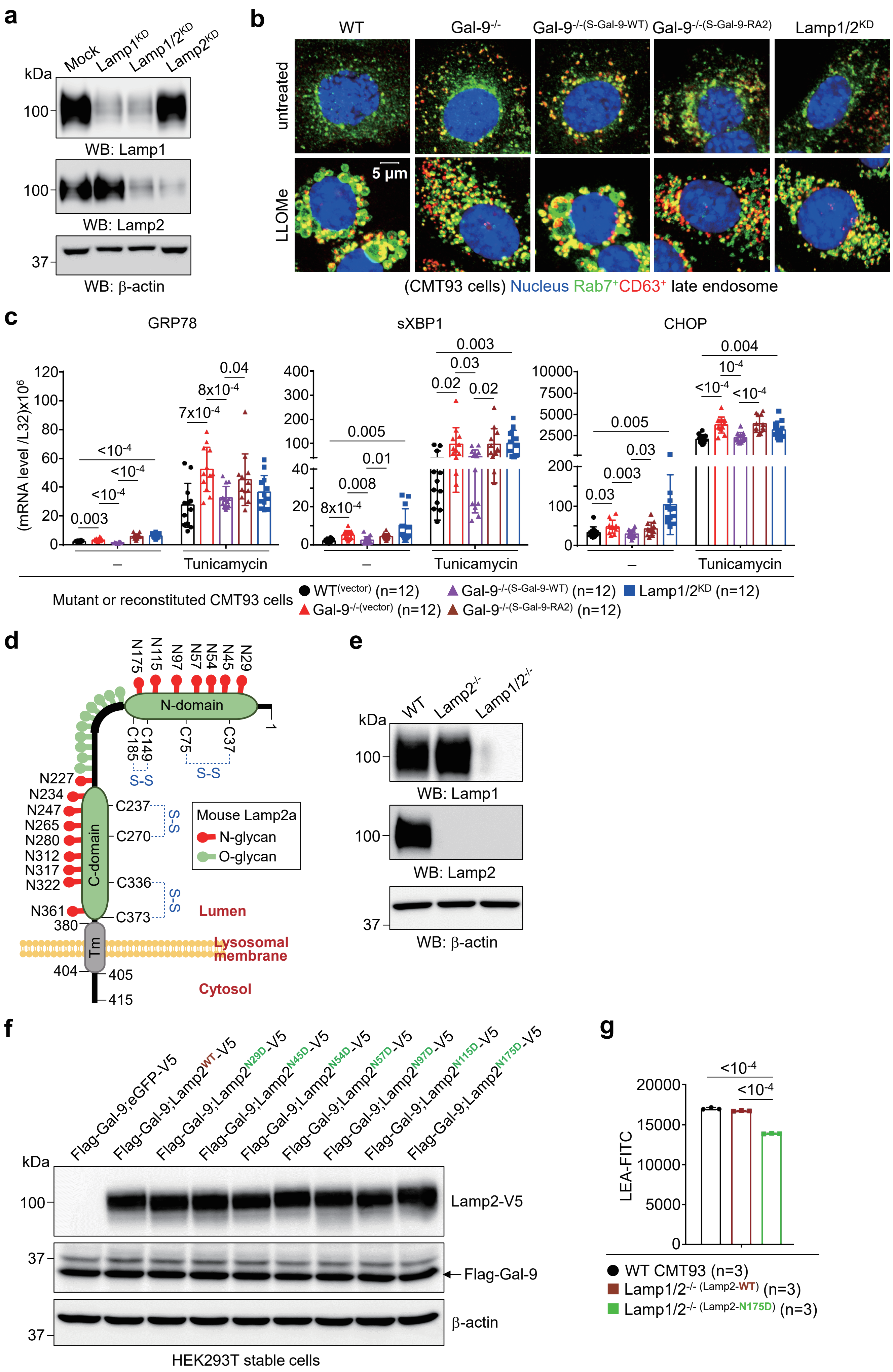
a
Mock
Lamp1KD
Lamp1/2KD
Lamp2KD
kDa
100
WB: Lamp1
100
WB: Lamp2
37
WB: β-actin
b
WT
Gal-9-/-
Gal-9-/-(S-Gal-9-WT)
Gal-9-/-(S-Gal-9-RA2)
Lamp1/2KD
untreated
LLOMe
5 μm
(CMT93 cells) Nucleus Rab7+CD63+ late endosome
c
GRP78
sXBP1
CHOP
(mRNA level /L32)x10^6
Tunicamycin
Mutant or reconstituted CMT93 cells
WT(vector) (n=12)
Gal-9-/-(vector) (n=12)
Gal-9-/-(S-Gal-9-WT) (n=12)
Gal-9-/-(S-Gal-9-RA2) (n=12)
Lamp1/2KD (n=12)
d
N-domain
C-domain
Tm
Mouse Lamp2a
N-glycan
O-glycan
S-S
Lumen
Lysosomal membrane
Cytosol
e
WT
Lamp2-/-
Lamp1/2-/-
kDa
100
WB: Lamp1
100
WB: Lamp2
37
WB: β-actin
f
Flag-Gal-9;eGFP-V5
Flag-Gal-9;Lamp2WT-V5
Flag-Gal-9;Lamp2N29D-V5
Flag-Gal-9;Lamp2N45D-V5
Flag-Gal-9;Lamp2N54D-V5
Flag-Gal-9;Lamp2N57D-V5
Flag-Gal-9;Lamp2N97D-V5
Flag-Gal-9;Lamp2N115D-V5
Flag-Gal-9;Lamp2N175D-V5
kDa
100
Lamp2-V5
37
Flag-Gal-9
β-actin
37
HEK293T stable cells
g
LEA-FITC
<10-4
<10-4
WT CMT93 (n=3)
Lamp1/2-/- (Lamp2-WT) (n=3)
Lamp1/2-/- (Lamp2-N175D) (n=3)

Supplementary Figure 6. Binding of Gal-9 to glycosylated Asn^{175} in Lamp2 is involved in lysosome function.

(**a**) Western blot analysis of the indicated CMT93 cells to determine the Lamp1/2 gene-knockdown (KD) by siRNA against Lamp1, Lamp2, or both. (**b**) Immunofluorescence analysis of perinuclear puncta containing $Rab7^+CD63^+$ late endosomes (shown in yellow) in the indicated CMT93 cells, untreated or treated with LLOMe for 3 hr to induce endolysosomal/lysosomal damage and subsequent perinuclear puncta formation. (**c**) Quantitative real-time PCR analysis of ER stress-associated genes in the indicated CMT93 cells, untreated or treated with tunicamycin to induce ER stress. (**d**) Schematic illustration of mouse (NP_001017959) Lamp2a. The potential residues for N (Asn)-linked and O (Ser/Thr)-linked glycosylations are shown in red and green sticks, respectively. S-S: disulphide bonds; Tm: transmembrane domain. (**e**) Western blot analysis of the indicated CMT93 cells to determine the Lamp1/2 knockout (KO) efficiency by CRISPR/Cas9-targeted Lamp2, or Lamp1/Lamp2. (**f**) Western blot analysis of Lamp2-V5 (by anti-V5 antibody) and Gal-9 (by anti-Flag antibody) expression in the indicated transfected HEK293T cells. (**g**) Flow cytometry analysis of tomato lectin (LEA) binding to LacNAc in the indicated CMT-93 cells. Results shown are representative (**a**,**b**,**e**,**f**,**g**) or combined (**c**) data from two independent and reproducible experiments. Statistical significance is indicated (**c**,**g**: Unpaired two-tailed t-test). Error bars indicate "means ± SD". Source data are provided as a Source Data file.

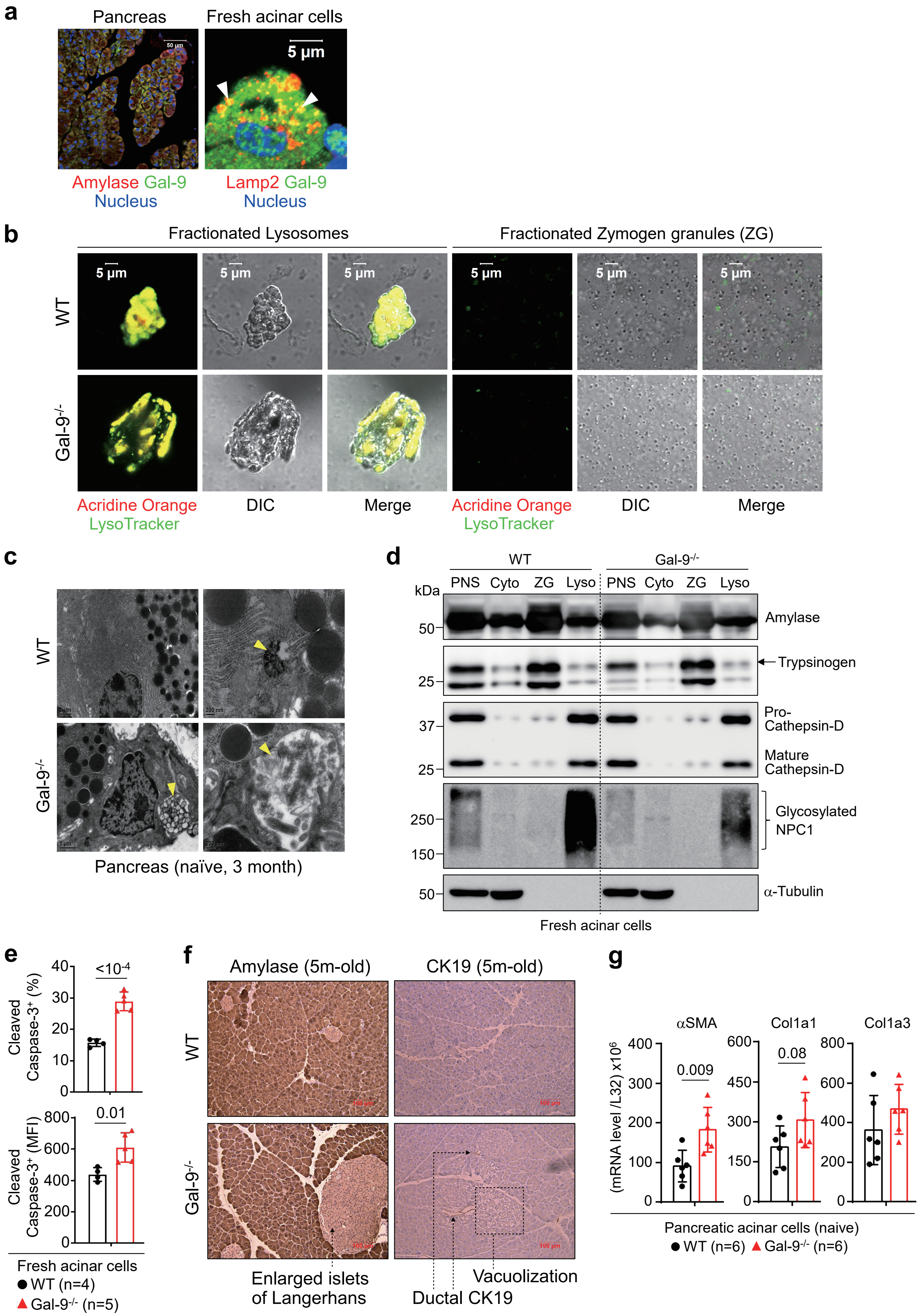
a
Pancreas
Fresh acinar cells
50 µm
5 µm
Amylase Gal-9
Nucleus
Lamp2 Gal-9
Nucleus
b
Fractionated Lysosomes
Fractionated Zymogen granules (ZG)
WT
Gal-9-/-
5 µm
Acridine Orange
LysoTracker
DIC
Merge
c
WT
Gal-9-/-
Pancreas (naïve, 3 month)
d
WT
Gal-9-/-
kDa
PNS
Cyto
ZG
Lyso
Amylase
Trypsinogen
Pro-Cathepsin-D
Mature Cathepsin-D
Glycosylated NPC1
α-Tubulin
50
25
37
250
150
Fresh acinar cells
e
Cleaved Caspase-3+ (%)
<10-4
Cleaved Caspase-3+ (MFI)
0.01
Fresh acinar cells
● WT (n=4)
▲ Gal-9-/- (n=5)
f
Amylase (5m-old)
CK19 (5m-old)
WT
Gal-9-/-
Enlarged islets of Langerhans
Vacuolization
Ductal CK19
g
αSMA
Col1a1
Col1a3
(mRNA level /L32) x10^6
0.009
0.08
Pancreatic acinar cells (naive)
● WT (n=6) ▲ Gal-9-/- (n=6)

Supplementary Figure 7. Gal-9 regulates lysosome function and ER stress in acinar cells to prevent pancreatic disorders.

(**a**) Immunofluorescence analysis of Gal-9 and amylase in pancreatic tissue sections from naïve mice (left panel) and Gal-9 and Lamp2 in fresh acinar cells (right panel). Arrowheads indicate Gal-9 colocalization with lysosome marker Lamp2 (shown in yellow puncta). (**b**) Immunofluorescence analysis of acidic compartments by acridine orange and LysoTracker staining in fractionated lysosomes and zymogen granules (ZGs), isolated from fresh acinar cells. (**c**) Transmission electron microscopy analysis of pancreas. Wild-type mouse showed a normal degradative autophagic vacuole (upper right panel), while Gal-9$^{-/-}$ mouse showed a large aberrant autolysosome containing partially digested materials or concentric multi-lamellar membrane-containing vacuole (lower panels) indicative of autophagy blockade. (**d**) Western blot analysis of the indicated lysosomal or ZG proteins in subcellular fractionations of fresh acinar cells. PNS: post nuclear supernatant, Cyto: cytosol fraction, ZG: zymogen granule fraction, Lyso: lysosome fraction. (**e**) Flow cytometry analysis of apoptotic cells by cleaved Caspase-3 in fresh acinar cells from the indicated naïve mice. (**f**) Immunochemistry analysis of pancreatic tissue sections for metaplasia and fibrosis by staining with amylase and CK19. Clinical manifestations of chronic pancreatitis are indicated. (**g**) Quantitative real-time PCR analysis of fibrosis-associated genes in freshly isolated acinar cells. Results shown are representative (**a**,**b**,**c**,**d**,**f**) or combined (**e**,**g**) data from two independent and reproducible experiments. Statistical significance is indicated (**e**,**g**: Unpaired two-tailed t-test). Error bars indicate "means ± SD". Source data are provided as a Source Data file.

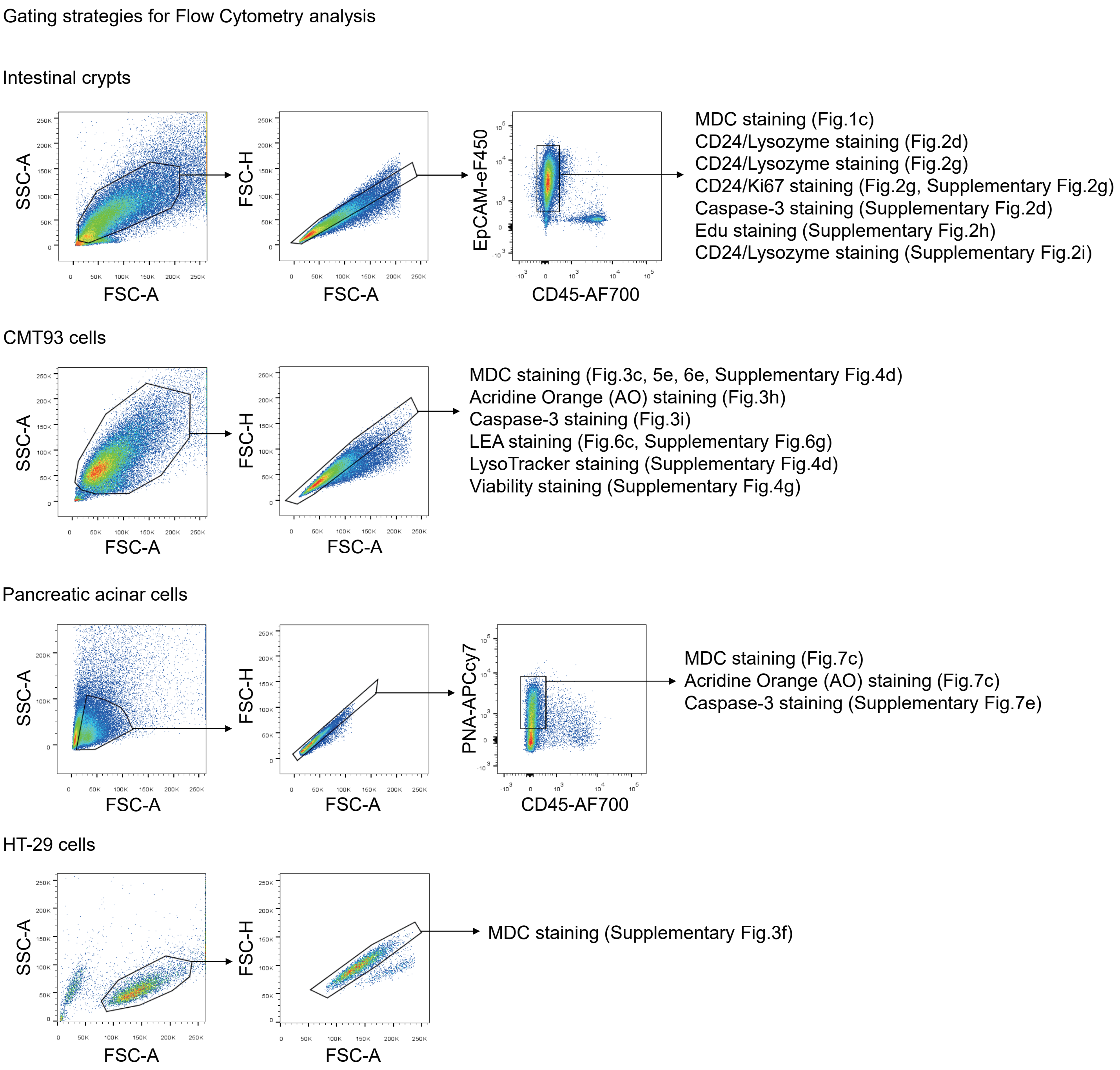

Supplementary Figure 8. Gating strategies for Flow Cytometry analysis.

Gating strategies used for intestinal crypts, CMT93 cells, pancreatic acinar cells, and HT-29 cells are indicated.

Supplementary Table 1. List of primers used for Quantitative real-time PCR.

Gene	Forward primer (5' to 3')	Reverse primer (5' to 3')
CHOP	CCACCACACCTGAAAGCAGAA	AGGTGAAAGGCAGGGACTCA
Col1a1	GAGAGGTGAACAAGGTCCCG	AAACCTCTCTCGCCTCTTGC
Col1a3	CAGGAGAAAAGGGTCCTCCC	ATACCCCGTATCCCTGGACC
Cryptdin	GAGGAATCGTTGAGAGATCTGGTATG	CCATGTTCAGCGACAGCAGAGC
Defensin-β3	GCTAGGGAGCACTTGTTTGC	TTGTTTGAGGAAAGGAGGCA
Galectin-1	CTCTCGGGTGGAGTCTTCTG	GGTTTGAGATTCAGGTTGCTG
Galectin-3	GCCTACCCCAGTGCTCCT	GGTCATAGGGCACCGTCA
Galectin-8	ATCGTGTTCATGGTGCTCAA	CGTACAGCAGAACATGCCTTC
Galectin-9	ACCCTACCACCTCGTGGA	GACAGGGGCTGCAGAGTTC
GRP78	ACTTGGGGACCACCTATTCCT	ATCGCCAATCAGACGCTCC
L32	GAAACTGGCGGAAACCCA	GGATCTGGCCCTTGAACCTT
α-SMA	GTCCACCGCAAATGCTTCTA	GAGGCGCTGATCCACAAAAC
sXBP1	CTGAGTCCGAATCAGGTGCAG	GTCCATGGGAAGATGTTCTGG

Supplementary Methods

Cell culture

Mouse epithelial, adherent rectal polyploid carcinoma cell line CMT93 (ATCC CCL-223), Human epithelial, adherent colorectal adenocarcinoma cell line HT-29 (ATCC® HTB-38™), and Human epithelial, adherent embryonic kidney cell line HEK293 (ATCC® CRL-1573™) were purchased from American Type Culture Collection (ATCC), were used for the *in vitro* studies. The cells were maintained as a monolayer in ATCC-formulated Dulbecco's Modified Eagle's Medium (DMEM) (Gibco #30-2002) supplemented with 10% fetal bovine serum (FBS) (Gibco #10437-028), 100 U/ml of penicillin and 100 μg/ml of streptomycin (Gibco #15140-122), 50 μg/ml of Gentamicin (Gibco #15710-064) and 2 mM GlutaMAX (Gibco #35050-061) in a 37 ºC humidified 5% CO_2 incubator. For passage, 80-90% confluent cells were washed with phosphate buffered saline (PBS) and trypsinised with 5*x* or 1*x* Trypsin-EDTA (0.5%) (Gibco #15400054) for 5 min. The cells were split at 1:10 subcultivation ratio for regular maintenance and the complete medium was renewed every 2 to 3 times/week. The cells were counted by using the Nexcelom, Cellometer Auto T4 Plus automated cell counting system. To explore the lysosomal regulation mediated by galectins, CMT93 cells were treated with or without 0.5 μg/ml recombinant mouse galectin-9 protein (R&D #3535-GA) or galectin-3 protein (R&D #1197-GA) for 48 h, and HT-29 cells were treated with or without 0.5 μg/ml recombinant human galectin-1 (R&D #1152-GA), galectin-3 (R&D #1154-GA), galectin-8, galectin-9 (R&D #2045-GA) for 48 h, respectively. The recombinant human Gal-8 was a gift from Dr. Kuo-I Lin, Genomics Research Center, Academia Sinica, Taipei, Taiwan.

Quantification of lysosomal acidic compartments by AO

Acridine Orange (AO) is cell permeable and in an acidic environment becomes protonated, sequestered and emits red fluorescence with an intensity proportional to the degree of acidity or to the acidic compartment volume. To visualize and measure the lysosomal/autolysosomal acidic compartments and assess their stability, function, and membrane integrity in cells, pH-sensitive fluorogenic organic weak base Acridine Orange dye (Sigma #A9231) was used in both confocal microscopy and flow cytometry. For confocal microscopy, ileal crypts were freshly isolated as previously described and about ~1000 crypts were incubated with AO (2 μg/ml) in ENR growth media at 37ºC humidified 5% CO2 incubator for 10 min. The crypts were washed in PBS and then visualized for red fluorescence under Carl Zeiss LSM 700 stage confocal microscope using 20*x* objective. For quantitative analysis, the isolated crypts were dissociated into single cells as previously described. The cells were incubated with AO (0.4 μg/ml) or Lysotracker DND-26 (15 nM) in flow staining buffer at 37 ºC for 15 min, later washed with PBS and stained with surface antibody markers if mentioned for 15 min at 4°C. The cells were analysed by flow cytometry with the excitation wavelength set of 488 nm and readings made at 515~545 nm and 685~735 nm.

Autophagy flux assay

To measure autophagic flux CMT93 cells (0.25×10^6 cells) were seeded into 6-cm dish and grown until confluency, whereas the freshly isolated acinar cells from whole pancreas were split into 4 parts and placed in 6-well plate containing 3 ml Waymouth's medium. The cells were treated with 500 nM mTOR inhibitor Rapamycin (InvivoGen #tlrl-rap), or 50 nM V-

ATPase inhibitor Bafilomycin A1 (InvivoGen #tlrl-baf1), or both. The untreated well was used a negative control, 16 hours later the cells were collected and lysed in 1% NP-40 lysis buffer containing 1*x* Protease Inhibitor Cocktail (MedChemExpress #HY-K0010). The samples were western blotted and probed with LC3B antibody, autophagic flux was analysed by comparing the LC3-II bands between wild-type and Galectin-9 knockout cells.

Endocytosis of exogenous galectin-9

To determine the internalization of recombinant mouse galectin-9 protein (rmGal-9), Galectin-9 knockout CMT93 cells (0.25 x 10^6 cells) were seeded into 6-cm dish and grown until confluency. The cells were washed with PBS and treated with complete media containing with or without 50 mM α-Lactose monohydrate (Sigma-Aldrich #L2643) for 2 h, and then 0.5 μg/ml rmGal-9 (R&D systems #3535-GA) was added to untreated or treated cells without media change and incubated for 15, 30, 60, 120, and 240 min, respectively. The cells which did not receive rmGal-9 were used as negative control, and CMT93 wildtype cells were used a positive control. The rmGal9 endocytosis was analysed by western blotting of whole cell lysates with anti-Galectin-9 antibody.

Edu incorporation assay

For Edu incorporation assay, the protocol (https://doi.org/doi:10.1152/ajpgi.00453.2010) was performed. Briefly, mice were injected with 100 μg Edu (5-ethynyl-2´-deoxyuridine) (Invitrogen #E10187) intraperitoneally. After 3 h, mice were sacrificed and crypts were isolated. Detection of incorporated Edu was performed after fixation and permeabilization, following provided protocol (Invitrogen #C10635).

Autophagic vacuole detection

Lysosomal/autophagic vacuoles were probed by MDC (monodansylcadaverine) staining. Ileum crypts, acinar cells, CMT93, or HT-29 cells with or without manipulations were subjected to MDC staining by Autophagy Detection Kit (Abcam, #ab139484) with manufacturer instructions.

RNA extraction from the cultured organoids

To extract the RNA, organoids pellet was homogenized with 1 ml TRIzol Reagent (Ambion #15596018) and the homogenate was incubated for 5 min at room temperature. About 0.2 ml chloroform was added to the homogenate and vortexed vigorously, the samples were stored at room temperature for 15 min with occasional vortex and later centrifuged at 12,000 *x*g for 15 min at 4 ºC to extract the RNA. The aqueous phase was transferred into a new tube, mixed with 0.5 ml isopropanol and 1 μl UltraPure Glycogen carrier (Invitrogen #10814010) and stored at room temperature for 10 min to precipitate the RNA. The samples were centrifuged at 12,000 *x*g for 8 min at 4 ºC and the supernatant was discarded. The RNA pellet was washed with 1 ml 75% ethanol and centrifuged at 7,500 *x*g for 5 min at 4 ºC for three times and finally the pellet was dissolved in DEPC-H_2O and RNA concentration was measured.

Epithelial crypt cell dissociation for flow cytometry

Crypts were isolated and washed twice in cold PBS as described above. Crypt pellets were resuspended in HBSS with 0.5 U/ml Dispase (Corning, #354235) and incubated at 37 °C for 10 min with 100 rpm shaking. To stop the digestion, 5% FBS in HBSS with 100 μg DNase I

(Sigma #DN-25) was added to the crypt suspension and then passed through the 70 μm cell strainer. The dissociated crypt single cells were pelleted by centrifugation at 500 *x*g for 5 min and were washed twice with flow staining buffer (FBS + 5% fetal calf serum). The dissociated cells were stained with surface antibody markers for 15 min at 4 °C and later fixed with 4% paraformaldehyde for 15 min at room temperature. After washing with staining buffer, cells were resuspended in Intracellular Permeabilization Buffer (Invitrogen #PB001) to permeabilize the cells for intracellular staining. The cells were incubated with staining buffer containing antibodies for intracellular staining at room temperature for 30 min in dark. The cells were again washed with staining buffer and then analyzed by flow cytometry.

Preparation of samples for cDNA synthesis and qPCR

Mice were euthanized, intestines collected, longitudinally opened, and immediately washed with ice-cold PBS. Crypts isolation and organoid culture was done as previously described. A fragment of the intestine was cut horizontally for further analysis and the intestinal epithelium was collected by gently scraping with glass slides and homogenized in TRIzol Reagent. Total RNA collected from the above samples were reverse transcribed by using iScript™ cDNA Synthesis Kit (Bio-Rad #1708891). cDNA was amplified by using Power SYBR® Green PCR Master Mix (Applied Biosystems #4367659) with manufacturer instructions. Data were collected by ABI StepOnePlus Real-Time PCR Systems with ABI StepOne Software (v2.3). House-keeping gene L32 was used as internal control for normalization.

Co-Immunoprecipitation and Western blotting

To perform co-Immunoprecipitation, Dynabeads® Co-Immunoprecipitation Kit (Life Technologies #14321D) was used. The 5 μg antibody (mouse monoclonal Anti-FLAG® M2 antibody, Sigma #F1804) was conjugated to 1.5 mg dynabeads as per the manufacturer's instructions. 50 mg of compact cells are lysed in 450 μl extraction buffer A containing 100 mM NaCl and 1*x* Protease inhibitor cocktail without EDTA (MCE #HY-K0010) and incubated on ice for 15 min. Then centrifuged at 2,600 *x*g for 5 min at 4 °C to remove large cell debris and nuclei. The supernatants were re-suspend with antibody coupled Dynabeads and incubated on a roller at 4 °C for 30 min. The beads were washed three times with extraction buffer A on magnetic stand. The Dynabeads® Co-IP complexes were washed with 1*x* LWB for one time. The beads were resuspend in 60 μl Elution Buffer and incubate on a roller at RT for 5 min to elute the Co-IP complexes. The elution product was dried out in speed vac to remove all the elution buffer. The samples were mixed with 1*x* NuPAGE™ LDS Sample Buffer (Invitrogen™ #NP0008) containing 1*x* NuPAGE™ Sample Reducing Agent (Invitrogen™ #NP0009) and samples were heated at 70 °C for 10 min. The samples were either loaded into NuPAGE™ 4-12% Bis-Tris Protein Gels (Invitrogen™ #NP0322PK2) or NuPAGE™ 3-8% Tris-Acetate Protein Gels (Invitrogen™ #EA03752PK2) and gel electrophoresis was run using XCell SureLock Mini-Cell. The bands in the gel were visualized by SYPRO ® Ruby Protein Gel Stain (Molecular probes #S12000) before the Co-IP complexes were analysed by mass spectrometry at the core facility, Institute of Biomedical sciences, Academia sinica. For immunoblotting the proteins were electro-transferred to polyvinylidene fluoride membrane after electrophoresis using XCell II Blot Module. The membrane was blocked with 5% skim milk and was probed using specific antibodies followed by incubation with the appropriate HRP-labelled secondary antibodies. The protein bands were detected using an T-Pro LumiLong Plus Chemiluminescence Detection Kit (T-Pro Biotechnology #JT96-K004M) and

the digital images were captured using ImageQuant LAS 4000 mini (GE Healthcare Life Sciences) Luminescent Image analyser.

Generation of CMT93 knockout cells by CRISPR-Cas9

sgRNA design and cloning were performed according to Ran et al's method (Nature Protocols 8:2281, 2013) using an online CRISPR design tool (http://crispr.mit.edu). The sequences of the oligos for galectin-9 are as follows: 5'-caccgccacttcaacccccggtttg-3' and 5'-aaaccaaaccgggggttgaagtggc-3'. The sequences of oligos for Lamp2 are as follows:5'-caccgacacgatggaagcagttgtg-3' and 5'- aaaccacaactgcttccatcgtgtc-3'. The sequences of oligos for Lamp1 are as follows: 5'- caccgaccattcgcagtctcgtagg-3' and 5'-aaaccctacgagactgcgaatggtc. Targeting sequences were cloned into BbsI-digested pSpCas9(BB)-2A-GFP plasmid (Addgene, px458), which was a gift from Dr. Liuh-Yow Chen in Academia Sinica, Taiwan. The px458-based constructs were introduced into CMT93 cells by PolyJet^{TM} In Vitro DNA Transfection Reagent using advanced protocol (SignaGen Laboratories #SL100688). Single GFP^{+} cells was sorted into 96-well plate to obtain single cell clone by FACS Aria IIIu cell sorter (BD Biosciences). Gal-9-deficient CMT93 cells were screened and confirmed by intracellular Gal-9 staining analyzed flow cytometry and immunoblotting. *Lamp1*/*Lamp2* double knock out CMT93 cells were screened by immunoblotting.

Stable reconstitution of CMT93 cells

Galectin-9-deficient CMT93 cells were reconstituted with Flag-Galectin-9 and its mutants, whereas *Lamp1* and *Lamp2*-double knockout CMT93 cells were reconstituted with Lamp2b-V5 and its mutants, respectively. To express the cDNA, recombinant lentivirus were produced in HEK293T cells, by co-transfecting cDNA expressing lentiviral vectors (2.5 μg) pLAS5w.Flag-Galectin-9.Ppuro or pLAS2w.Lamp2b-V5.Pbsd with packaging vectors such as pCMV-ΔR8.91 (2.25 μg) and pMD.G (0.25 μg) by Turbofect Transfection Reagent (Thermo scientific #R0531) in a 6 cm dish. After 72 h, the medium was harvested, aliquoted and stored at −80 °C. CMT93 cells were infected with recombinant lentiviruses encoding specific gene with an MOI>5 in the presence of 8 μg/ml polybrene for 24 h. The cells were selected in the growth medium containing 2 μg/ml of puromycin for 5 days or 10 μg/ml of Blasticidin for 10 days. The antibiotic-resistant cells were collected and the gene expression in those cells were determined by immunoblotting with the specific antibody.

Silencing the gene expression by using small interfering RNA

To inhibit the protein expression, recombinant lentiviruses encoding siRNAs specific to the gene of interest were purchased from RNAi core, Academia Sinica, Taiwan. The target sequence for mouse *Lamp1* (NM_010684) siRNA was 5'-GCAGTTCTTGTGGTAAAGAAA-3' (Clone ID: TRCN0000086784), mouse *Lamp2* (NM_010685) siRNA was 5'-GCGTTTCAGATCAACACCTTT-3' (Clone ID: TRCN0000086974), mouse Igf2r (NM_010515.2) siRNA were 5'-CGGAGGAAATACTACCTCAAT-3' (Clone ID: TRCN0000119863) or 5'-CGACCTATAAGAAGCCTTAAT-3' (Clone ID: TRCN0000119862), mouse *β3gnt2* (NM_016888) siRNA was 5'-CCCAGCAGTATTGTCCTTGTT-3' (Clone ID: TRCN0000332723), mouse *β4galt1* (NM_022305) siRNA was 5'-GCAATGGACAAGTTCGGGTTT-3' (Clone ID: TRCN0000018768), and human *LGALS9* (NM_002308) siRNA was 5'-CGGACTTCAGATCACTGTCAA-3' (Clone ID:

TRCN0000057443) respectively. The above-mentioned sequences were respectively cloned into the EcoRI site of the lentiviral vector pLKO.1-puro. To produce recombinant lentiviruses, HEK 293T cells were co-transfected with vector alone or pLKO.1-siRNA insert (2.5 µg), pCMV-ΔR8.91 (2.25 µg) and pMD.G (0.25 µg) by Transfection Reagent in a 6 cm dish. After 72 h, the medium was harvested, aliquoted and stored at −80 °C. To silence the gene expression in CMT93 or HT-29 cells, they were infected with recombinant lentiviruses encoding gene specific siRNAs with an MOI>6 in the presence of 8 µg/ml polybrene for 24 h. The cells were selected in the growth medium containing 2 µg/ml of puromycin for 5 days. The puromycin-resistant cells were collected and the gene knockdown in those cells was determined by immunoblotting with the specific antibody.

Construction of Flag-tagged galectin-9 cDNA

The mouse *galectin-9* cDNA (NCBI accession no: NM_010708.2, longer isoform; NM_001159301.1, shorter isoform) were cloned inframe at NheI and EcoRI sites of the pLAS5w.Ppuro lentiviral vector (RNAi core, Academia Sinica, Taiwan, #C6-8-39) containing Flag epitope towards N-terminal at Bsu36I and NheI sites. The primers for cloning *galectin-9* cDNA were forward primer 5' GCT AGC ATG GCT CTC TTC AGT GCC 3' and reverse primer 5' AGA ATT CCT CTA TGT CTG CAC GTG GGT C 3'. To create point mutations QuikChange II XL Site-Directed Mutagenesis Kit (Agilent Technologies, Inc. #200521) was used. The arginine at 64 in both longer and short isoform, arginine at 269 in longer isoform or 238 in shorter isoform were mutated to alanine. The primers used to mutate the site at 64 were Forward primer 5' CTT CCA CTT CAA CCC CGC GTT TGA GGA AGG AGG G 3', Reverse primer 5' CCC TCC TTC CTC AAA CGC GGG GTT GAA GTG GAA G 3'; and the primers used to mutate 269 or 238 were Forward primer 5' GCT TTC CAC CTG AAC CCC GCT TTC AAT GAG AAT GCT G 3', Reverse primer 5' CAG CAT TCT CAT TGA AAG CGG GGT TCA GGT GGA AAG C 3' respectively. The bases underlined were mutated. The double mutant is named as RA2.

Construction of Lamp2b-V5 tagged cDNA

The mouse *Lamp2b* cDNA (NCBI Reference Sequence: NM_001290485.2) was cloned inframe at NheI and AfeI sites of the pLAS2w.Pbsd lentiviral vector (RNAi core, Academia Sinica, Taiwan, #C6-8-24) containing V5-tagged epitope towards C-terminal at AgeI and EcoRI sites. The primers used for cloning *Lamp2b* cDNA were forward primer 5' GCT AGC GCC ACC ATG TGC CTC TCT CCG GTT AAA GGC 3' and reverse primer 5' AGC GCT GAC AGA CTG ATA ACC AGT ACG ACT TTT CCG 3'. To inhibit the glycosylation 7 asparagine (N) residues were mutated to aspartic acid (D).

The following are the list and the bases underlined were mutated:

(01) *Lamp2* N97D (the Asparagine AAT is mutated to Aspartic acid GAT)

Forward: 5′CT GTC TCT TGG GCT GTG GAT TTT ACC AAG GAA GC

Reverse: 5′GCT TCC TTG GTA AAA TCC ACA GCC CAA GAG ACA G

(02) *Lamp2* N115D (the Asparagine AAC is mutated to Aspartic acid GAC)

Forward: 5′GAC ATC GTG CTT TCC TAC GAC ACT AGT GAT AGC ACA G

Reverse: 5′CTG TGC TAT CAC TAG TGT CGT AGG AAA GCA CGA TGT C

(03) *Lamp2* N29D (the Asparagine AAT is mutated to Aspartic acid GAT)

Forward: 5′G TCC AAT GCA TTG ATA GTT GAT TTG ACA GAT TCA AAG GG

Reverse: 5′CCC TTT GAA TCT GTC AAA TCA ACT ATC AAT GCA TTG GAC

(04) *Lamp2* N45D (the Asparagine AAT is mutated to Aspartic acid GAT)

Forward: 5′C CTT TAT GCA GAA TGG GAG ATG GAT TTC ACA ATA ACA TAT GAA AC

Reverse: 5′GTT TCA TAT GTT ATT GTG AAA TCC ATC TCC CAT TCT GCA TAA AGG

(05) *Lamp2* N54D (the Asparagine AAC is mutated to Aspartic acid GAC)

Forward: 5′CACA ATA ACA TAT GAA ACT ACA GAC CAA ACC AAT AAA ACT ATA ACCATTG

Reverse: 5′CAATGG TTA TAG TTT TAT TGG TTT GGT CTG TAG TTT CAT ATG TTA TTGTG

(06) *Lamp2* N57D (the Asparagine AAT is mutated to Aspartic acid GAT)

Forward: 5′CA TAT GAA ACT ACA AAC CAA ACC GAT AAA ACT ATA ACC ATT GCA GTA C

Reverse: 5′GTA CTG CAA TGG TTA TAG TTT TAT CGG TTT GGT TTG TAG TTT CAT ATG

(07) *Lamp2* N175D (the Asparagine AAT is mutated to Aspartic acid GAT)

Forward: 5′CAC CTG CAA GCT TTT GTC CAA GAT GGT ACA GTG AGT AAA AAT G

Reverse: 5′CAT TTT TAC TCA CTG TAC CAT CTT GGA CAA AAG CTT GCA GGT G

Oxidative stress induced apoptosis

Reactive oxygen species (ROS) were generated during protein folding and activate ER stress response. Persistant oxidative stress and unsolved ER stress leads to apoptotic cascade in cell. To test the role of galectin-9 in ER stress mitigation, we challenged CMT93 cells with 750 μM H_2O_2 for 30 min and subjected to viability assay (eBioscience, #65-0865-14) and analyzed by Flow Cytometry.

Perinuclear puncta formation assay

CMT93 cells were grown on sterile silane coated glass slides for 24~48 h and then 2.5 mM of Leu-Leu methyl ester hydrobromide (LLOMe) (Sigma #L7393) was endocytosed for 3 h to induce endosomal/lysosomal damage. This damage induces the formation of autophagosomes which move towards the perinuclear region to fuse with functional lysosomes to form autolysosome to clear the damaged organelles. Later, the cells were fixed and the stained with antibodies.

Immunofluorescence staining of ileum organoids

About ~250 ileal crypts per well were mixed with 25 μl Matrigel containing 1 μM Jagged-1 peptide and plated into an 8-well chamber slide (Lab-Tek II, #154534). The Matrigel was polymerized for 10 min at 37 ºC incubator, and 400 μl ENR growth media was added on top of the Matrigel. To prevent anoikis 10 μM ROCK inhibitor, Y-27632, was added to the growth media for the first 2 days of the culture. Media was replaced every 2 days. Along with medium changes, treatment wells of ileal organoids received 0.5 μg/ml rmGal-9 protein for 6 days. To fix the organoids the growth media was aspirated, the chambers were washed with PBS twice and 400 μl freshly prepared 4% PFA-PME [4% (w/v) paraformaldehyde, 50 mM PIPES, 2.5 mM $MgCl_2$, and 5 mM EDTA] was added to each well for 20 min at room temperature. The chambers were washed with PBS twice and then the fixed organoids were incubated with 200 μl NH_4Cl (50 mM in PBS) for 30 min at room temperature to quench autofluorescence. The chambers were washed again with PBS twice and the organoids were permeabilized with 0.5% Triton X-100 in PBS for 30 min. For immunostaining, the chambers were washed with PBS twice, blocked in blocking buffer (1*x* PBS, 5% normal goat serum, 0.3% Triton X-100) for 1 h, and later the blocking buffer was replaced with 200 μl primary antibody in antibody dilution buffer (1*x* PBS, 1% BSA, 0.3% Triton X-100) and incubated overnight at 4 °C. After incubation,

the primary antibodies were aspirated and the chambers were washed with wash buffer (PBS, 0.1% Tween-20) three times and then the organoids were incubated with 200 μl fluorophore-conjugated secondary antibody in antibody dilution buffer for 2 h at room temperature. The secondary antibodies were aspirated and the chambers were washed with wash buffer three times. When applicable the organoids were double stained. The nucleus was visualized by counterstaining with 200 μl Hoechst 33342 (2 μg/ml in PBS) (Molecular Probes #H3570) for 10 min at room temperature and the chambers were removed after washing with wash buffer three times. The cover slips were mounted on the slides by using ProLong Gold Antifade Mountant (Molecular Probes #P36934). Confocal images were obtained with a Carl Zeiss LSM 700 stage laser scanning microscope imaging system under a 40*x* oil-immersion objectives with ZEN 2011 software. For imaging comparisons between wild-type and *galectin-9*$^{-/-}$ organoids that had undergone different treatments, samples were processed side by side for the whole procedure.

Human tissue array

The colorectal carcinoma tissue specimens and IBD tissue specimens were obtained from SuperBioChips Laboratories (Seoul, Korea) or from US Biomax, Inc. (Maryland, US), respectively. Galectin-9 expression score was assessed using H-score system. By taking into account both the staining intensity and the percentage of the surface area, the total intensity for galectin-9 staining evaluated as negative, 0; weak, 1+; moderate, 2+; or strong, 3+. H-score (0-300) = 0 * (%cells with 0 intensity) + 1 * (%cells with 1+ intensity) + 2 * (%cells with 2+ intensity) + 3 * (%cells with 3+ intensity)

Lysosome acidity measurement by Lysotracker DND-26

Lysosome acidity in CMT93 cells were measured by Lysotracker DND-26 staining (invitorgen, #L7526). The cells were incubated with 15 nM Lysotracker DND-26 in flow staining buffer at 37ºC for 15 min, later washed with PBS and stained with surface antibody markers if mentioned for 15 min at 4 °C and analysed by flow cytometry. To explore the lysosomal regulation mediated by Gal-9, CMT93 cells were treated with 10 nM Rapamycin for overnight, 100 μM Forskolin for 30 min, or with 0.5 μg/ml recombinant mouse galectin-9 protein for 2 days and subjected to lysotracker staining.

Mass Spectrometry

Protein Digestion: The protein samples were dissolved in 6 M urea and reduced with 20 mM DTT for 1 h at 60 °C before they were alkylated using 55 mM iodoacetamide for 45 min in the dark at RT. The sample solutions were diluted to 1 M urea by 50 mM TEABC buffer and were digested with trypsin at an enzyme: protein ratio of 1:50 w/w for 16-18 h at 37 °C. Digested peptides were desalted with C18 Ziptip (Millipore, MA) according to the instructions of the manufacturer, and then analyzed by LC-MS/MS.

Protein identification by LC-MS/MS: LC-MS/MS analysis was performed on a NanoACQUITY UPLC System (Waters, USA) coupled to a high-resolution mass spectrometer (Q Exactive HF-X, Thermo Fisher Scientific, USA). The tryptic peptides were injected into a trap column (2 cm *x* 75 µm i.d., Symmetry C18), then separated in a 25 cm *x* 75 µm i.d. BEH130 C18 column (Waters, USA) by a one-hour gradient from 0% to 85% buffer B (buffer A, 0.1% formic acid in H_2O; buffer B, 0.1% formic acid in acetonitrile). The mass spectrometer was operated

in data-dependent mode with the following acquisition cycle: a full scan (m/z 350–1600) recorded in the orbitrap analyzer at a resolution R = 60,000, and up to the 20 most intense peaks with charge ≥2 were selected and fragmented by HCD at resolution R = 15,000 with normalized collision energy of 28. The database search of RAW spectra was performed by Byonic search engine (v2.16.11, Protein Metrics Inc., San Carlos, CA) against the UniProtKB Mus musculus protein database using the following parameters: the mass tolerance of precursor peptide was set as 10 ppm, and the tolerance for MS/MS fragments was 0.02 Da with maximum two missed cleavage. Modifications of peptides were set as follows: static carbamidomethylation on cysteine, variable oxidation on methionine, and variable deamidation of asparagine or glutamine. The cut-off threshold of significant matches is score ≥ 300.

Heatmap of lysosomal protein quantity: The lysosomal protein coding genes were selected from the LC-MS/MS analysis and ranked by their RAW spectra values in descending order. All RAW spectra values were transformed into z-scores for creating the heatmap. The list of mouse lysosomal protein coding gene was from mLGdb v.1.2 (http://lysosome.unipg.it).

Density gradient ultra-centrifugation

For density gradient ultra-centrifugation to separate the cell organelles, the protocol with some modifications was performed (https://doi.org/doi:10.1038/ncomms9876). Briefly, PNS was prepared in 250 mM Sucrose, 10 mM Tris HCl, 1 mM EDTA, 0.03 mM Cycloheximide, 1*x* Protease inhibitors, and pH7.4. The 1 ml of PNS was mixed with 60% OptiPrep™ Density Gradient Medium (Sigma #D1556) to obtain 3 ml of 42% gradient and was placed at the bottom of polyallomer tube. On the top of 42% gradient, 1 ml of 35, 30, 25, 20, 17.5, 15, 12.5, 10, and 7.5% OptiPrep™ Density Gradient Medium (diluted in HM) was layered one above the other to obtain a step gradient. The samples were centrifuged at 125,000 *x*g (27,000 rpm), for 20 h at 4 °C, using SW 41Ti rotor in a Beckman Optima™ L-90K Ultracentrifuge. After ultracentrifugation, 500 μl per fraction was collected from the top of the ultracentrifuge tube. A total of 24 fractions were collected and immediately snap-frozen in liquid nitrogen and stored at −80 °C. The sample were thawed and fractions were concentrated in a speedVac. The protein concentration was measured by bradford method and the yield and enrichment of each fraction relative to the initial PNS was calculated. Equal volumes of each of the fractions were loaded on SDS-PAGE for western blot analysis.

Immunofluorescence staining of tissue sections and cells

Mouse pancreas or ileal tissues were fixed in freshly prepared 4% formaldehyde in PBS overnight at 4 ºC, depending upon the experiment, the tissue was either dehydrated in ethanol and embedded in paraffin or dehydrated in 30% sucrose in PBS and frozen in OCT compound (FSC 22 Frozen Section Media, Leica Biosystems #3801480). Paraffin tissue slices or cryosections (6 μm in thickness) were sectioned by the Pathology Core Lab at Institute of Biomedical Sciences, Academia Sinica, Taiwan and mounted on positively charged glass slides. Paraffin sections were dewaxed and heat-mediated antigen retrieval was performed by incubation in either 10 mM sodium citrate buffer pH 6.0 for 10 min or 1 mM EDTA pH 8.0 for 5 min at a sub-boiling temperature. The freshly isolated acinar cells or CMT93 cells which were treated with 0.5 μg/ml recombinant mouse galectin-9 protein (R&D systems #3535-GA) or recombinant mouse galectin-3 protein (R&D systems #1197-GA) and treated with LLOMe

to induce perinuclear puncta were fixed in freshly prepared 4% formaldehyde in PBS for 15 min and later permeabilized with 0.3% Triton X-100 in PBS for 15 min. Slides were then blocked with blocking buffer (PBS containing 5% goat serum and 0.3% Triton X-100) for 1 h and subjected to sequential incubation with primary antibodies at 4 °C overnight and fluorophore-conjugated secondary antibodies at room temperature for 2 h. When applicable the slides were double stained. Between each incubation step, slides were washed with PBS three times for 5 min. The antibodies were diluted in antibody dilution buffer (PBS containing 1% BSA and 0.3% Triton X-100). The cryosection slides prepared from Lgr5-eGFP mice were brought to room temperature and washed with de-ionized water to remove OCT compound. To visualize the nucleus, slides were counterstained with Hoechst 33342 (1 μg/ml in PBS) (Molecular Probes #H3570) for 10 min and cover slips were mounted on the slides by using ProLong Gold Antifade Mountant (Molecular Probes #P36934). Confocal images were obtained with a Carl Zeiss LSM 700 stage imaging system under a 60*x* oil-immersion objectives with ZEN 2018 software. For direct comparisons between wild-type and galectin-9-deficient tissues or cells, we performed the whole procedure side by side from the point of tissue harvesting.

Electron microscopy analysis

Pancreatic tissue sections or distal ileal tissue sections from euthanized mice or CMT93 cells were washed with PBS and fixed in PBS containing 4% PFA and 2.5% glutaraldehyde at 4 ºC overnight. Ethanol gradient dehydration followed by infiltration and embedment were performed by the Electron Microscopy Core Facility at the Institute of Biomedical Sciences, Academia Sinica, Taiwan. Ultrathin sections were cut on a Leica Ultracut Ultramicrotome (Leica EM UC6) and collected on copper grids. The sections were double-stained with uranyl acetate and lead citrate, and then digital images were obtained under a transmission electron microscope (FEI TECNAI G^2 F20 S-TWIN) at 120 kV. The major ultrastructural differences between lysosomes of cells with or without galectin-9 were quantified. The lysosomes with onion-like membranous conformations in their lumen were labelled as typical morphology. The lysosomes that were partially filled with unprocessed subvacuolar material as well as onion-like membranes were labelled as lysosomes with partial degradative defect, whereas lysosomes that displayed accumulated undigested material in their lumen were labelled as aberrant lysosomes.

Immunohistochemistry

Harvested pancreas were immediately fixed in 10% formalin overnight and embedded in paraffin. After deparaffinizing and rehydrating the tissue section, antigen retrieval was performed using 10 mM citrate buffer (pH 6.0) for 20 min in a pressure cooker. The sections were then incubated with 3% hydrogen peroxide for 10 min and permeabilized with 0.025% (V/V) Triton X-100 in PBS for 15 min. Non-specific signals were blocked using 2% blocking reagent (Roche) and then incubation with primary antibodies. After overnight incubation, the slides were incubated with secondary antibody (Dako, EnVision) for 1 h, and then stained with DAB substrate (Dako) and counter stained with haematoxylin.

Subcellular fractionation of acinar cells

Freshly isolated pancreatic acini were resuspended in 1 ml ice-cold homogenization buffer containing 240 mM sucrose, 5 mM MOPS, and 1 mM MgSO4 (pH 6.5) containing 1*x* Protease

Inhibitor Cocktail. The acini were homogenized by using 25 strokes of the pestle of a tight-fitting Dounce homogenizer. After 90% of cell breakage, the fractions were separated by density centrifugation. The post-nuclear supernatant (PNS) was separated by 150 *x*g centrifugation for 10 min at 4 °C. The zymogen granule-enriched fraction was centrifuged at 500 *x*g for 15 min at 4 °C, and the large debris was removed by centrifugation at 2,600 *x*g for 10 min at 4 °C, followed by lysosome-enriched fraction at 15,000 *x*g for 20 min at 4 °C. The remaining supernatant was cleaned up by centrifugation at 150,000 xg for 1 h at 4 °C in Beckman Coulter Optima MAX-XP Ultracentrifuge and was used as the cytoplasmic fraction. All the pelleted fractions were washed with HM three times and were lysed in 1% NP-40 lysis buffer containing 1*x* Protease Inhibitor Cocktail and analysed by western blotting.

Trypsin activity assay

Trypsin activity of freshly isolated acinar cells were assessed by measuring 7- Amino-4-methylcoumarin (AMC) release from synthetic substrates Boc-Gln-Ala-Arg-AMC (Enzo, BML-P237-0005), a specific substrate for trypsin. The assay mixture consisted of 5 μl of 5 mM stock of the substrate, 50 μl of sample and 145 μl of 100 mM Tris HCl (pH 8.5) containing 100 μg/ml bovine serum albumin in microfluor 96-well black microtiter plates (Thermo Scientific). After 1 h incubation, the released fluorescence was detected by Gemini EM Microplate Reader (Molecular Devices) with excitation at 360 nm and emission at 480 nm.

Amylase activity assay

α-Amylase activities of freshly isolated acinar cells were determined using EnzChek Ultra Amylase assay kit (E33651, Invitrogen) in a 96-well plate fluorimeter according to the manufacturer's instructions. After 30 min incubation, fluorescence was measured by Gemini EM Microplate Reader (Molecular Devices) with excitation at 485 nm and emission at 535 nm.

Serum amylase quantification

Mouse serum was collected and serum amylase level were measured by Fuji Dri-Chem Clinical Chemistry Analyzer FDC 4000i. Experiments were performed by Taiwan Mouse Clinic, National Comprehensive Mouse Phenotyping and Drug Testing Center.

Sirius Red Staining

To detect the fibrosis in Pancreas, the paraffin tissue sections were de-waxed and then hydrated. The nuclei were stained with Weigert's haematoxylin for 8 min, and the slides were washed for 10 min in running tap water. To stain the collagen the slides were incubated with picro-sirius red solution for 1 hour (Sirius Red, Sigma #365548; Picric acid, 1.3% in water, Sigma #P6744-1GA). The slides were washed twice in acidified water (5 ml acetic acid (glacial) in 1 liter of distilled water). Later, most of the water from the slides was removed by vigorous shaking. The sections were dehydrated in three changes of 100% ethanol, cleared in xylene, and mounted in a resinous medium. In the bright-field microscopy collagen is visualized as red color on a pale yellow/green background, and nuclei as black.

Reproducibility

The definition of symbols or sample numbers (n) in each figure is as follows.

In Fig.1b, “n” indicates the number of transmission electron microscopy images taken from two mouse. Results shown were representative data from two mice in each group from two independent reproducible experiments. In Fig. 1c and 1h, “n” indicates the total number of mice in experiments. Results shown were combined data from two (1c) or three (1h) independent reproducible experiments. In Fig.1e, “n” indicates fresh crypts isolated from one mouse. The symbol in Fig.1e indicates the average of fluorescence intensity calculated from at least 10 immunofluorescence images taken from crypts isolated from one mouse. Results shown were representative data from two independent experiments. In Fig.1i, “n” indicates one well of organoid culture which was derived from one mouse, so “n” here also indicates one mouse. Results shown were representative data from two independent reproducible experiments.

In Fig.2b, “n” indicates fresh crypts isolated from one mouse. Results shown were combined data from two independent reproducible experiments. In Fig.2c, “n” indicates one well of organoid culture which was derived from one mouse, so “n” here also indicates one mouse. Results shown were representative data from two independent reproducible experiments. In Fig.2e, 2h, and 2j, “n” indicates the total number of mice in experiments. Results shown were combined data from two independent reproducible experiments. In Fig.2k, lysates of colon crypts from three Gal-9$^{F/F}$ mice (left three wells) or three Defa6-Cre^{+}Gal-9$^{F/F}$ mice (right three wells) were individually loaded for Western blot analysis.

In Fig.3a, “n” indicates the number of transmission electron microscopy images taken from three culture wells of the indicated CMT93 cells. Results shown were representative data from two independent reproducible experiments. In Fig.3c, 3d, 3h, and 3i, the symbol (3c) or “n” (3d-i) indicates one culture well of the indicated CMT93 cells. Results shown were combined (3c,3d,3h) or representative (3i) data from two independent reproducible experiments. In Fig.3f, “n” indicates the number of immunofluorescence images taken from one culture well of the indicated untreated or treated CMT93 cells. Results shown were representative data from two independent reproducible experiments.

In Fig.4f, “n” indicates one culture well of the indicated CMT93 cells. Results shown were combined data from two independent reproducible experiments. In Fig.4g, “n” indicates the number of immunofluorescence images taken from one culture well of the indicated CMT93 cells. Results shown were representative data from two independent reproducible experiments.

In Fig.5b, the symbol indicates the immunofluorescence image taken from one culture well of the indicated CMT93 cells. Results shown were representative data from two independent reproducible experiments. In Fig.5c, the symbol indicates the immunofluorescence image taken from one culture well of the indicated CMT93 cells. Results shown were representative data from two independent reproducible experiments. In Fig.5e, “n” indicates one culture well of the indicated CMT93 cells. Results shown were representative data from two independent

reproducible experiments. In Fig.5f, “n” indicates one culture well of the indicated CMT93 cells. Results shown were combined data from two independent reproducible experiments.

In Fig.6c, “n” indicates one culture well of the indicated CMT93 cells. Results shown were representative data from two independent reproducible experiments. In Fig.6e, “n” indicates one culture well of the indicated untreated or rmGal-9-treated CMT93 cells. Results shown were combined data from two independent reproducible experiments.

In Fig.7b and 7f, lysates of fresh acinar cells from three wild-type (WT) mice (left three wells) or three Gal-$9^{-/-}$ mice (right three wells) were individually loaded for Western blot analysis. In Fig.7c, the symbol indicates fresh acinar cells isolated from one mouse in one experiment. Results shown were representative data from two independent reproducible experiments. In Fig.7d, “n” indicates fresh acinar cells isolated from one mouse. Results shown were combined data from two independent reproducible experiments. In Fig.7g, “n” indicates fresh acinar cells isolated from one mouse. The symbol indicates the average of fluorescence intensity calculated from at least 10 immunofluorescence images taken from acinar cells isolated from one mouse. Results shown were combined data from two independent reproducible experiments. In Fig.7i, “n” indicates a serum sample taken from one mouse. Results shown were combined data from three independent reproducible experiments.

In Supplementary Fig.1a, “n” indicates a mRNA sample isolated from the indicated tissue samples from one mouse. Results shown were representative data from two independent reproducible experiments. In Supplementary Fig.1e and 1f, “n” indicates one colon tissue sample of an indicated patient from a commercial human tissue array.

In Supplementary Fig.2a, “n” indicates fresh crypts isolated from one mouse. The symbol indicates the average of fluorescence intensity calculated from at least 10 immunofluorescence images taken from crypts isolated from one mouse. Results shown were combined data from two independent reproducible experiments. In Supplementary Fig.2d, 2f-i, the symbol or “n” indicates fresh crypts isolated from one mouse. Results shown were combined (2d,2f) or representative (2g-i) data from two independent reproducible experiments.

In Supplementary Fig.3b and 3d, “n” indicates the number of immunofluorescence images taken from two culture wells of the indicated untreated or LLOMe-treated CMT93 cells. Results shown were representative data from two independent reproducible experiments. In Fig.s3E and s3F, “n” indicates one culture well of the indicated CMT93 cells (3e) or HT-29 cells (3f). Results shown were combined data from two independent reproducible experiments.

In Supplementary Fig.4d, 4f and 4g, “n” indicates one culture well of the indicated CMT93 cells. Results shown were representative data from two independent reproducible experiments. In Supplementary Fig.4e, the symbol indicates the immunofluorescence image taken from one culture well of the indicated CMT93 cells. Results shown were representative data from two independent reproducible experiments.

In Supplementary Fig.6c, the symbol indicates a mRNA sample from one culture well of the indicated untreated or tunicamycin-treated CMT93 cells. Results shown were combined data

from two independent reproducible experiments. In Supplementary Fig.6g, the symbol indicates one culture well of the indicated CMT93 cells. Results shown were representative data from two independent reproducible experiments.

In Supplementary Fig.7e and 7g, “n” indicates fresh acinar cells isolated from one mouse. Results shown were combined data from two independent reproducible experiments.

Uncropped blots for Figure 1.

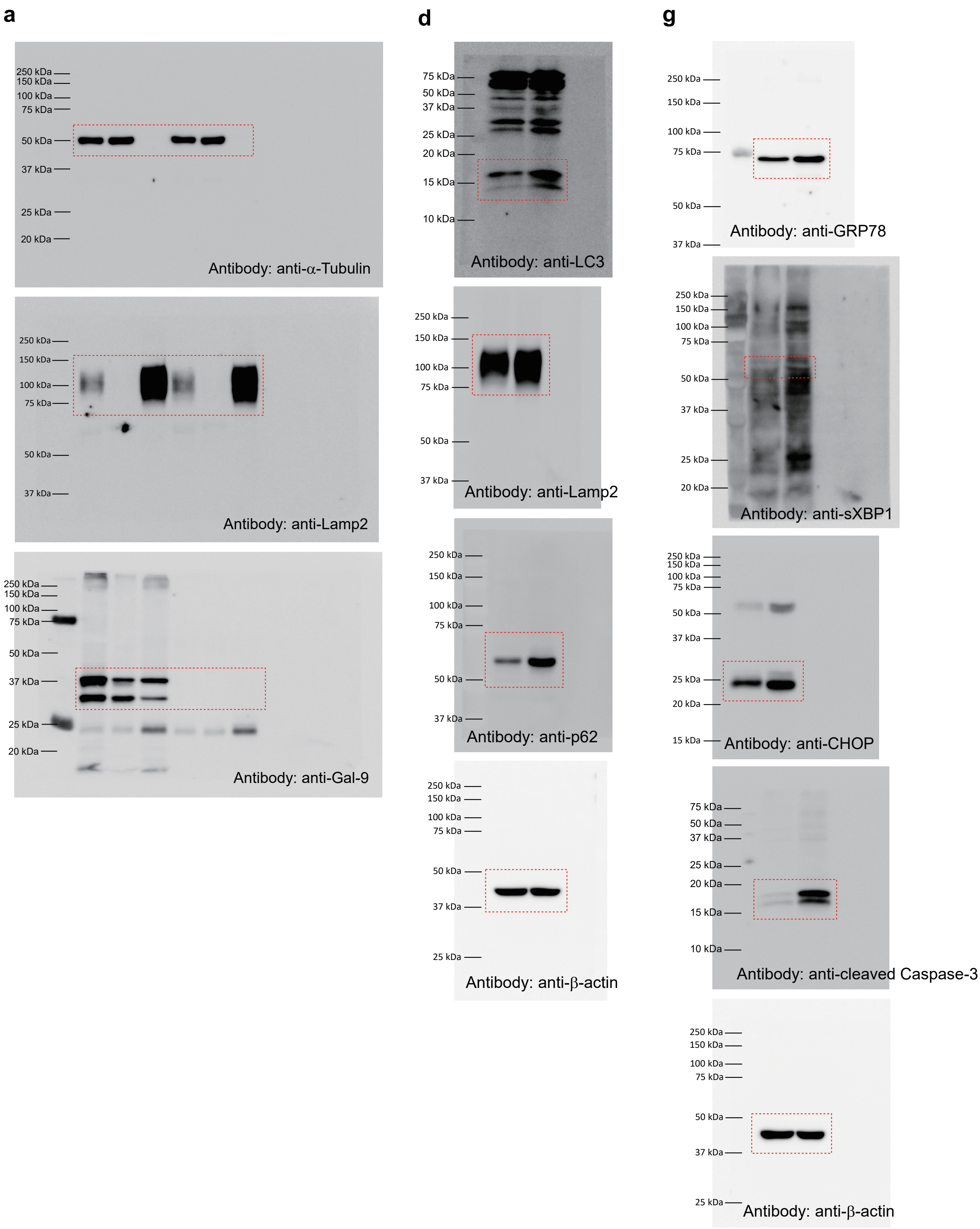

Uncropped blots for Figure 2.

k

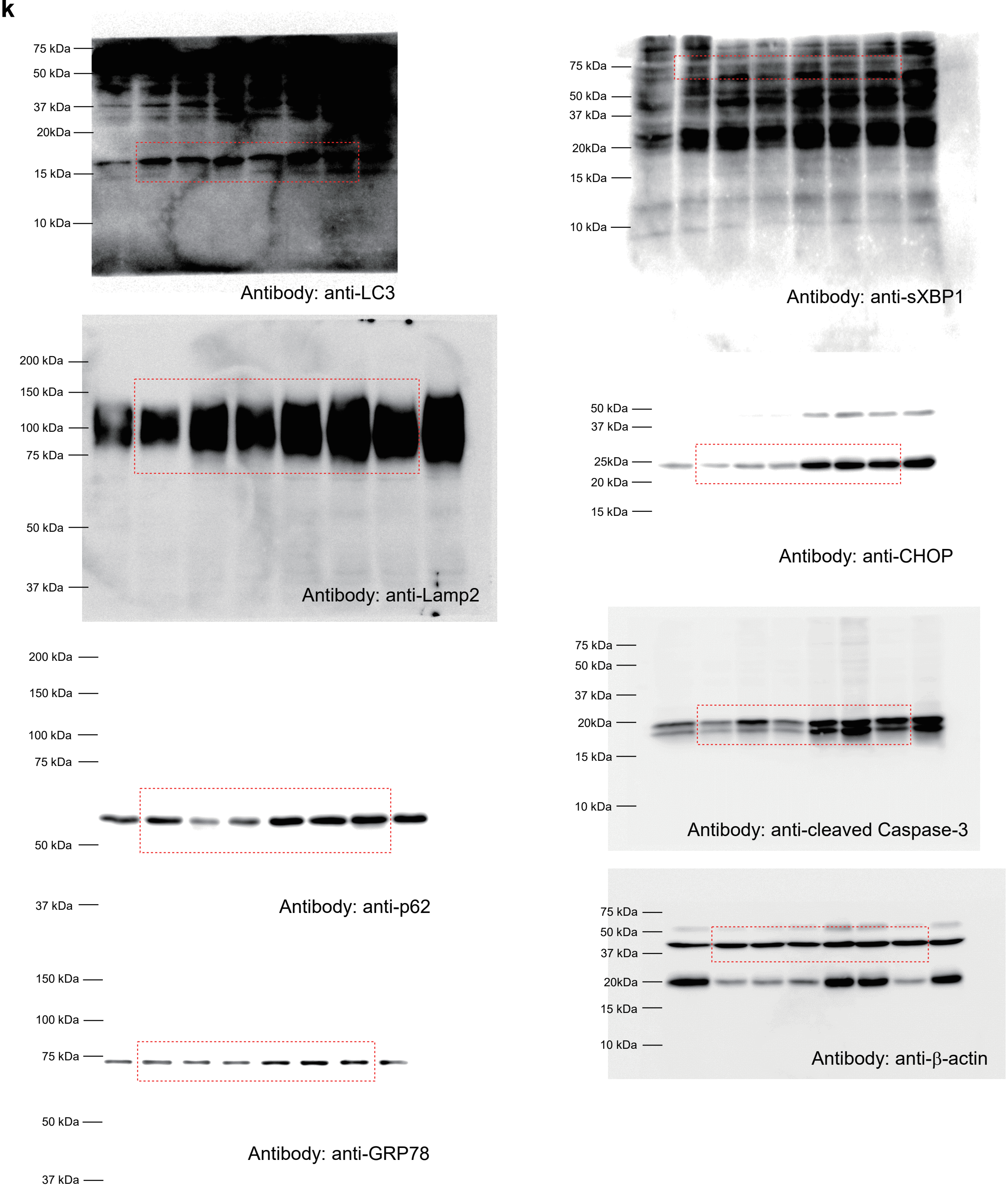

Uncropped blots for Figure 3.

b

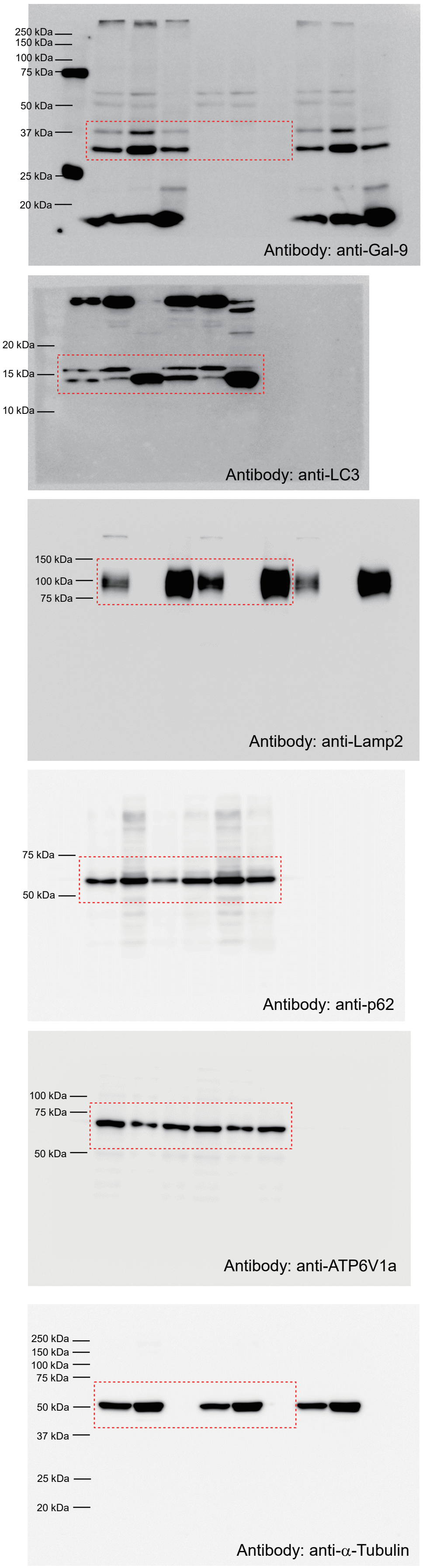

d

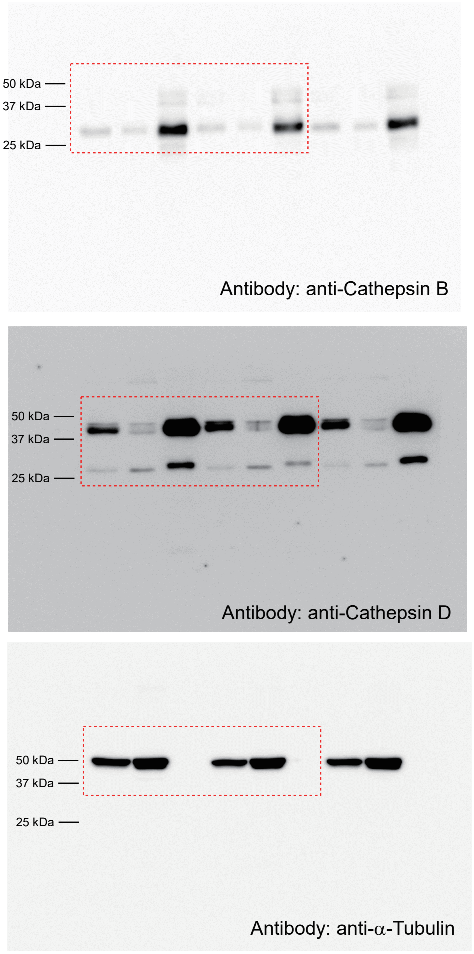

e

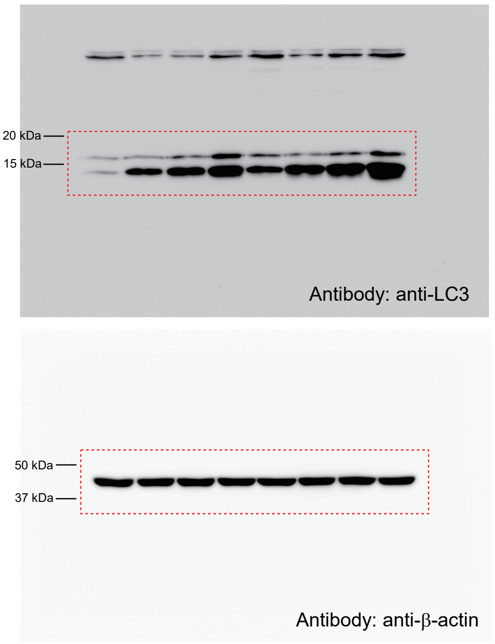

g

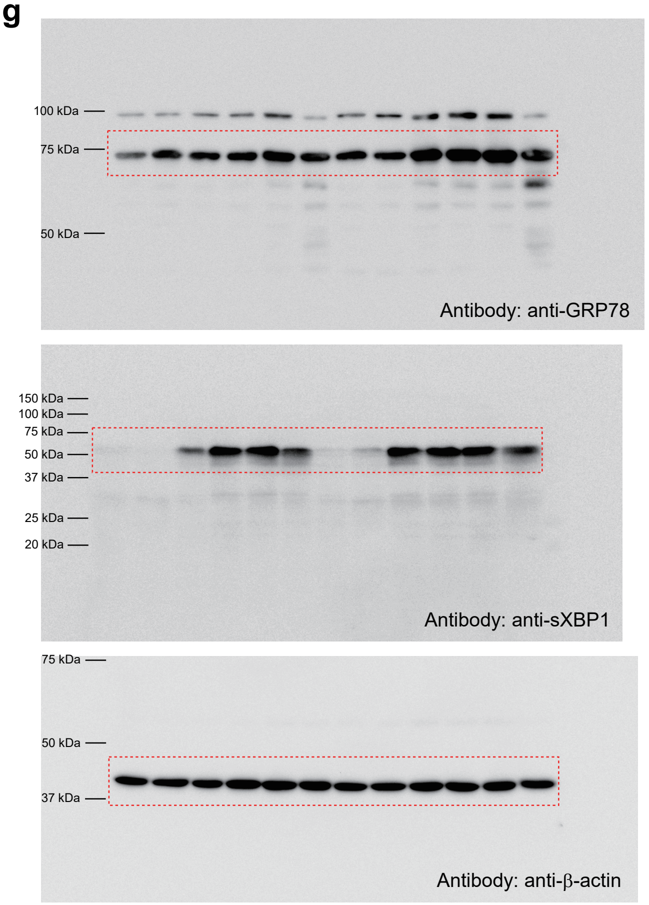

Uncropped blots for Figure 4.

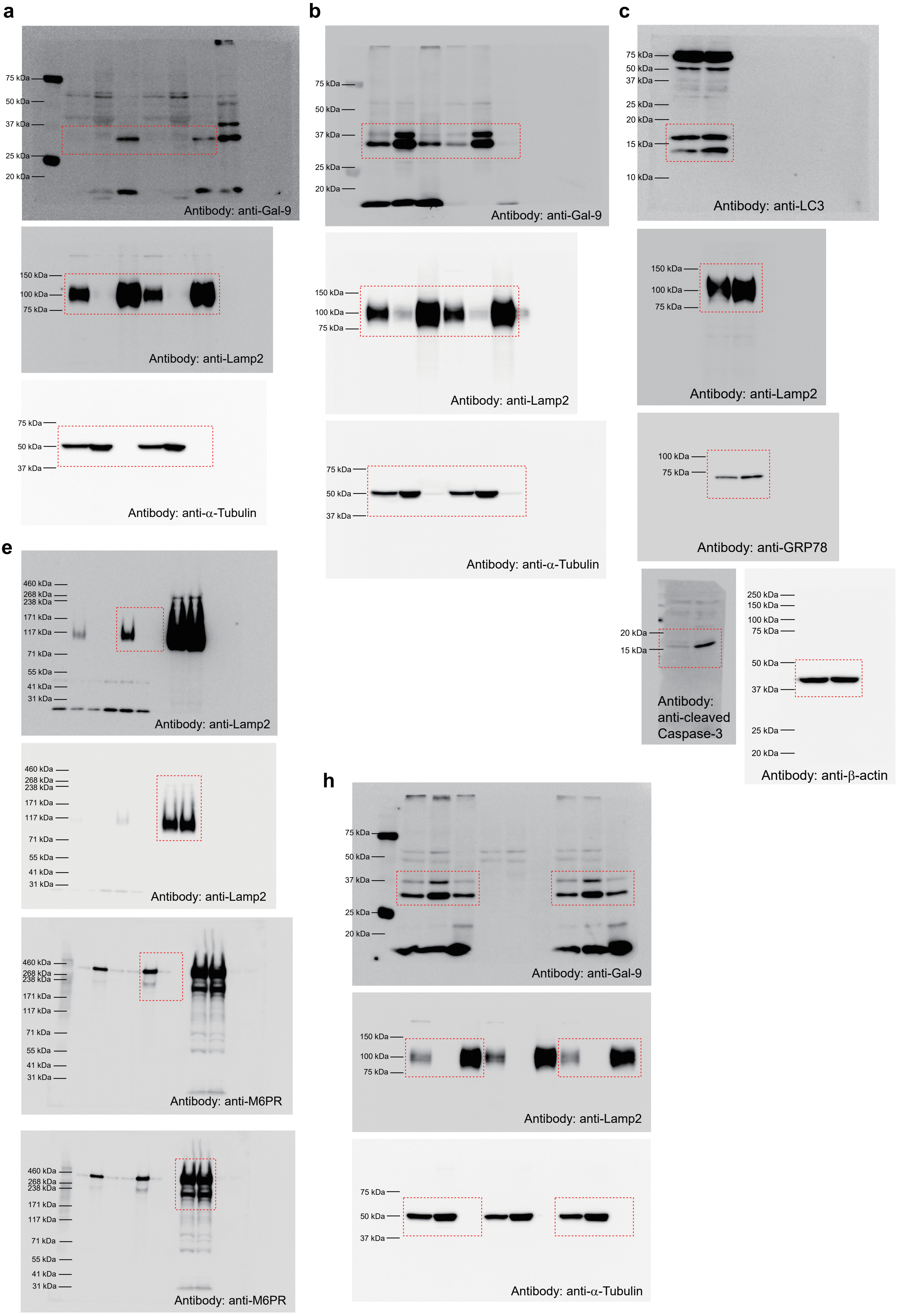

Uncropped blots for Figure 5.

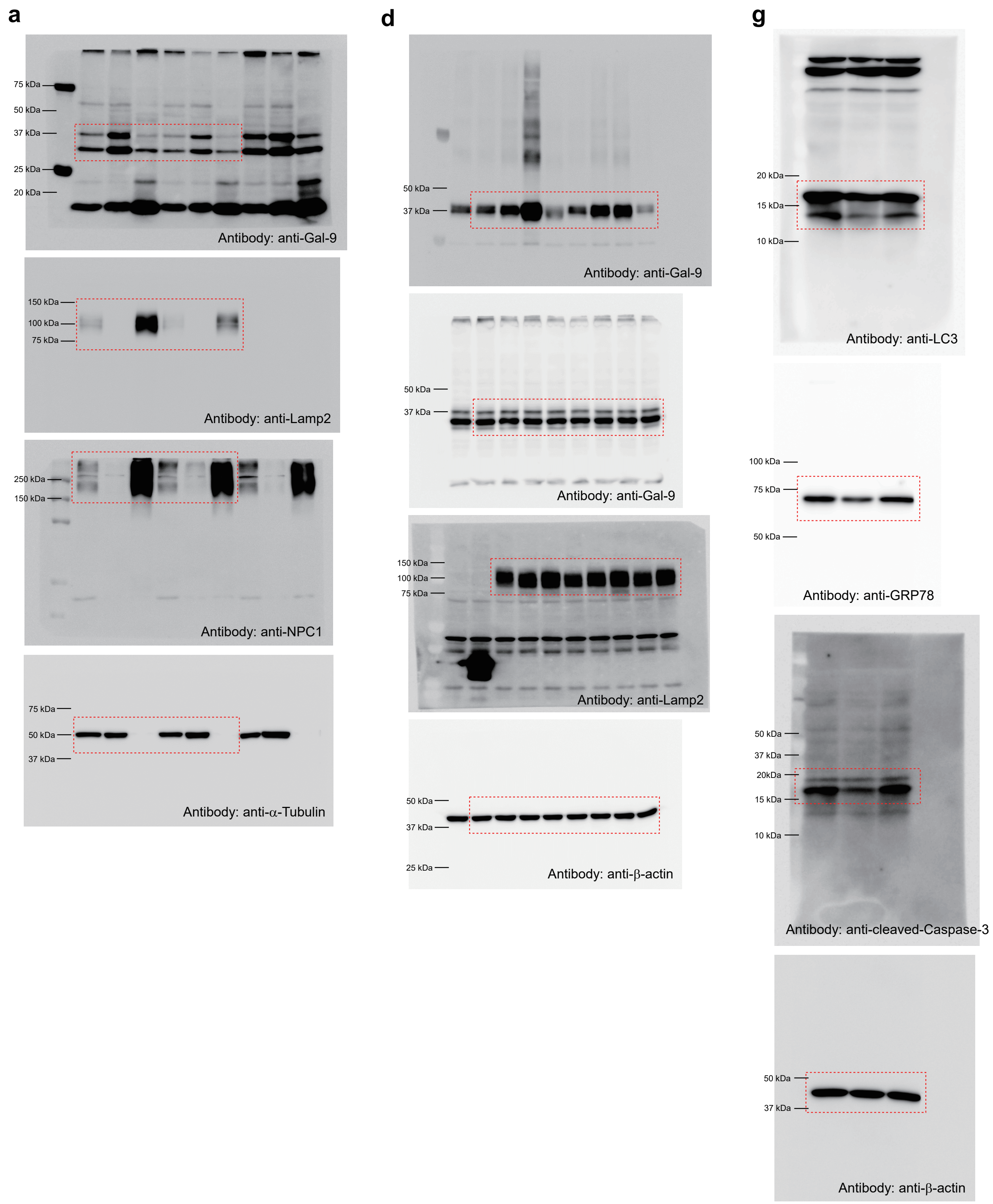

Uncropped blots for Figure 6.

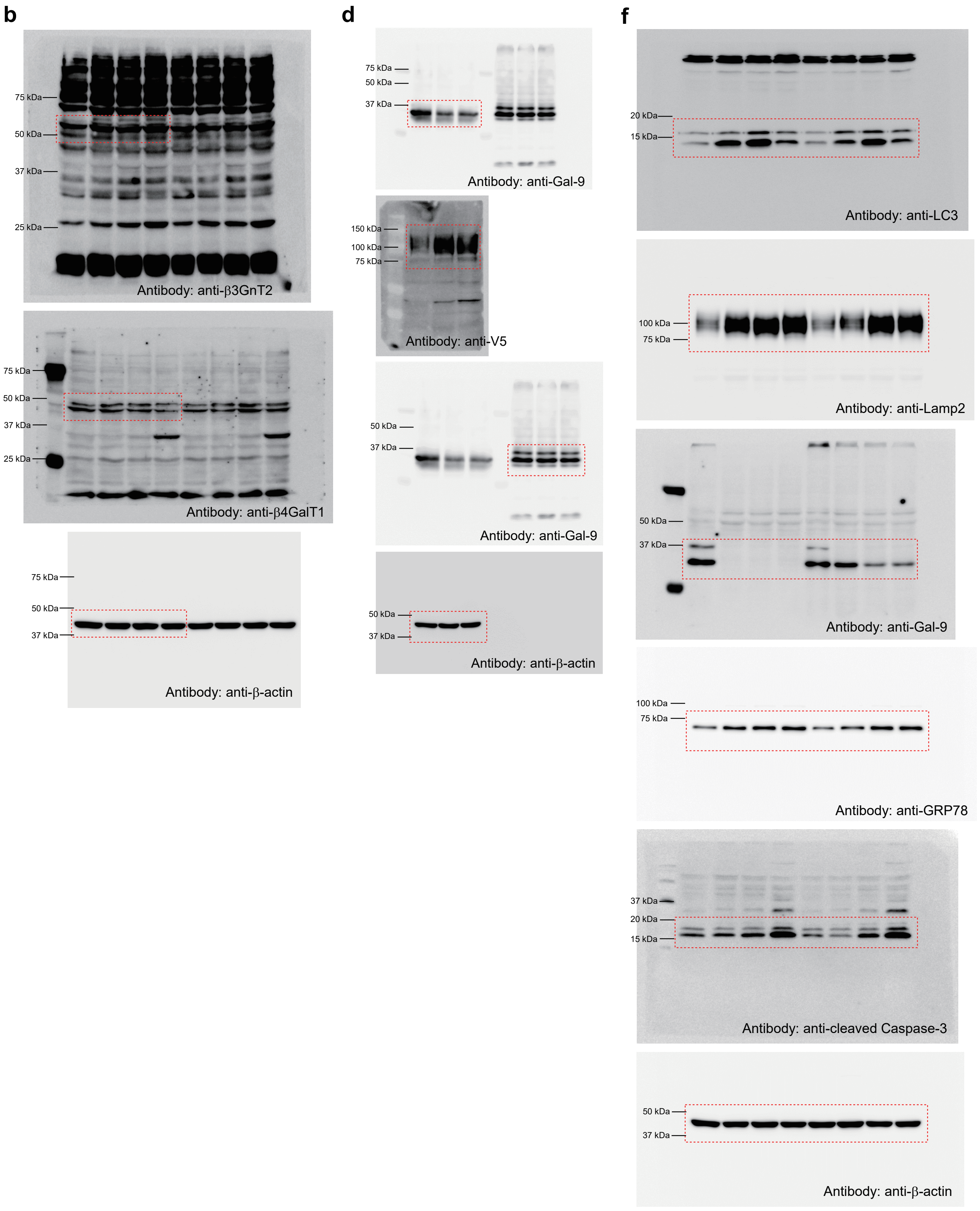

Uncropped blots for Figure 7.

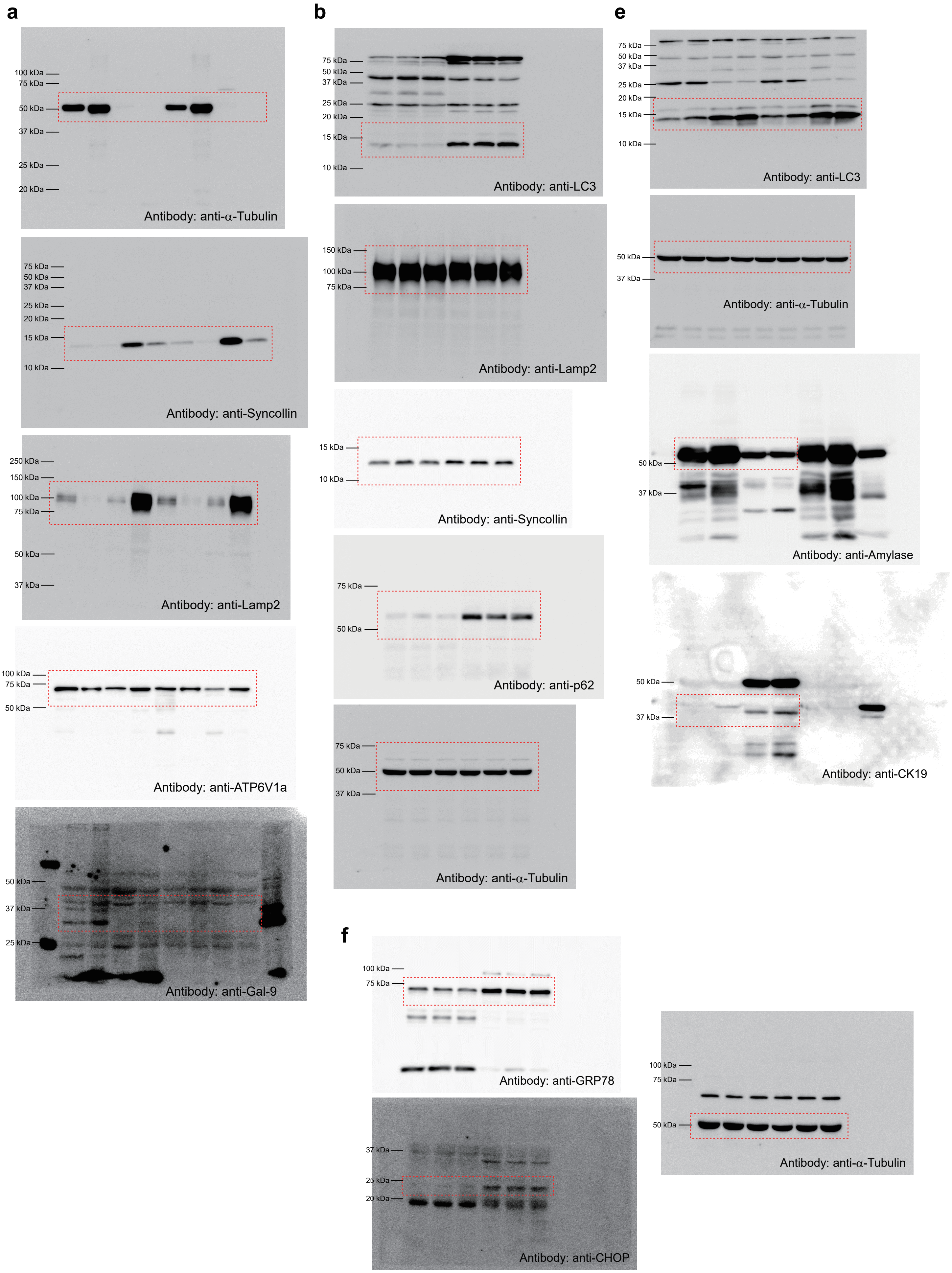

Uncropped blots for Supplementary Figure 3.

a

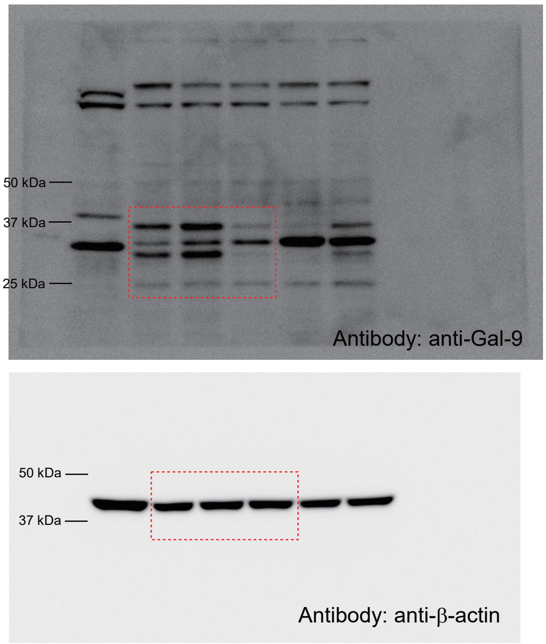

f

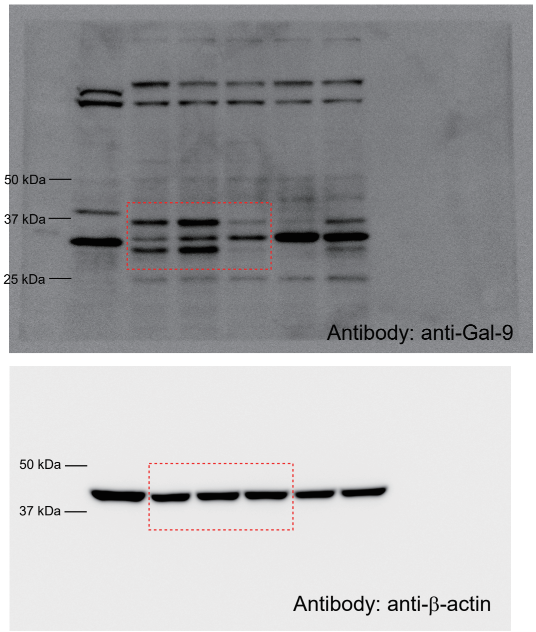

Uncropped blots for Supplementary Figure 4.

a

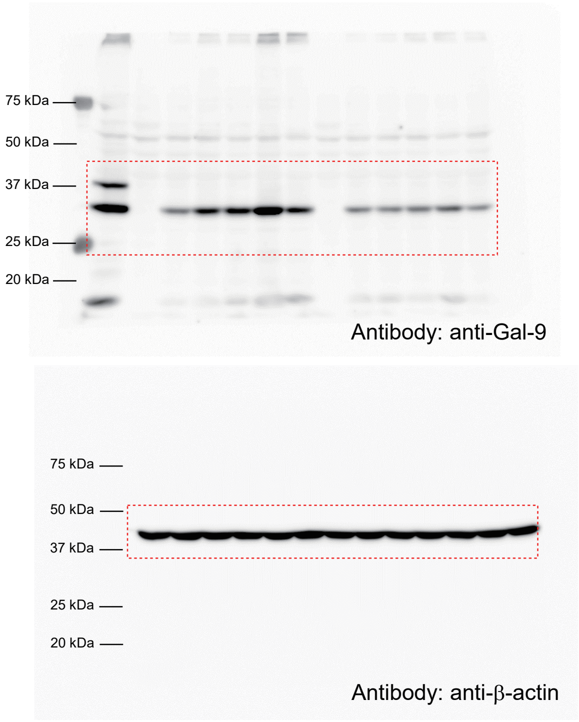

c

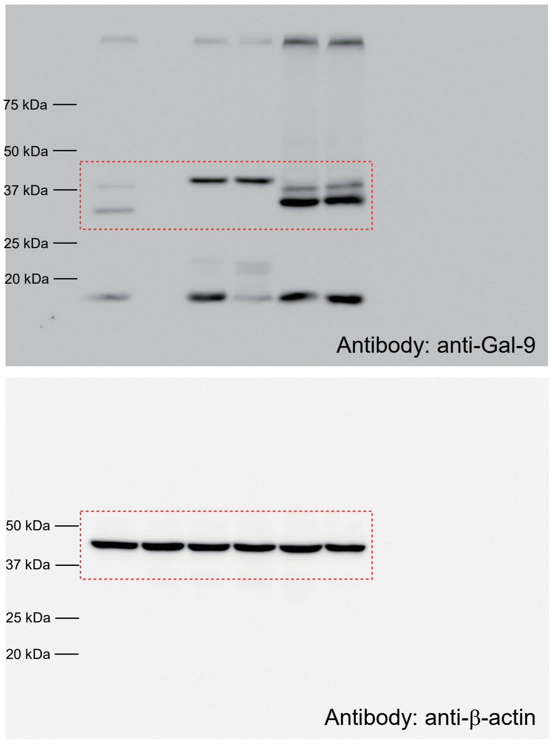

Uncropped blots for Supplementary Figure 5.

c

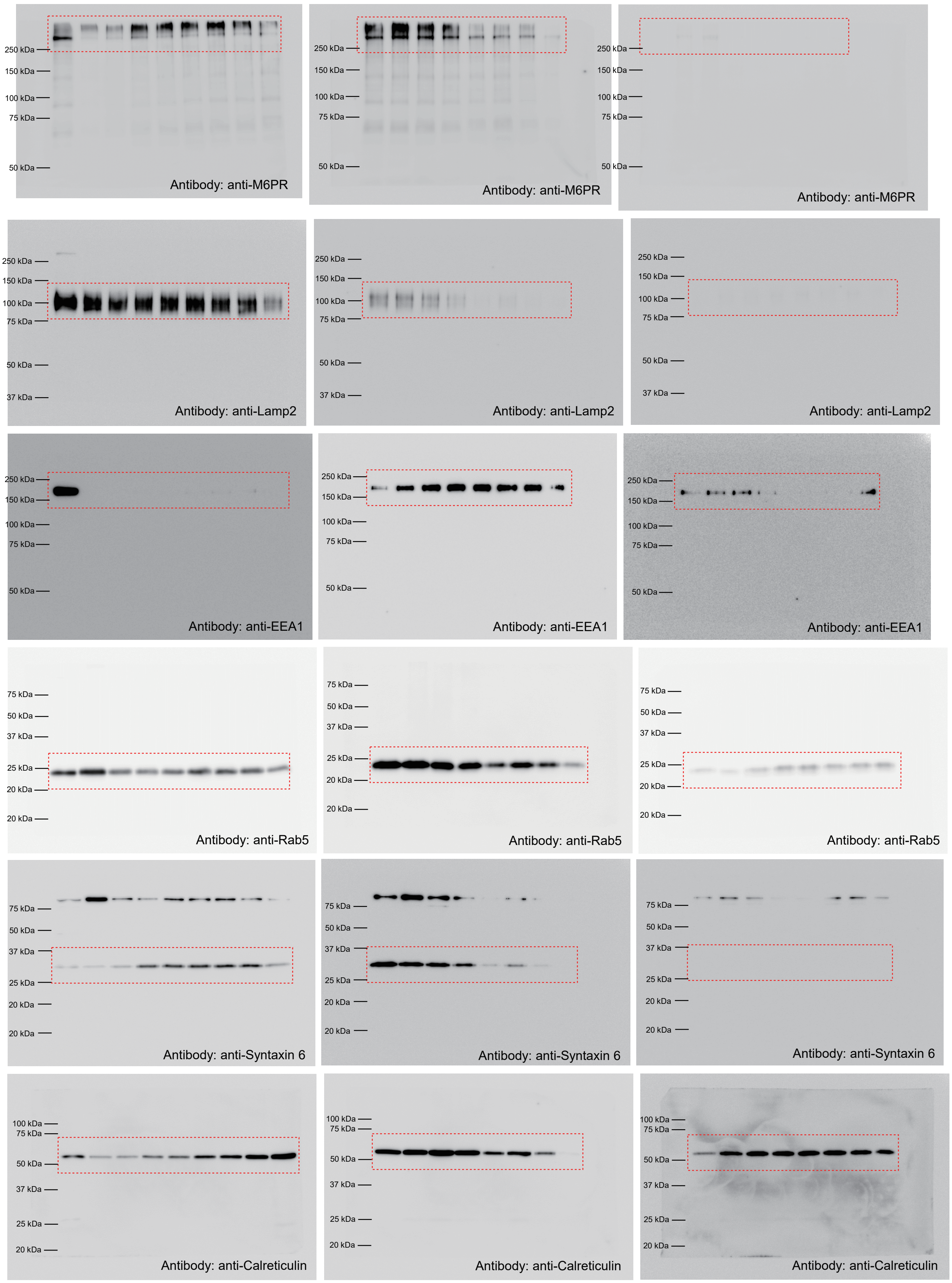

d

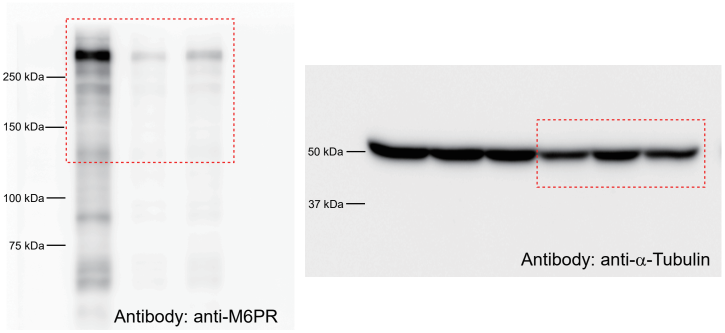

Uncropped blots for Supplementary Figure 6.

a

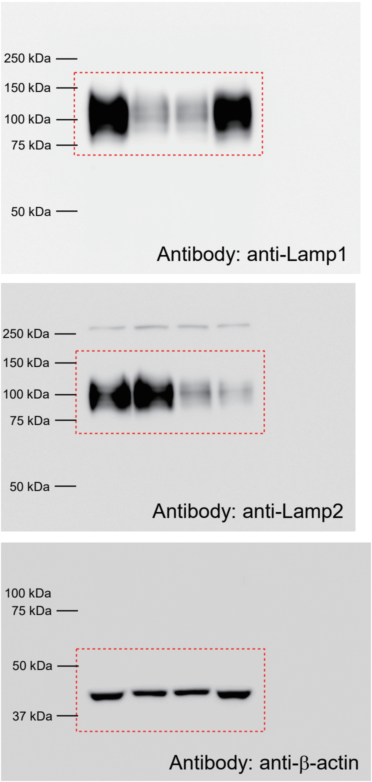

e

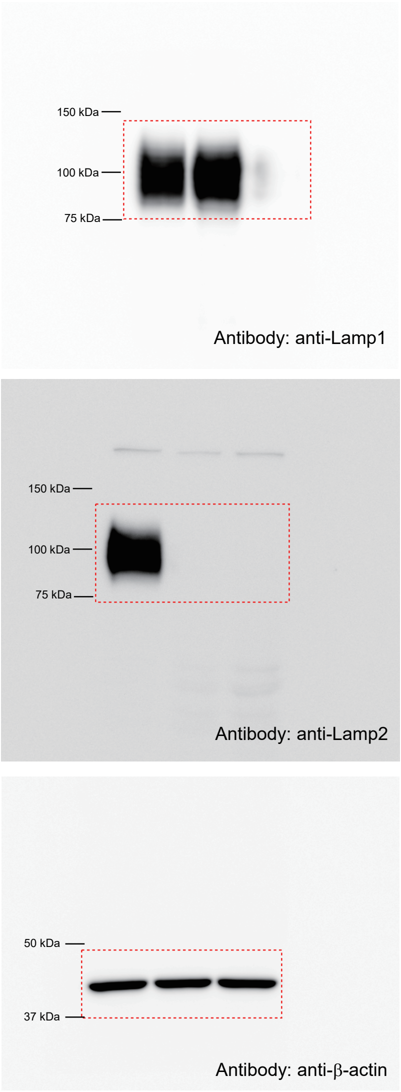

f

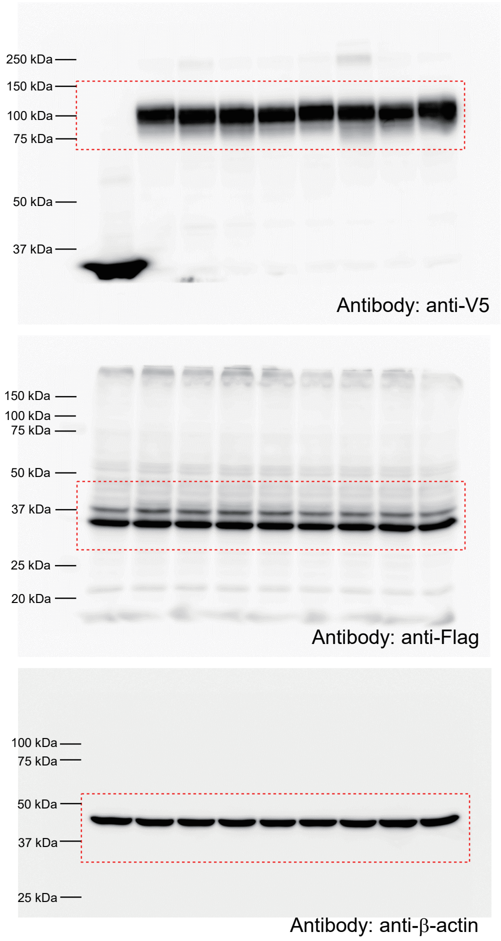

Uncropped blots for Supplementary Figure 7.

d

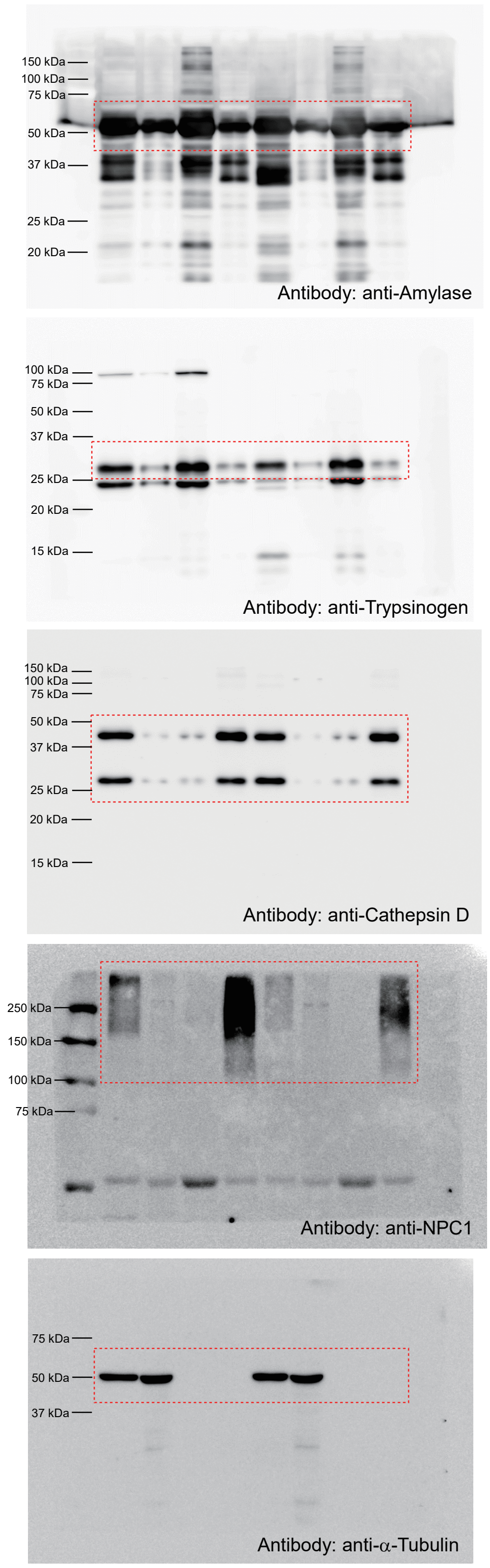

Supplement: Supplementary file 1 — Supplementary information [file 41467_2020_18102_MOESM1_ESM.pdf]
