# Supplementary material for: Lumenal Galectin-9-Lamp2 interaction regulates lysosome and autophagy to prevent pathogenesis in the intestine and pancreas
Source: Nat Commun. 2020 Aug 27;11:4286. doi: 10.1038/s41467-020-18102-7 (PMC7453023; doi:10.1038/s41467-020-18102-7)
Supplement: Supplementary file 4 — Reporting Summary [file 41467_2020_18102_MOESM4_ESM.pdf]

nature research

Corresponding author(s): Jr-Wen Shui

Last updated by author(s): Jul 10, 2020

Reporting Summary

Nature Research wishes to improve the reproducibility of the work that we publish. This form provides structure for consistency and transparency in reporting. For further information on Nature Research policies, see Authors & Referees and the Editorial Policy Checklist.

Statistics

For all statistical analyses, confirm that the following items are present in the figure legend, table legend, main text, or Methods section.

n/a	Confirmed	
☐	☒	The exact sample size (*n*) for each experimental group/condition, given as a discrete number and unit of measurement
☐	☒	A statement on whether measurements were taken from distinct samples or whether the same sample was measured repeatedly
☐	☒	The statistical test(s) used AND whether they are one- or two-sided<br>*Only common tests should be described solely by name; describe more complex techniques in the Methods section.*
☒	☐	A description of all covariates tested
☐	☒	A description of any assumptions or corrections, such as tests of normality and adjustment for multiple comparisons
☐	☒	A full description of the statistical parameters including central tendency (e.g. means) or other basic estimates (e.g. regression coefficient) AND variation (e.g. standard deviation) or associated estimates of uncertainty (e.g. confidence intervals)
☐	☒	For null hypothesis testing, the test statistic (e.g. F, t, r) with confidence intervals, effect sizes, degrees of freedom and P value noted<br>*Give P values as exact values whenever suitable.*
☒	☐	For Bayesian analysis, information on the choice of priors and Markov chain Monte Carlo settings
☒	☐	For hierarchical and complex designs, identification of the appropriate level for tests and full reporting of outcomes
☒	☐	Estimates of effect sizes (e.g. Cohen's *d*, Pearson's *r*), indicating how they were calculated

Our web collection on statistics for biologists contains articles on many of the points above.

Software and code

Policy information about availability of computer code

Data collection	BD FACS DIVA Software (v6.1.3)<br>ABI StepOne Software (v2.3)<br>Zeiss LSM700 stage - Zen 2018 (SP6 64 bit Black edition) (For Confocal Imaging)<br>FEI TECNAI G2 F20 S-TWIN (For Electron Microscopy Imaging)<br>Q Exactive™ HF Hybrid Quadrupole-Orbitrap™ Mass Spectrometer (For Mass Spectrometry)
Data analysis	Prism (GraphPad, v7.1)<br>FlowJo (v10.0.7)<br>Zen 2018 (SP6 64 bit Black edition) (For Confocal Image Analysis)<br>Microsoft Excel 2013 (For Mass Spectrometry)<br>CRISPR design tool (http://crispr.mit.edu)

For manuscripts utilizing custom algorithms or software that are central to the research but not yet described in published literature, software must be made available to editors/reviewers. We strongly encourage code deposition in a community repository (e.g. GitHub). See the Nature Research guidelines for submitting code & software for further information.

Data

Policy information about availability of data

All manuscripts must include a data availability statement. This statement should provide the following information, where applicable:

- Accession codes, unique identifiers, or web links for publicly available datasets
- A list of figures that have associated raw data
- A description of any restrictions on data availability

UniProtKB Mus musculus protein database (https://www.uniprot.org/) were used for mass spectrometry analysis. The heatmap (Fig. 4d), based on the online dataset (mouse lysosomal protein coding gene, mLGdb v.1.2, http://lysosome.unipg.it), was created. Colored Fig. 6a in this manuscript was created and partially adapted from the non-colored Fig. 1, published in https://doi.org/10.1038/s41598-018-25580-9 by Chung-Geun Lee et al. We acknowledge the original authors'

contribution and indicate their work is published under a CC BY 4.0 license. Source data and an Excel file containing raw data and analysis of mass spectrometry are provided with this manuscript. All uncropped Western blots are provided in the Supplementary information. All data presented in this study are available to the public upon request via the corresponding author.

Field-specific reporting

Please select the one below that is the best fit for your research. If you are not sure, read the appropriate sections before making your selection.

☒ Life sciences ☐ Behavioural & social sciences ☐ Ecological, evolutionary & environmental sciences

For a reference copy of the document with all sections, see nature.com/documents/nr-reporting-summary-flat.pdf

Life sciences study design

All studies must disclose on these points even when the disclosure is negative.

Sample size	Sample size for each experiment is indicated in the figure and in the Statistical analysis section. No statistical approaches were used in this study to pre-determine the sample size of experiments. We used sample size at least of three or more for each independent experiment, commonly exploited by researchers in the field. (doi:10.1016/j.immuni.2015.09.003; doi:10.1038/ni.2002; doi:10.1038/ni1271; doi:10.1038/ni.3278; doi:10.1038/nature11535)
Data exclusions	No datasets were excluded from the experiments in this study.
Replication	At least two to three independent experiments were performed and reproducible results were always obtained and calculated to achieve statistical significance.
Randomization	All cell or animal studies were randomized for group study. Age, gender-matched littermate mice were always used for animal studies.
Blinding	In general, the investigators who performed primary organoid culture or animal study were blinded for the genotypes of mice as animals were take care of and provided by other personnel in the lab. However, investigators were not blinded to the cell line study or western blot experiments as they need to arrange and load the sample based on the treatment information.

Reporting for specific materials, systems and methods

We require information from authors about some types of materials, experimental systems and methods used in many studies. Here, indicate whether each material, system or method listed is relevant to your study. If you are not sure if a list item applies to your research, read the appropriate section before selecting a response.

Materials & experimental systems

n/a	Involved in the study
☐	☒ Antibodies
☐	☒ Eukaryotic cell lines
☒	☐ Palaeontology
☐	☒ Animals and other organisms
☒	☐ Human research participants
☒	☐ Clinical data

Methods

n/a	Involved in the study
☒	☐ ChIP-seq
☐	☒ Flow cytometry
☒	☐ MRI-based neuroimaging

Antibodies

Antibodies used

rabbit anti-Amylase [aa484-511] (LSBio #LS-B11116) (IB: 1:500; IF: 1:200)
rabbit anti-ATP6V1A [EPR19270] (Abcam #199326) (IB: 1:2000)
mouse anti-b-actin [C4] (Santa Cruz Biotechnology, Inc. #47778) (IB: 1:1000)
rabbit anti-Calreticulin [EPR3924] (Abcam #ab92516) (IB: 1:5000, IF: 1:100)
rabbit anti-Syncollin [EPRR13148] (Abcam #ab178415) (IB: 1:2000)
rabbit anti-Cathepsin B [EPR21033] (abcam #ab214428) (IB: 1:1000)
rabbit anti-Cathepsin D [EPR3057Y] (abcam #ab75852) (IB: 1:2000; IF: 1:100)
rat anti-CD63 [NVG-2] (BioLegend #143902) (IF: 1:100)
rat anti-Galectin-9 [108A2] (BioLegend #137901) (IB: 1:1000; IF: 1:100)
rabbit anti-GFP [D5.1] (Cell signaling Technology #2956) (IF: 1:100)
rabbit anti-GRP78 (Abcam #ab21685) (IB: 1:3000; IF: 1:800)
rabbit anti-Ki67 [SP6] (Abcam #ab16667) (IF: 1:250)
rat anti-Lamp1 [1D4B] (Abcam #ab25245) (IB: 1:1000)
rat anti-Lamp2 [ABL-93] (Abcam #ab25339) (IB: 1:1000; IF: 1:100)
rabbit anti-Lamp2 (Thermo Fisher #PA1-655) (IF: 1:200),
rabbit anti-LC3B [D11] (Cell Signaling Technology #3868) (IB: 1:1000; IF: 1:200)
mouse anti-Lysozyme [BGN/06/961] (Abcam #ab36362) (IF: 1:100)

rabbit anti-MMP7 (Abcam #ab5706) (IF: 1:100)
mouse anti-p62 (Abcam #ab56416) (IB: 1:2000)
rabbit anti-Rab7 [D95F2] (Cell Signaling Technology #9367) (IF: 1:200)
rabbit anti-a-tubulin (Cell signaling Technology #2144), (IB: 1:1000)
mouse anti-Chop [L63F7] (Cell signaling Technology #2895), (IB: 1:1000)
rabbit anti-Cleaved Caspase-3 (Asp175) [5A1E] (Cell signaling Technology #9664), (IB: 1:1000)
rabbit anti-Xbp1 [EPR22004] (Abcam #ab220783), (IB: 1:1000)
mouse anti-Flag [M2] (Sigma-Aldrich #F1804), (IP: 5ug antibody/1mg dynabeads)
mouse anti-V5 tag [SV5-Pk1] (Abcam #ab27671), (IP: 5ug antibody/1mg dynabeads; IB: 1:1000)
goat anti-b3gnt2 (mybiosource #MBS421555), (IB: 1:1000)
rabbit anti-b4galt1 (mybiosource #MBS8242969), (IB: 1:1000)
goat anti-human galectin-9 (R&D #AF2945), (IB: 1:1000)
rabbit anti-human galectin-9 (sigma #HPA046876) (IHC: 1:100)
rabbit anti-M6PR (cation independent) [EPR6599] (Abcam #ab124767), (IB: 1:10,000)
rabbit anti-Niemann Pick C1 antibody [EPR5209] (Abcam #ab134113) (IB: 1:2000)
goat anti-CK19 (santa cruz #sc-33111) (IB: 1:1000; IHC: 1:1000)
rabbit anti-a-SMA (Abcam #ab32575) (IHC: 1:200)
rabbit anti-EEA1 (C45B10) (Cell signaling Technology #3288) (IB: 1:1000)
rabbit anti- Syntaxin 6 antibody [EP7665] (Abcam # ab140607) (IB: 1:5000)
rabbit anti-Rab5 antibody [EPR21801] (Abcam # ab218624) (IB: 1:1000)
rat anti-CD24 [M1/69] APCeFluor 780 (eBioscience #47-0242-82) (1:200 for flow cytometry)
rat anti-CD45 [30-F11] Alexa Fluor-700 (eBioscience#56-0451-82) (1:400 for flow cytometry)
rat anti-CD326 (EpCAM) [G8.8] eFluor 450 (eBioscience #48-5791-82) (1:400 for flow cytometry)
rat anti-CD44 [IM7] PECy7 (Biolegned #103029) (1:200 for flow cytometry)
rabbit anti-active Caspase-3 [C92-605] Alexa Fluor-647 (BD Biosciences #560626) (5ul/test for flow cytometry)
rat anti-Ki67 [SolA15] PeCy7 (eBioscience #25-5698-82) (1:200 for flow cytometry)
rabbit anti-Lysozyme [EC 3.2.1.17] FITC (Dako #F037201-1) (1:100 for flow cytometry)
rat anti-mouse Galectin-9 [RG9-35] APC (Biolegned #136110) (1:200 for flow cytometry)
APC/Cy7 Streptavidin (Biolegned #405208) (1:400 for flow cytometry)
anti-PNA (Vector Laboratories #FL-1071) (1:200 for flow cytometry) (5ug/ml)
DyLight 488 Labeled Lycopersicon Esculentum (Tomato) Lectin (Vector Laboratories #DL-1174-1) (5ug/ml)
anti-mouse Alexa Fluor-594 (Cell signaling Technology #8890) (IF, 1:300)
anti-rabbit Alexa Fluor-488 (Cell signaling Technology #4412) (IF, 1:300)
anti-rabbit Alexa Fluor-594 (Cell signaling Technology #8889) (IF, 1:300)
anti-rat Alexa Fluor-488 (Cell signaling Technology #4416) (IF, 1:300)
anti-rat Alexa Fluor-594 (Thermo Scientific #A-11007) (IF, 1:300)

Validation

rabbit anti-Amylase [aa484-511] (LSBio #LS-B11116)
In the manuscript: Fig. 7E, Supplementary Fig. S7A, S7D, S7F
- https://www.lsbio.com/antibodies/ihc-plus-amy2a-antibody-pancreatic-amylase-antibody-aa484-511-if-immunofluorescence-ihc-wb-western-ls-b11116/302152
-Species Reactivity: Human, Mouse
-Application: IHC, IHC-P, IF, WB

rabbit anti-ATP6V1A [EPR19270] (Abcam #199326)
In the manuscript: Fig. 3B, Fig. 7A.
-https://www.abcam.com/atp6v1a-antibody-epr19270-ab199326.html
-Species Reactivity: Mouse, Rat, Human
-Application: IHC-P, WB, ICC/IF, IP, Flow Cytometry
-Cell Death Dis 9:614 (2018)

mouse anti-b-actin [C4] (Santa Cruz Biotechnology, Inc. #47778)
In the manuscript: Fig. 1D, 1G, 2K, 3E, 3G, 4C, 5D, 5G, 6B, 6D, 6F, Supplementary S3A, S3F, Fig. S4A, S4C, S6A, S6D, S6F
-https://www.scbt.com/scbt/product/beta-actin-antibody-c4?requestFrom=search
-Species Reactivity: mouse, rat, human, avian, bovine, canine, porcine, rabbit, Dictyostelium discoideum and Physarum polycephalum
-Application: WB, IP, IF, IHC(P) and ELISA
-Nat. Chem. Biol. 14: 22-28 (2018)

rabbit anti-Calreticulin [EPR3924] (Abcam #ab92516)
In the manuscript: Supplementary Fig. S2B, S5C.
-https://www.abcam.com/calreticulin-antibody-epr3924-er-marker-ab92516.html
-Species Reactivity: Mouse, Rat, Human, Monkey
-Application: WB, IP, IHC-P, Flow Cytometry , ICC/IF
- FASEB J 26:2145-53 (2012)

rabbit anti-Syncollin [EPRR13148] (Abcam #ab178415)
In the manuscript: Fig 7A, 7B.
-https://www.abcam.com/syncollin-antibody-eprr13148-ab178415.html
-Species Reactivity: Mouse, Rat, Human
-Application: WB
- Gastroenterology 154:704-718.e10 (2018)

rabbit anti-Cathepsin B [EPR21033] (abcam #ab214428)

In the manuscript: Fig 3D.
- https://www.abcam.com/cathepsin-b-antibody-epr21033-ab214428.html
-Species Reactivity: Mouse, Rat
-Application: Flow Cyt, IHC-P, WB, ICC/IF

rabbit anti-Cathepsin D [EPR3057Y] (abcam #ab75852)
In the manuscript: Fig. 3D, Supplementary Fig. S3B, S7D.
-https://www.abcam.com/cathepsin-d-antibody-epr3057y-ab75852.html
-Species Reactivity: Mouse, Human
-Application: Flow Cytometry , ICC/IF, WB, IP, IHC-P
-PLoS One 7:e31223 (2012)

rat anti-CD63 [NVG-2] (BioLegend #143902)
In the manuscript: Supplementary Fig. S3C, S6B.
-https://www.biolegend.com/en-us/products/purified-anti-mouse-cd63-antibody-7813
-Species Reactivity: Mouse
-Application: Flow Cytometry , WB, IHC-F
-J. Immunol. 187:2268 (2011)

rat anti-Galectin-9 [108A2] (BioLegend #137901)
In manuscript: Fig. 1A, 3B, 4A, 4B, 4H, 5A, 5D, 6D, 6F, 7A, Supplementary Fig. S1B, S1C, S2I, S7A
-https://www.biolegend.com/en-us/products/purified-anti-mouse-galectin-9-antibody-6562
-Species Reactivity: Mouse
-Application: Flow Cytometry , ICFC, IHC-F, WB, ELISA.
-J Exp Med. 211:1433 (2014)

rabbit anti-GFP [D5.1] (Cell signaling Technology #2956)
In the manuscript: Fig. 2A Supplementary Fig. S1C.
-https://www.cellsignal.com/products/primary-antibodies/gfp-d5-1-xp-rabbit-mab/2956
-Species Reactivity: All
-Application: WB, IHC-P, IF, ICC, Flow Cytometry
-Nature Neuroscience volume 19, pages 1506–1512 (2016)

rabbit anti-GRP78 (Abcam #ab21685)
In the manuscript: Fig. 1G, 2K, 3G, 4C, 5G, 6F, 7F, Supplementary Fig. S2B, S6A
-https://www.abcam.com/grp78-bip-antibody-ab21685.html
-Species Reactivity: Mouse, Rat, Dog, Human, Pig, African green monkey, Chinese hamster
-Application: IHC-FoFr, IP, ICC/IF, WB, Electron Microscopy, IHC-P
-EBioMedicine 15:137-149 (2017)

rabbit anti-Ki67 [SP6] (Abcam #ab16667)
In the manuscript: Supplementary Fig S2G.
-https://www.abcam.com/ki67-antibody-sp6-ab16667.html
-Species Reactivity: Mouse, Rat, Human, Common marmoset
-Application: IHC-FoFr, ICC/IF, Flow Cytometry , IHC-Fr, WB, IHC-P
-Nat Neurosci 20:1329-1341 (2017)

rat anti-Lamp1 [1D4B] (Abcam #ab25245)
In the manuscript: Supplementary Fig. S6A, S6E.
-https://www.abcam.com/lamp1-antibody-1d4b-ab25245.html
-Species Reactivity: Mouse, Human
-Application: IHC-P, ICC/IF, Other, Flow Cytometry , IP, IHC-Fr, WB, Immunomicroscopy
- Sci Rep 7:5647 (2017)

rat anti-Lamp2 [ABL-93] (Abcam #ab25339)
In the manuscript: Fig. 1A, 1D, 2K, 3B, 4A-C, 4E, 4H, 5A, 5D, 6F, 7A, 7B Supplementary Fig. S3B-D, S5C, S6A, S6E.
-https://www.abcam.com/lamp2-antibody-abl-93-ab25339.html
-Species Reactivity: Mouse
-Application: Flow Cytometry , IHC-Fr, IP, ICC/IF, WB
-PLoS One 10:e0117412 (2015)

rabbit anti-Lamp2 (Thermo Fisher #PA1-655),
-In the manuscript: Supplementary Fig. S7A
-https://www.thermofisher.com/antibody/product/LAMP2-Antibody-Polyclonal/PA1-655
-Species Reactivity: Mouse, Rat, Human
-Application: WB, IF, ICC

rabbit anti-LC3B [D11] (Cell Signaling Technology #3868)
In the manuscript: Fig. 1D, 2K, 3B, 3E, 4C, 5G, 6F, 7B, 7E, Supplementary S3D.
-https://www.cellsignal.com/products/primary-antibodies/lc3b-d11-xp-rabbit-mab/3868
-Species Reactivity: Mouse, Rat, Human
-Application: WB, IP, IHC-P, IF, ICC, FC
-J Biol Chem. 293(18):6802-6811 (2018)

mouse anti-Lysozyme [BGN/06/961] (Abcam #ab36362)

In the manuscript: Supplementary S2J.
-https://www.abcam.com/lysozyme-antibody-bgn06961-ab36362.html
-Species Reactivity: Human/Mouse
-Application: IHC-P, WB, ELISA
-Nature Immunology 16, 918–926 (2015)

rabbit anti-MMP7 (Abcam #ab5706)
In the manuscript: Fig. 2A, Supplementary Fig. S1C.
-https://www.abcam.com/mmp7-antibody-ab5706.html
-Species Reactivity: Mouse, Human
-Application: WB, ELISA, IP, IHC-Fr, ICC/IF, IHC-P
-Carcinogenesis 29:1421-7 (2008)

mouse anti-p62 (Abcam #ab56416)
In the manuscript: Fig. 1D, 2K, 3B, 7B
-https://www.abcam.com/sqstm1--p62-antibody-ab56416.html
-Species Reactivity: Mouse, Rat, Human, Rhesus monkey, Chinese hamster
-Application: IHC-P, WB, ICC/IF, Flow Cyt, IHC-Fr
-PLoS One 8:e76187 (2013)

rabbit anti-Rab7 [D95F2] (Cell Signaling Technology #9367)
In the manuscript: Supplementary Fig S3C, S6B.
-https://www.cellsignal.com/products/primary-antibodies/rab7-d95f2-xp-rabbit-mab/9367
-Species Reactivity: Mouse, Rat, Human, Monkey
-Application: WB, IP, IF, ICC
-Sci Rep. 7:10481 (2017)

rabbit anti-a-tubulin (Cell signaling Technology #2144)
In the manuscript: Fig. 1A, 3B, 3D, 4A, 4B, 4H, 5A, 7A, 7B, 7E, 7F, Supplementary Fig S5D, S7D.
-https://www.cellsignal.com/products/primary-antibodies/a-tubulin-antibody/2144
-Species Reactivity: Mouse, Rat, Human, Monkey
-Application: WB, IHC, IF, ICC, Flow Cytometry

mouse anti-Chop [L63F7] (Cell signaling Technology #2895)
In the manuscript: Fig. 1G, 2K, 3G, 7F.
-https://www.cellsignal.com/products/primary-antibodies/chop-l63f7-mouse-mab/2895
-Species Reactivity: Mouse, Rat, Human
-Application: WB, IP, IF

rabbit anti-Cleaved Caspase-3 (Asp175) [5A1E] (Cell signaling Technology #9664)
In the manuscript: Fig. 1G, 2K, 4C, 5G, 6F.
-https://www.cellsignal.com/products/primary-antibodies/cleaved-caspase-3-asp175-5a1e-rabbit-mab/9664-Species Reactivity: Mouse, Rat, Human, Monkey
-Application: WB, IP, IHC, IF

rabbit anti-xbp1 [EPR22004] (Abcam #ab220783)
In the manuscript: Fig. 1G, 2K, 3G.
-https://www.abcam.com/xbp1-antibody-epr22004-ab220783.html
-Species Reactivity: Mouse, Human
-Application: WB, IP, Flow Cytometry

mouse anti-Flag [M2] (Sigma-Aldrich #F1804)
In the manuscript: Fig. 4E, Supplementary Fig S6F,
-https://www.sigmaaldrich.com/catalog/product/sigma/f1804?lang=en&region=TW
-Species Reactivity: Species independent
-Application: WB, IP, IF, IHC, ICC

mouse anti-V5 tag [SV5-Pk1] (Abcam #ab27671)
In the manuscript: Fig. 5D, 6D, Supplementary Fig S6F.
-https://www.abcam.com/v5-tag-antibody-sv5-pk1-ab27671.html
-Species Reactivity: Species independent
-Application: WB, IP, IF, Flow Cytometry

goat anti-b3gnt2 (mybiosource #MBS421555)
In the manuscript: Fig. 6B
-https://www.mybiosource.com/polyclonal-human-mouse-rat-antibody/b3gnt2/421555
-Species Reactivity: Mouse, Rat, Human
-Application: WB

rabbit anti-b4galt1 (mybiosource #MBS8242969)
In the manuscript: Fig. 6B
-https://www.mybiosource.com/polyclonal-bovine-human-monkey-mouse-ovine-rat-antibody/b4galt1/8242969
-Species Reactivity: Human
-Application: WB, IHC, ICC

goat anti-human galectin-9 (R&D #AF2045)
In the manuscript: Supplementary Fig. S3F
-https://www.rndsystems.com/products/human-galectin-9-antibody_af2045
-Species Reactivity: Mouse, Rat, Human
-Application: WB

Rabbit anti-human galectin-9 (sigma #HPA046876)
In the manuscript: Supplementary Fig. S1D, S1F
-https://www.sigmaaldrich.com/catalog/product/sigma/hpa046876?lang=en&region=TW
-Species Reactivity: Human
-Application: WB, IF, IHC

rabbit anti-M6PR (cation independent) [EPR6599] (Abcam #ab124767),
In the manuscript: Fig. 4E, SupplementaryFig. S5C, S5D
-https://www.abcam.com/m6pr-cation-independent-antibody-epr6599-ab124767.html
-Species Reactivity: Mouse, Rat, Human
-Application: WB, IP, ICC, IF, Flow Cytometry

rabbit anti-Niemann Pick C1 antibody [EPR5209] (Abcam #ab134113)
In the manuscript: Fig. 5A, SupplementaryFig. S7D
-https://www.abcam.com/niemann-pick-c1-antibody-epr5209-ab134113.html
-Species Reactivity: Mouse, Rat, Human
-Application: WB, ICC, IF, Flow Cytometry

goat anti-CK19 (santa cruz, #sc-33111)
In the manuscript: Fig. 7E, Supplementary Fig. S7F
-https://www.scbt.com/p/cytokeratin-19-antibody-m-17
-Species Reactivity: mouse, human
-Application: WB, IP, IHC

Rabbit anti-a-SMA (Abcam # ab32575; 1:200).
In the manuscript: Fig. 7J
-https://www.abcam.com/alpha-smooth-muscle-actin-antibody-e184-ab32575.html
-Species Reactivity: mouse, human, rat
-Application: WB, IHC, ICC

rabbit anti-EEA1 (C45B10) (Cell signaling Technology #3288)
In the manuscript: Supplementary Fig. S5C.
-https://www.cellsignal.com/products/primary-antibodies/eea1-c45b10-rabbit-mab/3288
-Species Reactivity: Mouse, Rat, Human
-Application: WB, IP, IF

Rabbit anti- Syntaxin 6 antibody [EP7665] (Abcam # ab140607).
In the manuscript: Supplementary Fig. S5C.
-https://www.abcam.com/syntaxin-6-antibody-ep7665-golgi-membrane-marker-ab140607.html
-Species Reactivity: mouse, human, rat
-Application: WB, IHC

Rabbit Anti-Rab5 antibody [EPR21801] (Abcam # ab218624).
In the manuscript: Supplementary Fig. S5C.
-https://www.abcam.com/rab5-antibody-epr21801-ab218624.html
-Species Reactivity: mouse, human, rat
-Application: WB, IHC, IP, ICC

rat anti-CD24 [M1/69] APCeFluor 780 (eBioscience #47-0242-82)
In the manuscript: Fig. 1C, 2D, 2G, Supplementary Fig. S2D, S2G-I.
- https://www.thermofisher.com/antibody/product/CD24-Antibody-clone-M1-69-Monoclonal/47-0242-82
-Species Reactivity: Mouse
-Application: Flow cytometry
-Am J Physiol Gastrointest Liver Physiol.300(3):G409-17., 2011

rat anti-CD45 [30-F11] Alexa Fluor-700 (eBioscience#56-0451-82) (1:400 for flow cytometry)
In the manuscript: Fig. 1C, 2D, 2G, 7C, Supplementary Fig. S2D, S2G-I, S7E.
- https://www.thermofisher.com/antibody/product/CD45-Antibody-clone-30-F11-Monoclonal/56-0451-82
-Species Reactivity: Mouse
-Application: Flow cytometry
-Am J Physiol Gastrointest Liver Physiol.300(3):G409-17., 2011

rat anti-CD326 (EpCAM) [G8.8] eFluor 450 (eBioscience #48-5791-82) (1:400 for flow cytometry)
In the manuscript: Fig. 1C, 2D, 2G, Supplementary Fig. S2D, S2G-I.
-https://www.thermofisher.com/antibody/product/CD326-EpCAM-Antibody-clone-G8-8-Monoclonal/48-5791-82
-Species Reactivity: Mouse
-Application: Flow cytometry
-Am J Physiol Gastrointest Liver Physiol.300(3):G409-17., 2011

rat anti-CD44 [IM7] PECy7 (Biolegned #103029) (1:200 for flow cytometry)
In the manuscript: Fig. 1C, 2D, 2G, Supplementary Fig. S2D, S2G-I.
-https://www.biolegend.com/fr-ch/products/pe-cy7-anti-mouse-human-cd44-antibody-3932
-Species Reactivity: Mouse, Human
-Application: Flow cytometry
-GASTROENTEROLOGY 145(2):383–395., 2013

rabbit anti-active Caspase-3 [C92-605] Alexa Fluor-647 (BD Biosciences #560626) (5ul/test for flow cytometry)
In the manuscript: Fig. 3I, Supplementary Fig.S2D, S7E
-https://www.bdbiosciences.com/us/applications/research/intracellular-flow/intracellular-antibodies-and-isotype-controls/anti-human-antibodies/alexa-fluor-647-rabbit-anti-active-caspase-3-c92-605/p/560626
-Species Reactivity: Mouse, Human
-Application: Flow cytometry

rat anti-Ki67 [SolA15] PeCy7 (eBioscience #25-5698-82) (1:200 for flow cytometry)
In the manuscript: Fig. 2G, Supplementary Fig.S2G
- https://www.thermofisher.com/antibody/product/Ki-67-Antibody-clone-SolA15-Monoclonal/25-5698-82
-Species Reactivity: Dog, Cynomolgus Monkey, Human, Mouse, Non-human primate, Rat
-Application: Flow cytometry

rabbit anti-Lysozyme [EC 3.2.1.17] FITC (Dako, Now part of Agilent #F037201-1) (1:100 for flow cytometry)
In the manuscript: Fig. 2D, 2G, Supplementary Fig.S2I
- https://www.agilent.com/store/en_US/Prod-F037201-1/F037201-1
-Species Reactivity: Human
-Application: Flow cytometry
-Am J Physiol Gastrointest Liver Physiol.300 (3):G409-17., 2011

rat anti-mouse Galectin-9 [RG9-35] APC (Biolegned #136110)
In the manuscript: Fig. 2D
-https://www.biolegend.com/ja-jp/products/apc-anti-mouse-galectin-9-antibody-8239
-Species Reactivity: mouse
-Application: Flow cytometry

APC/Cy7 Streptavidin (Biolegned #405208)
In the manuscript: Fig. 7C.
-https://www.biolegend.com/ja-jp/products/apc-cyanine7-streptavidin-1471
-Application: Flow cytometry, IF

anti-PNA (Vector Laboratories #FL-1071)
In the manuscript: Fig. 7C.
-https://vectorlabs.com/fluorescein-labeled-peanut-agglutinin-pna.html
-Application: Flow cytometry, IF
-Mol Reprod Dev, 75 (5), 699-706, 2008

DyLight 488 Labeled Lycopersicon Esculentum (Tomato) Lectin (Vector Laboratories #DL-1174-1)
In the manuscript: Fig. 6C, Supplementary Fig.S6G
-https://vectorlabs.com/dylight-488-labeled-lycopersicon-esculentum-tomato-lectin-lel-tl.html
-Application: Flow cytometry, IF
-Sci Rep, 6, 21127, 2016

anti-mouse Alexa Fluor-594 (Cell signaling Technology #8890) (IF, 1:300)
In the manuscript: Supplementary Fig.S2I, S2J
-https://www.cellsignal.com/products/secondary-antibodies/anti-mouse-igg-h-l-f-ab-2-fragment-alexa-fluor-594-conjugate/8890
-Application: Flow cytometry, IF

anti-rabbit Alexa Fluor-488 (Cell signaling Technology #4412) (IF, 1:300)
In the manuscript: Fig. 2A, Supplementary Fig.S1C, S3B, S3C, S3D, S6B
-https://www.cellsignal.com/products/secondary-antibodies/anti-mouse-igg-h-l-f-ab-2-fragment-alexa-fluor-594-conjugate/8890
-Application: Flow cytometry, IF

anti-rabbit Alexa Fluor-594 (Cell signaling Technology #8889) (IF, 1:300)
In the manuscript: Fig. 2A, Supplementary Fig.S1C, S2B, S2G, S7A
-https://www.cellsignal.com/products/secondary-antibodies/anti-rabbit-igg-h-l-f-ab-2-fragment-alexa-fluor-594-conjugate/8889
-Application: Flow cytometry, IF

anti-rat Alexa Fluor-488 (Cell signaling Technology #4416) (IF, 1:300)
In the manuscript: Supplementary Fig.S1B, S1C, S2I
-https://www.cellsignal.com/products/secondary-antibodies/anti-rat-igg-h-l-alexa-fluor-488-conjugate/4416
-Application: Flow cytometry, IF

anti-rat Alexa Fluor-594 (Thermo Scientific #A-11007) (IF, 1:300)
In the manuscript: Supplementary Fig.S1C, S3B, S3C, S3D, S6B, S7A
-https://www.thermofisher.com/antibody/product/Goat-anti-Rat-IgG-H-L-Cross-Adsorbed-Secondary-Antibody-Polyclonal/A-11007

-Application: Flow cytometry, IF

Eukaryotic cell lines

Policy information about cell lines

Cell line source(s)	Mouse colon epithelial cell lines CMT-93 were purchased from ATCC (Cat# ATCC® CCL-223™).<br>Human epithelial, adherent colorectal adenocarcinoma cell line HT-29 (ATCC® HTB-38™)<br>Human epithelial, adherent embryonic kidney cell line HEK-293 (ATCC® CRL-1573™)
Authentication	All cell lines were directly obtained from ATCC with a certificate attached. And, ATCC authenticates cell lines routinely with the following tests: Cellular Morphology, Karyotyping, Cytochrome C Oxidase I (COI) Assay Testing.
Mycoplasma contamination	All cell lines or derived mutant cell lines were free of mycoplasma contamination determined by the nucleus staining or PCR amplification.
Commonly misidentified lines (See ICLAC register)	No commonly misidentified cell lines were used in the study.

Animals and other organisms

Policy information about studies involving animals; ARRIVE guidelines recommended for reporting animal research

Laboratory animals	Galectin-9-/- mice were obtained from Dr. Liu, Fu-Tong, Institute of Biomedical Sciences, Academia Sinica, Taiwan. Galectin-9-/- mice were in C57BL/6 background (https://doi.org/10.1016/j.ajpath.2018.01.017) and we have further backcrossed Galectin-9-/- mice to C57BL/6 mice for two more generations before intercrossing them to have littermates for our experiments. Lgr5-eGFP-ires-creERT2 mice were obtained from Jackson Laboratory. Lgr5-eGFP mice were bred with Galectin-9-/- mice to obtain Lgr5-eGFP+Galectin-9-/- mice. Galectin-9 conditional knockout (Galectin-9flox/flox) mice in C57BL/6 background were generated via Transgenic Mouse Core Facility in National Taiwan University by the CRISPR/Cas9. To minimize off-target effects in CRISPR mice, Galectin-9flox/flox mice were first backcrossed to C57BL/6 mice for four generations before breeding into Paneth cell-specific Galectin-9 conditional knockout mice (Defa6-Cre+Galectin-9flox/flox). The Defa6-Cre mice were obtained from Dr. Richard S. Blumberg, Division of Gastroenterology, Department of Medicine, Brigham and Women's Hospital, Harvard Medical School. Animals were maintained in a specific-pathogen-free (SPF) facility at a relative humidity 50±10%, 20-26 °C, and in 12 h dark/light cycles (08:00–20:00 light). Experiments were performed on mature animals (age, 8 to 12 wk) in both male and female mice, unless otherwise indicated. Animal care and experimental protocols (Protocol ID: 15-12-908) have been approved by the Institutional Animal Care and Use Committee (IACUC) at the Institute of Biomedical Sciences, Academia Sinica. Ethical compliance has been observed in animal study. Dr. John T. Kung is the chair of IACUC and Ethics Committee in Academia Sinica.
Wild animals	The study here did not use any wild animals.
Field-collected samples	The study here did not use any samples collected from the field.
Ethics oversight	Animal care and experimental protocols (Protocol ID: 15-12-908) have been approved by the Institutional Animal Care and Use Committee (IACUC) at the Institute of Biomedical Sciences, Academia Sinica. Ethical compliance has been observed in animal study. Dr. John T. Kung is the chair of IACUC and Ethics Committee in Academia Sinica.

Note that full information on the approval of the study protocol must also be provided in the manuscript.

Flow Cytometry

Plots

Confirm that:

☒ The axis labels state the marker and fluorochrome used (e.g. CD4-FITC).

☒ The axis scales are clearly visible. Include numbers along axes only for bottom left plot of group (a 'group' is an analysis of identical markers).

☒ All plots are contour plots with outliers or pseudocolor plots.

☒ A numerical value for number of cells or percentage (with statistics) is provided.

Methodology

Sample preparation	The biological sources of the primary cells were isolated from intestine or pancreas of WT and Gal-9 KO mice as described in the Method section. Mouse colon epithelial cell lines CMT-93 were purchased from ATCC (Cat# ATCC® CCL-223™).Human epithelial, adherent colorectal adenocarcinoma cell line HT-29 (ATCC® HTB-38™).
Instrument	BD LSR II flow cytometry
Software	BD FACS DIVA Software (v6.1.3) were used to collect data. FlowJo (v10.0.7) software were used to analyze data. No custom code has been deposited into a community repository.

Cell population abundance	The purity of the intestinal epithelial cells were determined by the labeling of EpCAM, a marker that is specific to epithelial cells, and CD45, a specific marker for leukocyte that do not express by epithelial cells. The purity of the WT and Gal-9 KO epithelial cells were 86.20 ± 0.79% and 86.94 ± 3.94%, respectively.<br>The purity of pancreatic acinar cells were determined by the labeling of PNA, a marker specific to pancreatic acinar cells and CD45, a specific marker for leukocyte that do not express by pancreatic acinar cells. The purity of the WT and Gal-9 KO acinar cells were 80.07 ± 2.85% and 79.17 ± 0.15%, respectively.
Gating strategy	In epithelial samples gating, samples were first gated to distinguish populations of cells based on their side and forward scatter properties (SSC-A vs FSC-A), followed by singlets (FSC-H vs. FSC-A). To define epithelial population, the samples were gated on CD45- EpCAM+ cells. For Lysozyme, MDC, AO, Lysotracker, Caspase-3 staining, CD44, a surface marker expressed by intestinal crypts was included to enrich the population.<br><br>In pancreatic samples gating, samples were first gated to distinguish populations of cells based on their side and forward scatter properties (SSC-A vs FSC-A), followed by singlets (FSC-H vs. FSC-A). To define acinar cell population, the samples were gated on CD45- PNA+ cells.

☒ Tick this box to confirm that a figure exemplifying the gating strategy is provided in the Supplementary Information.
