# Supplementary material for: Lumenal Galectin-9-Lamp2 interaction regulates lysosome and autophagy to prevent pathogenesis in the intestine and pancreas
Source: Nat Commun. 2020 Aug 27;11:4286. doi: 10.1038/s41467-020-18102-7 (PMC7453023; doi:10.1038/s41467-020-18102-7)
Supplement: Supplementary file 2 — Peer Review File [file 41467_2020_18102_MOESM2_ESM.docx]

Peer Review Information

**Journal:** Nature Communications

**Manuscript title:** Lumenal Galectin-9-Lamp2 interaction regulates lysosome and autophagy to prevent pathogenesis in the intestine and pancreas

**Corresponding author name(s):** Dr Jr-Wen Shui

**Editorial notes:**

**none**

**Reviewer comments & decisions:**

**Reviewer comments, first version:**

Reviewer #1 (Remarks to the Author):

This study investigates interaction between galectin 9 (Gal-9), a galactoside-binding lectin, and the major lysosomal membrane protein LAMP2, and the roles of Gal-9 in lysosomal stability and cellular homeostasis in the exocrine pancreas and intestine. Galectins were previously shown to modulate the activity of glycoproteins on both cell surface and intracellular compartments, Gal-9 regulates immune responses; however, the effects of Gal-9 on lysosomes in pancreas and intestine have not been examined. Recent studies showed that lysosomal dysfunction (caused, in particular, by LAMP2 decrease) leads to pancreatitis. Therefore, understanding of the mechanisms mediating lysosomal function, particularly LAMP2 stability, in pancreas is clinically relevant. However, the study has serious drawbacks both in experimental design and data interpretation, and many conclusions are not substantiated by experimental data.

Major comments:
1. The authors claim (Figs. 1 and S1) that Gal-9 regulates lysosomal stability and function in pancreatic acinar cells but present no experimental evidence other than a decrease in acridine orange (AO) staining in Gal-9 null cells. Zymogen granules (ZG) in these cells have acidic pH (Niderau C, et al. Gastroenterology, 1986:91:1433-42), so changes in AO intensity (Fig. 1E) could due to pH changes in not only lysosomes but also ZG. This can be assessed by co-localizing AO with markers of both lysosomes and ZG. To evaluate lysosomal functions, the authors should measure the effect of Gal-9 deficiency on activities of lysosomal hydrolases, levels of lysosomal membrane proteins (in addition to LAMP2), and on autophagic flux in acinar cells.
2. Different from their data in the intestine, the authors’ conclusion that Gal-9 interacts with LAMP2 in acinar cells lacks sufficient evidence. IF (Figs 1A, S1A) shows Gal-9 colocalization with ZG (amylase) and ER (GRP78) but, amazingly, there is no data indicating Gal-9 presence in acinar cell lysosomes.
3. A major weakness of the study is the use of general, not organ-specific, Gal-9 KO. Gal-9 is present in the immune/inflammatory cells and modifies their activity. Thus, it is impossible to make any mechanistic conclusion on the effect of Gal-9 knockout on inflammation, a major pathologic response of pancreatitis – both local (pancreatic) and systemic.
4. The authors’ conclusion that Gal-9 KO mice develop spontaneous pancreatitis (p. 9) is based on only 2 parameters of pancreatitis, fibrosis and apoptosis. To make this conclusion solid, the authors should also measure hallmark responses of pancreatitis, such as changes in pancreatic levels of digestive enzymes, trypsinogen activation, acinar/ductal metaplasia.
5. The authors conclude in the Abstract that Gal-9 deficiency “impaired homeostatic cargo degradation,” which is not substantiated by specific measurements of autophagic flux in Gal-9 null vs. WT acinar cells.
6. Changes in IF intensity of LAMP2, LC3 and p62 (Fig. 1B) should be quantified and corroborated by measurements of LAMP2, LC3, and p62 levels with western blot.
7. Fig. 2 discusses the effect of Gal-9 deficiency on acute pancreatitis induced by cerulein (CER-AP); saline-injected Gal-9 KO mice serve as controls. Surprisingly, the data in saline-treated Gal-9 KO vs WT mice show no signs of pancreatitis – contradicting the effects of Gal-9 deficiency on pancreas shown in Fig. 1 (on which the conclusion about spontaneous pancreatitis was based). For example, in Fig. 1 Gal-9 deficiency increased LC3 dots (Fig. 1B), caused edema (Fig. 1K) and acinar cell death (Fig. 1J); but the data in Fig. 2G show no difference in edema, vacuolization, and cell death between saline-treated KO and WT pancreas (!). A possible explanation is that saline/CER injections were performed on much younger mice, but this should be examined. Anyway, the above discrepancies should be reconciled.

I also have serious concerns RE the methods used:
1. Make sure to specify the genetic background of Gal-9 KO mice.
2. The authors performed in vitro experiments using 24-h or 48-h acinar cell culture. Of note, isolated mouse or rat acinar cells undergo time dependent de-differentiation, losing acinar and acquiring ductal cell phenotype (Sphyris N et al. Pancreas 2005; 30(2):148-57); loss of endolysosomal markers, such as Rab5, EEA1 and LAMP1, have been described after 6-h of culture (Messenger S et al. Am J Physiol Gastrointest Liver Physiol 2013;305:G439–G452). Thus, prolonged culture of acinar cells requires caution, various controls, and should be documented in great detail (e.g., Mareninova OA et al. J Clin Invest 2009;119:3340-3355). To validate the in vitro/ex-vivo system they use, the authors should assess changes in the levels of amylase, the ductal cell marker CK19, and the above endolysosomal markers; and functional parameters such as hormone-induced amylase secretion or trypsin activity. But the obvious question is: why not do the measurements on freshly isolated acinar cells, i.e., within the “standard” time frame of 6-h incubation (of course, experiments on transduced cells require prolonged culture).
3. The authors extensively use FACS analysis of acinar cells. This is, at best, problematic, as the standard pancreas digestion with collagenase yields a suspension of pancreatic acini, groups containing different numbers (from 3 to 20) of acinar cells. Moreover, the average size of isolated acini strongly depends on “hands” (and varies from one preparation to another even in the same hands). Thus, flow cytometry data on acinar cells are not reliable.
4. Many experiments in the paper include treatment of mice with tunicamycin, an inhibitor of endogenous N-glycosylation. LAMP-2 is a highly N-glycosylated protein; thus, the authors should check LAMP2 glycosylation status in these experiments.
5. The authors measure trypsinogen activation with an enzymatic assay using Rhodamine 110, which can be cleaved by various proteases. Trypsin activity data should be re-measured using a selective trypsin substrate Boc-Gln-Ala-Arg-AMC, as done in numerous publications.


Reviewer #2 (Remarks to the Author):

This manuscript provides evidence that luminal galectin-9 promotes lysosome stabilization and that this is mediated via an interaction with Lamp2. This is an interesting idea, not least because it is conceptually opposite to the previously identified role of cytosolic galectins in lysosome homeostasis, in which they bind to exposed glycans when the limiting membrane of the lysosome is damaged, resulting in lysophagy. As the authors explain in their introduction, the specific role of galectins-3 and -8 in the molecular mechanism of lysophagy has previously been characterized. The starting point of the present manuscript was to address the question of whether Gal-9 has a function in the homeostasis of autophagy-active acinar cells in the pancreas. Using a mouse model (cerulein treatment) of acute pancreatitis, as well as acinar cells or tissue from Gal-9 -/- mice, the authors provide compelling evidence that Gal-9 is essential for maintaining lysosomal function and autophagy in pancreatic acinar cells. A key experiment shown in Fig 1E was to test lysosome stability by ‘photo-oxidation stimulation of acridine orange -labelled acinar cells to induce lysosome membrane permeabilization (LMP)’. Gal-9 -/- cells showed increased susceptibility to LMP challenge. The authors also studied the effects of tunicamycin treatment, which inhibits N-glycosylation and has previously been shown to cause ER stress and LMP, finding that tunicamycin treated Gal-9 -/- mouse cells showed increased autophagy blockade and greater ER stress. In addition to the characterization of acinar cells shown in Fig 1, the authors went on to provide data in Fig 2 that support their conclusion that cerulein-mediated induction of deleterious zymogens and subsequent necrotic pancreatitis requires Gal-9-regulated autophagy. The authors then turned their attention to crypt cells in the gut, in which they showed (Fig 3) that Gal-9 was predominantly expressed and found a similar requirement for Gal-9 in maintaining lysosomal stability and affecting ER-stress. Importantly, they were able to reproduce their findings in organoid cultures, a system in which they were able to do rescue experiments, showing that exogenous recombinant Gal-9 could rescue the effects of Gal-9 knockout. In Fig 4 the authors provide evidence that Gal-9 regulates crypt homeostasis, anti-microbial immunity and inflammation. This is followed by a series of experiments (Figs 5 and 6) in CMT93 cells ± Gal-9 CRISPR knockout, in which the core findings from the acinar cells and intestinal crypts were reproduced and which address the central question of how luminal Gal-9 promotes lysosome stabilization. They obtained evidence that endogenous Gal-9 co-fractionates with LAMP-positive organelles and showed in Gal-9 knockout cells that they could rescue LMP and acidification defects with exogenous recombinant Gal-9, not Gal-3. They then reconstituted the Gal-9 knockout cells with wild-type or mutant Gal-9 to demonstrate the importance of the glycan-binding function of Gal-9 for rescue. Pull down of FLAG-tagged Gal-9 found LAMP1 and LAMP2 and the binding to LAMP2 was confirmed by co-immunoprecipitation. The authors finally propose a model in which luminal Gal-9 strengthens or expands the glycocalyx, although no direct evidence is presented.
Overall, this is a manuscript with an interesting central proposal and experiments that support it. However, it needs some additional experiments and a major re-write before it can be published.

Major points
1. A major caveat to this review is that all information about the Gal-9 -/- mice in Materials and Methods was removed due to double-blind peer review process. Therefore, I have simply assumed that that they are indeed Gal-9 -/- and have been appropriately genotyped and phenotyped.
2. It has previously been suggested that Gal-9 senses damaged lysosomes, leading to lysophagy (see e.g. Papadopoulos C et al (2019) J Mol Biol. 2019 Aug 23. pii: S0022-2836(19)30519-4). In their introduction, the authors quote reference 13 with respect to Gal-8 initiating anti-bacterial autophagy but should also point out that the same paper states that “Damage to lysosomes by glycyl-L-phenylalanine 2-naphthylamide (GPN) treatment resulted in the initial loss of lysotracker staining, followed by the appearance of galectin 3, 8 and 9 speckles “
3. The authors use tunicamycin treatment as a model of LMP (they write “LMP challenge such as tunicamycin”). However, they have not themselves shown that it causes LMP in their cell types, nor have they shown that the ER stress that results is a consequence of LMP rather than being a direct effect at the level of the ER (as has been shown by many other groups in different cell types), which seems much more likely.
4. Fig S3, Panels B,C, D (which needs quantification of colocalization) and G should be in main figures, not supplementary since they address the central proposition of the paper, which is that Gal-9-LAMP2 interaction promotes lysosome stabilization.
5. The data in Fig 4 showing that Gal-9 regulates crypt homeostasis, anti-microbial immunity and inflammation is not central to the main message of the manuscript that luminal Gal-9 promotes lysosome stabilization and would be better shown as supplementary information.
6. The mass spec data for the pull down of FLAG-tagged Gal-9 must be shown.
I do not understand why the authors only looked at the interaction of Gal-9 with LAMP2. They state that LAMP2 is the most abundant LAMP , but they provide no evidence this is the case in the cells they are studying. It is certainly not true in HeLa cells, where mass spectrometry has shown ~4x more LAMP1 than LAMP2 (URL: http://mapofthecell.org).
7. The authors propose a model in which luminal Gal-9 strengthens or expands the glycocalyx, but wouldn’t a simpler model be that by binding to the glycocalyx it simply acts to block access of enzymes that hydrolyse glycans and thereby extends the half-life of the LAMPs ?
Minor Points
1. P4- description of Fig 1A . What evidence is there of rapid induction of amylase+ acini after cerulein injection ? Only one time point is measured, at 8 h after the first injection and I h after the last.
2. P4. The PCR analysis described here measures mRNA – it cannot directly show that ’cerulein induces rapid release of zymogens’ as stated.
3. P4. In contrast to the text statement, Fig 2G. shows that cerulein does greatly increase vacuolization and necrosis in Gal9-/- mice, just not as much as in wild type mice. The current sentence implying no vacuolization and necrosis in Gal9-/- mice is incorrect.
4. Fig S3B. The authors need to explain why they see no increase in LC3 in the Gal-9 -/- CMT-93 cells compared to WT cells.
5. Fig 5A. There needs to be a blot of the PNS loaded onto the step gradient, not just the gradient fractions.
6. Fig6D. The EGF degradation experiment in Fig 6D must be quantified.
7. In Figure legends it is stated that error bars indicate ‘means±SEM’ from ‘at least 2 independent experiments’. Instances where there are only 2 independent experiments should be identified and ‘means±range’ shown. Presumably none of the p values relate to situations where there are only 2 experiments.
8. P25. The section on reconstitution of Gal-9 KO CMT93 cells is incomplete. There is no explanation of how the cells are reconstituted. Somewhere there also needs to be data showing, or a statement saying, that cells took up/expressed equal amounts of recombinant Gal-9 in experiments where WT and mutants were being compared.
9. The end of the Results section contains speculation about how Gal-9 may be functioning that would more appropriately be placed in the Discussion.


Reviewer #3 (Remarks to the Author):

Galectin-9 is a widely expressed galectin that is known to bind certain features of mammalian glycans, in particular poly-N-acetyllactosamine-type sequences of repeating Gal-GlcNAc. These are glycan features known to occur on lysosome-associated membrane proteins 1/2 (Lamp1/2d). Here the authors identify a functional role of galectin-9 binding to Lamp2 in that regard which is interpreted to be important in lysosomal stabilization, ER stress/homeostasis, and other cellular pathways. The functions of galectins in general are widespread, as the proteins occur both in the cytoplasm, intracellularly within organelles (here within lysosomes), and on the cell surface and in secretions. Here the authors explore a key potential function of galectin-9, a secreted lectin that may acquire access to the lysosome via secretion and uptake into lysosomes via interactions with Lamp2. The study relates to the process of autophagy, or self-degradation, whereby cellular material is degraded in survival responses, with an emphasis on pancreatic disorders and intestinal Paneth cells.. The study is exciting in many ways and provides novel insights into both galectin-9 functions, lysosomal glycoproteins, and roles of autophagy in lysosomal function and pathogenesis. There are several questions the authors could address that could strengthen the impact of the study.

1. It is fundamentally not clear how galectin-9 binding to lamp1/2 and present in the lysosome in some way stabilizes the glycoproteins? What would that mechanisms be, and would it involve cross-linking, precipitation, or some other mechanism? Is the galectin-9 also stabilized there by its interactions with lamp1/2? This is an especially important issues, as the authors indicate, prior studies had concluded it was the glycosylation of the glycoproteins that was required for their stabilization, but here the authors indicate that interactions with galectin-9 are required.
2. How does galectin-9 gain access to the lysosomal compartment in normal cells? Is it through endocytosis involving lamp1/2 or some other surface glycoproteins? The authors do not demonstrate mechanistically that lamp1/2 at the cell membrane are used in uptake of galectin-9, should comment on the possibility that membrane glycosphingolipids, which can also contain poly-N-acetyllactosamine moieties, might be important.
3. A key focal point of the paper is galectin-9 null mice, which is a general, not targeted, deletion of galectin-9. This raises the question of the origin of galectin-9 in lysosomes, is it provided by lymphocytes, which also have a lot of the protein or is it provided by the acinar cells?
4. Galectin-9 is presumed to interact with the long chain poly-N-acetyllactosamine moieties as the authors indicate. If the synthesis of such chains is blocked, without affecting overall N-glycan biosynthesis, are the contributions of galectin-9 muted?
5. The authors refer to intracellular functions, but for this reviewer the authors should make a clear distinction between cytoplasmic localization, where galectin-9 and presumably all galectins are generated on cytoplasmic ribosomes, versus intraorganelle presence in the cytoplasm as in lysosomes.
6. A prior study by Itoh et al (2019), not referenced herein, showed that galectin-9 in prostate cancer cells can induce apoptosis by entry into lysosomes and presumed association with ligands there.
7. Galectin-9 is a tandem-repeat galectin, and susceptible to cleavage by thrombin, depending on the isoforms. The authors should identify the isoforms (L or S) here in their studies in pancreatic acinar cells, and whether the galectin-9 within lysosomes is susceptible to proteolysis there.
8. In the tissues and cells examined in this study there are probably other galectin family members expressed, which may also bind poly-N-acetyllactosamine moieties and other N-glycans. The authors should provide information about the presence of other galectins if possible because of potential compensatory or conflicting activities of these other proteins.

**Author rebuttal, first version:**

**Reviewer-1:** (Remarks to the Author)

This study investigates interaction between galectin-9 (Gal9), a galactoside-binding lectin, and the major lysosomal membrane protein LAMP2, and the roles of Gal9 in lysosomal stability and cellular homeostasis in the exocrine pancreas and intestine. Galectins were previously shown to modulate the activity of glycoproteins on both cell surface and intracellular compartments, Gal9 regulates immune responses; however, the effects of Gal9 on lysosomes in pancreas and intestine have not been examined. Recent studies showed that lysosomal dysfunction (caused, in particular, by LAMP2 decrease) leads to pancreatitis. Therefore, understanding of the mechanisms mediating lysosomal function, particularly LAMP2 stability, in pancreas is clinically relevant. However, the study has serious drawbacks both in experimental design and data interpretation, and many conclusions are not substantiated by experimental data.

Major comments:

1. The authors claim (Figs. 1 and S1) that Gal9 regulates lysosomal stability and function in pancreatic acinar cells but present no experimental evidence other than a decrease in acridine orange (AO) staining in Gal9 null cells. Zymogen granules (ZG) in these cells have acidic pH (Niderau C, Gastroenterology, 1986:91:143342), so changes in AO intensity (Fig. 1E) could due to pH changes in not only lysosomes but also ZG. This can be assessed by colocalizing AO with markers of both lysosomes and ZG. To evaluate lysosomal functions, the authors should measure the effect of Gal9 deficiency on activities of lysosomal hydrolases, levels of lysosomal membrane proteins (in addition to LAMP2), and on autophagic flux in acinar cells.

**Our response:**

To address this, we have first tested colocalization of AO/LysoTracker dye with Lamp2 (lysosome marker) and Syncollin (ZG marker) by immunofluorescence. Unfortunately, surface antibody staining is technically incompatible with AO/LysoTracker dye which only works with living cells. We then isolated lysosome and ZG fractionations from fresh acinar cells for AO/LysoTracker staining, which was similar to the approach used by Gastroenterology 91:1433, 1986 as reviewer indicated. Our cell fractionation protocol using fresh acinar cells worked very well (revised **Fig.7A**, **S6D**), as lysosome fraction is enriched with Lamp2, Cathepsin-D, and NPC1, while ZG fraction is enriched with Syncollin, Amylase, and trypsinogen. However, while isolated lysosomes (revised **Fig.S6B**, appear as clumps because we did not perform flush wash during isolation in order to keep lysosomes intact) are very positive (shown in pseudo-yellow color) with both AO (red) and LysoTracker (green), isolated ZGs are negative for both and Gal-9^-/-^ lysosomes indeed were less acidic. Therefore, it appears that mouse ZGs are different from rat ZGs (Niderau C, Gastroenterology,1986: 91: 1433), or in our hands, ZGs are not acidic so the AO signals in lysosome stability assay were mostly from lysosomes (revised **Fig.7G**). Correlated to this, there was reduced lysosomal hydrolase activity which suggest that Gal-9^-/-^ lysosomes are more unstable leading to increased permeability or reduced acidity. As suggested by the reviewer, activities of lysosomal hydrolases (revised **Fig.7D**) and levels of lysosomal membrane proteins (ATP6V1a in revised **Fig.7A**, NPC1 in revised **Fig.7D**) were shown. Western blotting (WB) of LC3 to measure autophagic flux (related references were cited) using fresh acinar cells stimulated with rapamycin/bafilomycin for 16hr was shown (revised **Fig.7E**). WB of amylase and CK19 from the same experiment was also shown to indicate isolated acinar cells were functionally intact during 16hr of culture and stimulation.

2. Different from their data in the intestine, the authors’ conclusion that Gal9 interacts with LAMP2 in acinar cells lacks sufficient evidence. IF (Figs 1A, S1A) shows Gal9 colocalization with ZG (amylase) and ER (GRP78) but, amazingly, there is no data indicating Gal9 presence in acinar cell lysosomes.

**Our response:**

As suggested by the reviewer, lysosomal localization of Gal-9 in fresh acinar cells by WB of cell fractionation was shown (revised **Fig.7A**). As we noticed that level of lysosomal Gal-9 in acinar cells, by WB fractionation analysis, is relatively low (we repeated 4 times), compared to ileum crypts (revised **Fig.3B**), we further performed colocalization of Gal-9 and Lamp2 in freshly-isolated acinar cells by immunofluorescence (revised Fig.**S6A**, yellow puncta). Both L and S-forms of Gal-9 exist in acinar cells.

3. A major weakness of the study is the use of general, not organ-specific, Gal9 KO. Gal9 is present in the immune/inflammatory cells and modifies their activity. Thus, it is impossible to make any mechanistic conclusion on the effect of Gal9 knockout on inflammation, a major pathologic response of pancreatitis – both local (pancreatic) and systemic.

1

**Our response:**

We completely agreed with reviewer’s comments. However, using freshly isolated acinar cells, we showed there were autophagy block (revised **Fig.7B**,**7C**), reduced lysosomal hydrolase activity (revised **Fig.7D**), impaired autophagic flux (revised **Fig.7E**), and increase ER stress (revised **Fig.7F**). As all these phenotypes were based on isolated cells, it means all acinar-cell phenotypes were cell-intrinsic defects. In addition, autophagy flux experiment was based on the stimulation of homogeneous acinar cells (revised **Fig.7E**), which indicates that impaired autophagy flux, intrinsically occurred in isolated acinar cells, could lead to more ER stress, higher LMP response, and increased apoptosis, which likely would cause pancreatitis.

In the case of Paneth cell-specific Gal-9 conditional knockout mice (Defa6-Cre^+^Gal-9^F/F^), while intra-epithelial and lamina propria compartments, both enriched with lymphocytes or other cell types, are in the immediate proximity of Paneth cells-localized crypts, Gal-9 derived or released from those lymphocytes or other cell types appeared not accessible or sufficient to rescue nearby Gal-9^-/-^ Paneth cells from autophagy block or cell degeneration (revised **Fig.2E-2K**). Therefore, although we do not have Pancreas-specific Gal-9 conditional knockout mice yet, it is reasonable to speculate that endogenous Gal-9 in acinar cells, which are autophagy-active cells similar to Paneth cells, could also be indispensable for preventing autophagy block or cell degeneration. That is, in the pancreas, we do not expect Gal-9 provided by nearby lymphocytes in the acini may significantly or functionally contributes to homeostasis of acinar cells. Furthermore, because we could not provide data related to Pancreas-specific Gal-9 conditional knockout mice, we tone down our conclusion with Gal-9-mediated pancreatitis by moving original Fig.1 to revised **Fig.7** to indicate this fact.

4. The authors’ conclusion that Gal9 KO mice develop spontaneous pancreatitis (p. 9) is based on only 2 parameters of pancreatitis, fibrosis and apoptosis. To make this conclusion solid, the authors should also measure hallmark responses of pancreatitis, such as changes in pancreatic levels of digestive enzymes, tryps inogen activation, acinar/ductal metaplasia.

**Our response:**

To address this concern, we have performed more pathological measurements. Based on Nat Comms 9:5033, 2018, we showed in Gal-9^-/-^ mice (revised **Fig.7I-J**,**S6F-G**), there were increased level of serum amylase, dilated ducts, enlarged islets of Langerhans, acinar vacuolization, and fatty replacement, which are associated with chronic pancreatitis evidenced in the above reference. For fibrosis, in addition to Sirius red staining (PNAS 112:E6166, 2015), we also showed increased α-SMA staining and mRNA level of α-SMA in Gal-9^-/-^ mice (J Immunol 198:3886, 2017). For acinar/ductal metaplasia, while we did not detect increased levels of amylase by WB of ZG fraction (revised Fig.**S6D**) or by IHC analysis (revised Fig.**S6F**), there were indeed increased amylase activity (revised Fig.**7D**), obvious dilated ducts, and slightly increased CK19 (revised **Fig.7I-J**,**S6F**) in Gal-9^-/­^mice, suggesting ductal metaplasia. While some typical parameters for pancreatitis might not be all observed in Gal-9^-/-^ mice, we would like to emphasize that cell-intrinsic autophagy block, impaired autophagy flux, more acinar ER stress, and higher LMP response could consequently cause acinar cell apoptosis (revised **Fig.7H**) and pancreatitis in aged KO mice due to lysosome dysfunction.

5. The authors conclude in the Abstract that Gal9 deficiency “impaired homeostatic cargo degradation,” which is not substantiated by specific measurements of autophagic flux in Gal9 null vs. WT acinar cells.

**Our response:**

We agreed with reviewer’s comments about this key data. Based on Autophagy 14:1060,2018, we have measured autophagy flux in isolated acinar cells using well-established protocol by treating fresh acinar cells with rapamycin and bafilomycin and performing WB of LC3 (revised **Fig.7E**). The result indicated an impaired cell-intrinsic autophagy flux in Gal-9^-/-^ acinar cells. WB of amylase and CK19 from the same experiment was also shown to indicate isolated acinar cells were functionally intact during 16hr of culture and stimulation.

6. Changes in IF intensity of LAMP2, LC3 and p62 (Fig. 1B) should be quantified and corroborated by measurements of LAMP2, LC3, and p62 levels with western blot.

**Our response:**

IF data in original Fig.1B were removed because of low quality. Instead, WB analyses of Lam p2, LC3, p62, GRP78, and CHOP, to indicate autophagy block and more ER stress in Gal-9^-/-^ mice, were shown (revised Fig.**7B**,**7F**).

2

7. Fig.2 discusses the effect of Gal9 deficiency on acute pancreatitis induced by cerulein (CERAP); saline-injected Gal9 KO mice serve as controls. Surprisingly, the data in saline-treated Gal9 KO vs WT mice show no signs of pancreatitis – contradicting the effects of Gal9 deficiency on pancreas shown in Fig.1 (on which the conclusion about spontaneous pancreatitis was based). For example, in Fig.1 Gal9 deficiency increased LC3 dots (Fig.1B), caused edema (Fig.1K) and acinar cell death (Fig.1J); but the data in Fig. 2G show no difference in edema, vacuolization, and cell death between saline-treated KO and WT pancreas (!). A possible explanation is that saline/CER injections were performed on much younger mice, but this should be examined. Anyway, the above discrepancies should be reconciled.

**Our response:**

We agreed with reviewer’s comments about data discrepancy. Indeed, cerulein-related data were earlier work using much younger (6~8 wks) mice as we initially focused on the model of “autophagy-mediated trypsinogen activation during cerulein injection”, in order to elucidate the role of Gal-9-mediated autophagy in pancreatitis. Our working model at that time was based on the study of J Cell Biol 181:1065, 2008, which appears to be controversial. As pointed out by Autophagy 6:428, 2010, the authors mentioned “it is not the autophagy induction per se but its impairment that is responsible for the pathological manifestations of pancreatitis”. So we felt we should go back to measure autophagy impairment and autophagy flux in Gal-9^-/-^ cells (as also suggested by the reviewer). Because of this concern and new autophagy flux data, we completely removed the original Fig.2 (i.e. all results related to cerulein injection/treatment) and focused on autophagy impairment (revised **Fig.7B**) and autophagy flux (revised **Fig.7E**), using freshly-isolated acinar cells from mice at the steady state (not cerulein or tunicamycin injection). Indeed, all clinical manifestations with pancreatitis and fibrosis were observed only in aged mice typically over 5-month-old.

I also have serious concerns RE the methods used:

1. Make sure to specify the genetic background of Gal9 KO mice.

**Our response:**

Gal-9^-/-^ mice we obtained were in B6 background as indicated in Am J Pathol 188:1225, 2018 (cited in the revised manuscript) and we have further backcrossed them to wild-type B6 mice for two more generations before intercrossing them to have Gal-9^+/+^ and Gal-9^-/-^ littermates for all experiments. To strengthen our conclusion with genetic evidence, we have generated Gal-9 conditional knockout (Gal-9^F/F^) mice in B6 background by the CRISPR/Cas9 technology. To minimize off-target effects in CRISPR mice, Gal-9 flox mice were first backcrossed to wild-type B6 mice for four generations before breeding into Paneth cell-specific Gal-9 conditional knockout mice (Defa6-Cre^+^Gal-9^F/F^) that we used in revised **Fig.2**. Unfortunately, Pancreas-specific Gal-9 conditional knockout (Ela-Cre^ERT2+^Gal-9^F/F^) mice are still in breeding and not ready for experiments, so we tone down our conclusion in Gal-9-mediated pancreatitis by moving original Fig.1 to revised **Fig.7** to indicate this fact.

2. The authors performed in vitro experiments using 24h or 48h acinar cell culture. Of note, isolated mouse or rat acinar cells undergo time dependent dedifferentiation, losing acinar and acquiring ductal cell phenotype (Sphyris N et al. Pancreas 2005; 30(2):14857); loss of endolysosomal markers, such as Rab5, EEA1 and LAMP1, have been described after 6h of culture (Messenger S et al. Am J Physiol Gastrointest Liver Physiol 2013;305:G439–G452). Thus, prolonged culture of acinar cells requires caution, various controls, and should be documented in great detail (e.g., Mareninova OA et al. J Clin Invest 2009;119:33403355).

To validate the in vitro/ex vivo system they use, the authors should assess changes in the levels of amylase, the ductal cell marker CK19, and the above endolysosomal markers; and functional parameters such as hormone-induced amylase secretion or trypsin activity. But the obvious question is: why not do the measurements on freshly isolated acinar cells, i.e., within the “standard” time frame of 6h incubation (of course, experiments on transduced cells require prolonged culture).

**Our response:**

We agreed with reviewer’s comments about prolonged acinar cell culture. To address the concern, we have now used freshly-isolated acinar cells for all experiments (revised **Fig.7A-D**,**7F**,**7G**,**Fig.S6**), except in revised **Fig.7E**, where acinar cells need to be cultured and stimulated with rapamycin/bafilomycin for 16hr in order to measure autophagy flux. We have tried shorter time for stimulation but 16hr appeared the best for autophagy induction. As a quality control, we also showed during 16hr of culture (revised **Fig.7E**), acinar cells maintain high expression of amylase and no CK19 was detected by WB (thus no acinar-to-ductal transition issue in this 16hr experiment).

3

3. The authors extensively use FACS analysis of acinar cells. This is, at best, problematic, as the standard pancreas digestion with collagenase yields a suspension of pancreatic acini, groups containing different numbers (from 3 to 20) of acinar cells. Moreover, the average size of isolated acini strongly depends on “hands” (and varies from one preparation to another even in the same hands). Thus, flow cytometry data on acinar cells are not reliable.

**Our response:**

To address the concern, we have minimized the use of flow cytometry for analysis but instead used Western blot, except in two experiments. First, for dye-based approach such as MDC and AO staining (revised **Fig.7C**), measurement by flow cytometry was needed. Second, as at the steady state, fresh acinar cells without exogenous insults have undetectable cleaved caspase-3 by WB, we therefore used Flow cytometry to measure caspase-3 activation in fresh acinar cells (revised **Fig.S6E**). Also, we would like to point out when analyzing by flow cytometry, acinar cells were actually gated by FSC/SSC such that only single acinar cell but not cell clumps, was analyzed for signals, which in this way gives more accurate readout about “the percentage” among whole cell population analyzed and thus minimizes the variation due to different batch of acinar cell preparation.

4. Many experiments in the paper include treatment of mice with tunicamycin, an inhibitor of endogenous N-glycosylation. LAMP2 is a highly N-glycosylated protein; thus, the authors should check LAMP2 glycosylation status in these experiments.

**Our response:**

We have performed Western blot of Lamp2 in several conditions (see **Fig.R1** below) to address the reviewer’s concern. Ex vitro culture (6 and 16hr) of acinar cells, treated with tunicamycin (Tm) or cerulein (CRE), did not alter Lamp2 glycosylation. Similarly, Lamp2 glycosylation was not altered in pancreas or fresh acinar cells isolated from mice at day-1 (Tm-d1) or day-2 (Tm-d2) post tunicamycin injection in vivo. As we have only one result with tunicamycin treatment in acinar cells (revised **Fig.7H**) and no results with cerulein treatment, the **Fig.R1** was not shown in the revised manuscript.

5. The authors measure trypsinogen activation with an enzymatic assay using Rhodamine 110, which can be cleaved by various proteases. Trypsin activity data should be remeasured using a selective trypsin substrate BocGlnAlaArgAMC, as done in numerous publications.

**Our response:**

In the revised manuscript, trypsin activity was re-measured using the specific substrate BocGlnAlaArgAMC (revised **Fig.7D**). Similarly, we have also used specific substrates when measuring lysosomal hydrolase activity (revised **Fig.7D**) as indicated in the section of “Materials & Methods”.

4

**Reviewer-2:** (Remarks to the Author)

This manuscript provides evidence that luminal galectin-9 promotes lysosome stabilization and that this is mediated via an interaction with Lamp2. This is an interesting idea, not least because it is conceptually opposite to the previously identified role of cytosolic galectins in lysosome homeostasis, in which they bind to exposed glycans when the limiting membrane of the lysosome is damaged, resulting in lysophagy. As the authors explain in their introduction, the specific role of galectins-3 and 8 in the molecular mechanism of lysophagy has previously been characterized. The starting point of the present manuscript was to address the question of whether Gal9 has a function in the homeostasis of autophagy active acinar cells in the pancreas. Using a mouse model (cerulein treatment) of acute pancreatitis, as well as acinar cells or tissue from Gal9^-/-^ mice, the authors provide compelling evidence that Gal9 is essential for maintaining lysosomal function and autophagy in pancreatic acinar cells. A key experiment shown in Fig.1E was to test lysosome stability by ‘photooxidation stimulation of acridine orange labelled acinar cells to induce lysosome membrane permeabilization (LMP)’. Gal9^-^/- cells showed increased susceptibility to LMP challenge. The authors also studied the effects of tunicamycin treatment, which inhibits N-glycosylation and has previously been shown to cause ER stress and LMP, finding that tunicamycin treated Gal9^-/-^ mouse cells showed increased autophagy blockade and greater ER stress. In addition to the characterization of acinar cells shown in Fig.1, the authors went on to provide data in Fig.2 that support their conclusion that cerulein-mediated induction of deleterious zymogens and subsequent necrotic pancreatitis requires Gal9-regulated autophagy. The authors then turned their attention to crypt cells in the gut, in which they showed (Fig.3) that Gal9 was predominantly expressed and found a similar requirement for Gal9 in maintaining lysosomal stability and affecting ERstress. Importantly, they were able to reproduce their findings in organoid cultures, a system in which they were able to do rescue experiments, showing that exogenous recombinant Gal9 could rescue the effects of Gal9 knockout. In Fig.4 the authors provide evidence that Gal9 regulates crypt homeostasis, antimicrobial immunity and inflammation. This is followed by a series of experiments (Figs 5 and 6) in CMT93 cells ± Gal9 CRISPR knockout, in which the core findings from the acinar cells and intestinal crypts were reproduced and which address the central question of how luminal Gal9 promotes lysosome stabilization. They obtained evidence that endogenous Gal9 cofractionates with LAMP positive organelles and showed in Gal9 knockout cells that they could rescue LMP and acidification defects with exogenous recombinant Gal9, not Gal3. They then reconstituted the Gal9 knockout cells with wild-type or mutant Gal9 to demonstrate the importance of the glycan-binding function of Gal9 for rescue. Pull down of FLAG-tagged Gal9 found LAMP1 and LAMP2 and the binding to LAMP2 was confirmed by coimmunoprecipitation. The authors finally propose a model in which luminal Gal9 strengthens or expands the glycocalyx, although no direct evidence is presented. Overall, this is a manuscript with an interesting central proposal and experiments that support it. However, it needs some additional experiments and a major rewrite before it can be published.

Major points

1. A major caveat to this review is that all information about the Gal9-/- mice in Materials and Methods was removed due to double-blind peer review process. Therefore, I have simply assumed that they are indeed Gal9-/- and have been appropriately genotyped and phenotyped.

**Our response:**

Gal-9^-/-^ mice we obtained were in B6 background as indicated in Am J Pathol 188:1225, 2018 (cited in the revised manuscript) and we have further backcrossed them to wild-type B6 mice for two more generations before intercrossing them to have Gal-9^+/+^ and Gal-9^-/-^ littermates for all experiments. To strengthen our conclusion with genetic evidence, we have generated Gal-9 conditional knockout (Gal-9^F/F^) mice in B6 background by the CRISPR/Cas9 technology. To minimize off-target effects in CRISPR mice, Gal-9 flox mice were first backcrossed to wild-type B6 mice for four generations before breeding into Paneth cell-specific Gal-9 conditional knockout mice (Defa6-Cre^+^Gal-9^F/F^) that we used in revised **Fig.2**. Unfortunately, Pancreas-specific Gal-9 conditional knockout (Ela-Cre^ERT2+^Gal-9^F/F^) mice are still in breeding and not ready for experiments, so we tone down our conclusion in Gal-9-mediated pancreatitis by moving original Fig.1 to revised **Fig.7** to indicate this fact.

2. It has previously been suggested that Gal9 senses damaged lysosomes, leading to lysophagy (see e.g. Papadopoulos C et al (2019) J Mol Biol. 2019 Aug 23. pii: S00222836(19)305194). In their introduction, the authors quote reference 13 with respect to Gal8 initiating anti-bacterial autophagy but should also point out that the same paper states that “Damage to lysosomes by glycyl-L-phenylalanine-2-naphthylamide (GPN) treatment resulted in the initial loss of lysotracker staining, followed by the appearance of galectin 3, 8 and 9 speckles“

**Our response:**

We thanked the reviewer to point out this. As suggested by the reviewer, we have rewritten the introduction with

reference cited as follows...

5

“Due to absence of complex glycans in the cytosol, cytosolic galectins were shown, likely only after vesicle rupture, to mediate a host surveillance mechanism for detection, repair, and clearance of damaged vesicles including lysosomes. Such lysosome-mediated lysophagy is believed to be aided by cytosolic galectins, including Gal-3, Gal-8, and Gal-9, which can sense sterile lysosomal damage and are rapidly recruited to damaged lysosomes via binding to exposed glycans from the lysosomal lumen.”

3. The authors use tunicamycin treatment as a model of LMP (they write “LMP challenge such as tunicamycin”). However, they have not themselves shown that it causes LMP in their cell types, nor have they shown that the ER stress that results is a consequence of LMP rather than being a direct effect at the level of the ER (as has been shown by many other groups in different cell types), which seems much more likely.

**Our response:**

We agreed with reviewer’s comments about the missing evidence to show the connection between tunicamycin, ER stress and LMP in CMT93 cells. To address this, we have performed a time-course (0-12hr) experiment by treating cells with tunicamycin (Tm) and then testing ER stress, LMP response, and apoptosis in the same stimulated cells accordingly. As shown in revised **Fig.3G-I**, the results showed Tm treatment in wild-type CMT93 cells induced ER stress as early as 1-3hr, detectable LMP after 6hr, and apoptosis around 9hr. Our results are consistent with J Pharmacol Exp Ther 329:524,2009 showing Tm with Sci Reports 7:1564, 2017 showing ER stress leads to a disruption in the lysosomal membrane (LMP), and with Nat Stru Mol Biol 25:217, 2018 showing Tm targets and inhibits GPT in the ER so should cause ER stress before LMP in lysosome. We have rewritten this part with references cited to support our conclusion in CMT93 cells.

4. Fig S3, Panels B,C, D (which needs quantification of colocalization) and G should be in main figures, not supplementary since they address the central proposition of the paper, which is that Gal9-LAMP2 interaction promotes lysosome stabilization.

**Our response:**

We agreed with reviewer’s comments and have made some changes. Original Fig.S3B was updated with WB of LC3/Lamp2 using cell fractionation and is now in revised **Fig.3B** (main figure). Original Fig.S3C was moved to revised **Fig.3F** (main figure), as suggested. Original Fig.S3D was updated with quantification and is now in revised **Fig.S3B**. The reason to still keep Fig.S3D in supplementary is because revised Fig.S3B is a LLOMe-treatment experiment that is conceptually linked to revised Fig.S3C-S3D when describing in the manuscript. Original Fig.S3G was updated with Gal-9-M6PR Co-IP and is now moved to revised **Fig.4E** (main figure) after mass spec result.

5. The data in Fig 4 showing that Gal9 regulates crypt homeostasis, anti-microbial immunity and inflammation is not central to the main message of the manuscript that luminal Gal9 promotes lysosome stabilization and would be better shown as supplementary information.

**Our response:**

We partially agreed with reviewer’s comments here. As in the title of original manuscript we did mention “luminal Gal-9-Lamp2 interaction ...prevents pathogenesis in the intestine...”, we think it is important to keep some of those data (revised **Fig.2C** for anti-microbial immunity, **Fig.2E** for crypt homeostasis) in the main figure to support the central message, which is that Gal-9 was reported as a risk factor for Crohn’s disease (CD) and the role of Gal-9 in CD is entirely unknown. The rationale for this is that the maintenance of crypt homeostasis and anti-microbial immunity is critical for intestinal pathogenesis. The remaining data (original Fig.4A to 4G) were all moved to supplementary result section (revised **Fig.S2B-S2J**), as suggested by the reviewer. Importantly, some previous data were obtained in global KO mice but now can be revised using Paneth cell-specific Gal-9 conditional knockout mice and will still be shown in main figures. For example, original Fig.4A (total crypt number) was replaced with revised **Fig.2E**, original Fig.4F (endoscopy) was replaced with revised **Fig.2I**.

6. The mass spec data for the pull down of FLAGtagged Gal9 must be shown. I do not understand why the authors only looked at the interaction of Gal9 with LAMP2. They state that LAMP2 is the most abundant LAMP , but they provide no evidence this is the case in the cells they are studying. It is certainly not true in HeLa cells, where mass spectrometry has shown ~4x more LAMP1 than LAMP2 (URL: <http://mapofthecell.org>).

**Our response:**

We thanked the reviewer to point out this and we have submitted the mass spec raw data as Excel file together with the analyses of heat-map and volcano-map in the revised manuscript. Regarding Lamp1/2 abundance in CMT93 cells, Gal-9 indeed pulled down both Lamp1 and Lamp2 (shown in Excel file) but when our output cut-

6

off threshold was set to score ≥ 300, Lamp1 became less significant and was not shown in the top 25 lysosomal Gal-9-binding targets while Lamp2 was the top 3 (revised **Fig.4D**), indicating in CMT93 cells Lamp2 is the major target for Gal-9. We have included the heat-map (revised **Fig.4D**) and volcano-map (revised **Fig.S4H**) data and also provided new data showing, in addition to Lamp2 (revised **Fig.5**), functional interaction of Gal-9 with M6PR (revised **Fig.4G-I**) also contributes to hydrolase activity and degradative autophagy in CMT93 cells. We also showed the M6PR protein structure with potential N-glycosylation sites (revised **Fig.S4I**). Unfortunately, as M6PR is huge (>300kDa) so it is technically difficult to perform mutagenesis study like Lamp2.

7. The authors propose a model in which luminal Gal9 strengthens or expands the glycocalyx, but wouldn’t a simpler model be that by binding to the glycocalyx it simply acts to block access of enzymes that hydrolyse glycans and thereby extends the halflife of the LAMPs ?

**Our response:**

We completely agreed with reviewer’s comments. Indeed we do not have any evidence to support our ”glycocalyx model” and there are indeed many possibilities about how Gal-9 might strengthen or expand the glycocalyx, as was also pointed out by the reviewer-3. In terms of this, we removed the model of Gal-9-regulated glycocalyx (original Fig.S4B), as we felt it is technically difficult for us to address this question and we think it is beyond the scope of this study. Instead, we chose to investigate the underlying mechanism about how Gal-9-Lamp2 interaction, with respect to poly-N-glycosylation, contributes to lysosome function, autophagy and cargo degradation. To this end, we spent significant amount of efforts to generate Lamp2 mutants and reconstituted cells (revised Fig.**S5D-F**), in order to identify critical N-glycosylation site(s) in Lamp2 that are bound by Gal-9 and regulates autophagy (revised **Fig.5D-H**). Correlated to this, the mechanism studies in Lamp2 were further extended (thanks to those comments from the reviewer-3) to examine whether the synthesis of poly-LacNAc chains (the key moieties bound by galectins including Gal-9) indeed contributes to autophagy and cargo degradation, and whether blocking the synthesis (by β3GnT2 and β4GalT1 gene knockdown in CMT93 cells) would mute the contribution of exogenous Gal-9 in the rescue experiment (revised **Fig.6**). While we could not address how Gal-9 affects glycocalyx as originally proposed (original Fig.S4B), we were able to reveal the contribution of functional interactions of Gal-9 with Lamp2 (and newly-identified Gal-9-M6PR via mass spec) to autophagy and cargo degradation, which leads to an updated model (revised **Fig.S5H**), based on our new data (Gal-9 is involved in M6PR-mediated lysosomal hydrolase activity in revised **Fig.4H** and cargo degradation in **Fig.4I**, the time-course study showing ‘’autophagy prevents ER stress->LMP->Caspase-3->apoptosis in revised **Fig.3G-I**).

Minor Points

1. P4 description of Fig 1A . What evidence is there of rapid induction of amylase+ acini after cerulein injection ? Only one time point is measured, at 8 h after the first injection and I h after the last.

**Our response:**

As we have completely removed cerulein-related data, we removed the statement “Gal-9 was rapidly induced in amylase^+^ acini after cerulein injection” accordingly. Now in the revised manuscript, we state that “we found at the steady state Gal-9 is weakly expressed in amylase-expressing acini (revised **Fig.S6A**)”.

2. P4. The PCR analysis described here measures mRNA – it cannot directly show that ’cerulein

induces rapid release of zymogens’ as stated.

**Our response:**

As we have completely removed cerulein-related data, we removed the statement accordingly.

3. P4. In contrast to the text statement, Fig 2G. shows that cerulein does greatly increase vacuolization and

necrosis in Gal9-/- mice, just not as much as in wild type mice. The current sentence implying no vacuolization

and necrosis in Gal9-/- mice is incorrect.

**Our response:**

As we have completely removed cerulein-related data, we removed the statement accordingly.

4. Fig S3B. The authors need to explain why they see no increase in LC3 in the Gal9-/- CMT93 cells compared

to WT cells.

**Our response:**

We have updated the data with WB of LC3 in CMT93 cells using lysosome fractionation (revised **Fig.3B**). It is

clear that Gal-9^-/-^ cells showed increased accumulation of LC3-II and p62.

5. Fig 5A. There needs to be a blot of the PNS loaded onto the step gradient, not just the gradient fractions. **Our response:**

7

In the revised manuscript, where applicable, we have included the PNS in all Western blot analysis with cell fractionation studies.

6. Fig6D. The EGF degradation experiment in Fig 6D must be quantified.

**Our response:**

We have updated the data with quantification (shown in revised **Fig.5C**).

7. In Figure legends it is stated that error bars indicate ‘means±SEM’ from ‘at least 2 independent experiments’. Instances where there are only 2 independent experiments should be identified and ‘means±range’ shown. Presumably none of the p values relate to situations where there are only 2 experiments.

**Our response:**

We thanked the reviewer to point out this.

(a) In all of our analyses, “n” indicates “the number of mice used in one experiment” or “the number of wells of cell culture used in one experiment”. For example, in organoid culture (revised **Fig.1I**, **2C**), “n” indicates one well of organoid culture which was also derived from one mouse. So “n” also indicates “the number of mice” here. In CMT93 cells, we always have multiple wells for untreated or treated conditions. “n” indicates cells, lysate, or supernatant harvested from several individual well of cell culture so it is not duplicated or triplicated samples from only one well of cell culture (as is pointed out in Fig.3 of J Cell Biol 177:7, 2007, which is ‘’inappropriate use of error bars”). Therefore, p values calculated from multiple independent wells of cell culture or from multiple mice indeed are statistically meaningful in a single experiment. We calculated p values based on the number of samples (or mice) in one experiment but not the number of experiments.
2. As described in J Cell Biol 177:7, 2007, standard deviation (SD) are used for descriptive error bars because they show how the data are spread. SD is calculated and represents the average or typical difference between the data points and their mean. About two thirds of the data points will lie within the region of mean ± 1 SD, and ~95% of the data points will be within 2 SD of the mean. The standard error of the mean (SEM) quantifies the precision of the mean. It is a measure of how far your sample mean is likely to be from the true population mean. Therefore, as we don’t know the “true population mean” in our samples, we think our datasets are better presented as “means±SD” instead of “means±range” or “means±SEM”.

8. P25. The section on reconstitution of Gal9 KO CMT93 cells is incomplete. There is no explanation of how the cells are reconstituted. Somewhere there also needs to be data showing, or a statement saying, that cells took up/expressed equal amounts of recombinant Gal9 in experiments where WT and mutants were being compared.

**Our response:**

We thanked the reviewer to point out this. We have updated the WB, by showing that Gal-9^-/-^ cells were reconstituted with Gal-9-WT or Gal-9-RA2 mutants and equally expressed (revised **Fig.S3A**,**S4C**). Similarly, we have also updated the WB, showing Lamp1/2^-/-^ cells reconstituted with Lamp2-WT or Lamp2 Asn-to-Asp mutants and their equal expression in HEK293T cells (revised **Fig.S5E**,**S5F**), as well as in CMT93 cells (revised **Fig.5D**).

9. The end of the Results section contains speculation about how Gal9 may be functioning that would more appropriately be placed in the Discussion.

**Our response:**

We thanked the reviewer to point out this. We have removed the statement and rewritten the result section, as

suggested.

8

**Reviewer-3:** (Remarks to the Author)

Galectin9 is a widely expressed galectin that is known to bind certain features of mammalian glycans, in particular poly-N-acetyl-lactosamine type sequences of repeating GalGlcNAc. These are glycan features known to occur on lysosome associated membrane proteins 1/2 (Lam p1/2). Here the authors identify a functional role of galectin-9 binding to Lamp2 in that regard which is interpreted to be important in lysosomal stabilization, ER stress/homeostasis, and other cellular pathways. The functions of galectins in general are widespread, as the proteins occur both in the cytoplasm, intracellularly within organelles (here within lysosomes), and on the cell surface and in secretions. Here the authors explore a key potential function of galectin-9, a secreted lectin that may acquire access to the lysosome via secretion and uptake into lysosomes via interactions with Lamp2. The study relates to the process of autophagy, or self degradation, whereby cellular material is degraded in survival responses, with an emphasis on pancreatic disorders and intestinal Paneth cells. The study is exciting in many ways and provides novel insights into both galectin-9 functions, lysosomal glycoproteins, and roles of autophagy in lysosomal function and pathogenesis. There are several questions the authors could address that could strengthen the impact of the study.

1. It is fundamentally not clear how galectin-9 binding to lam p1/2 and present in the lysosome in some way stabilizes the glycoproteins? What would that mechanisms be, and would it involve crosslinking, precipitation, or some other mechanism? Is the galectin-9 also stabilized there by its interactions with lamp1/2? This is an especially important issues, as the authors indicate, prior studies had concluded it was the glycosylation of the glycoproteins that was required for their stabilization, but here the authors indicate that interactions with galectin-9 are required.

**Our response:**

We thanked the reviewer to raise this key question. Based on new results, now we have more clues to revise (and update) our previous model, regarding whether binding of Gal-9 to Lam p1/2 stabilizes Lamp2 and how binding of Gal-9 to Lamp2 regulates Lamp2-mediated lysosome function.

(a) It appears that binding of Gal-9 to Lamp2 does not stabilize Gal-9 or Lamp2 protein per se. In Gal-9^-/-^ crypts or Gal-9^-/-^ CMT93 cells, Lamp2 expression was instead slightly increased (revised **Fig.1A**,**3B**) and this is likely due to intrinsic autophagy impairment in Gal-9^-/-^ cells leading to Lamp2 accumulation, or Gal-9 deficiency simply has no effect on Lamp2 protein stability per se. Similarly, “lysosomal expression level” of Gal-9 in Lam p1/2^KD^ or M6pr^KD^ cells was not dramatically decreased or altered (revised **Fig.4G**,**5A**), suggesting Lamp2 or M6PR deficiency has minimal effect on Gal-9 protein stability per se.

(b) We have data (**Fig.R2**, not shown in the revised manuscript) indicating that Lamp2 in Gal-9^-/-^ CMT93 cells was not more susceptible to Endo-H-mediated proteolysis (equally degraded by Endo-H, Endo-H dose and treatment time were experimentally determined), suggesting Gal-9 might not protect Lamp2 from proteolysis.

1. Both Gal-9 and Lamp1/2 deficiency leads to autophagy (flux) block (revised **Fig.3B**,**3E**,**5G**) that may associate with increased LMP response (revised **Fig.5B**) and impaired cargo degradation (revised **Fig.5C**). It was recently shown that Lamp2 overexpression reverses autophagy flux blockade which contributes to LMP and lysosomal cell death (Front Cell Dev Biol 8:31, 2020, cited in the Introduction section). Therefore, “N-glycan-mediated Gal-9-Lamp2 interaction-> maintain autophagy flux -> prevents LMP -> prevents cell death (revised **Fig.3G-I**)” is the main conclusion (summarized in revised **Fig.S5H** and please also see **Fig.R3** below) supported by our data and others (the above reference).
(a) Then the next question is how N-glycan-mediated Gal-9-Lamp2 interaction regulates autophagy flux (highlighted in red shown in **Fig.R3** below). We addressed this by analyzing several Lamp2 mutants, each

9

containing a single mutation of one N-glycosylation site (revised **Fig.5D**, Asn mutated to Asp). First, the result showed loss of a single N-glycosylation site in Lamp2 could cause decreased, increased, or unaltered Gal-9-Lamp2 interaction (by Co-IP), suggesting loss of one N-glycan chain (or one Gal-9-bound N-glycan chain) may affect the overall binding of Gal-9 to Lamp2. Second, among all Lamp2 mutants, loss of Asn^175^ in Lamp2 has a predominant effect leading to reduced Gal-9 binding (revised **Fig.5D**), autophagy block (revised **Fig.5E**,**5G**), reduced hydrolase activity (revised **Fig.5F**), increased ER stress and apoptosis (revised **Fig.5G**), and impaired cargo degradation (revised **Fig.5H**). All of these phenotypes were also found in Gal-9^-/-^ cells. As Gal-9-Lamp2 interaction does not stabilize Gal-9 or Lamp2 per se and loss of N-glycosylation sites does not de-stabilize Gal-9 or Lamp2 (revised **Fig.5D**,**S5F**), then the Asn^175^ mutant study clearly indicates that it is the Gal-9-bound N-glycan chain in Asn^175^ of Lamp2 that controls Lamp2-mediated lysosome function and its downstream functionality of autophagy. Furthermore, this regulation is very much mediated by the positioning of Gal-9-bound N-glycan chain in Asn^175^ of Lamp2 which is linked to Lamp2-mediated lysosome function. Therefore, at the steady state, N-glycan-mediated Gal-9-Lamp2 interaction via glycosylated moiety in Asn^175^ of Lamp2 is needed to maintain an unique conformation of glycosylated Lamp2, which facilitates functionality of lysosome and autophagy, but unlikely contributes to the physical strength of Lamp2-associated glycocalyx (our original model).

(e) Base on the [(](https://glyconnect.expasy.org/browser/proteins/2495)https://glyconnect.expasy.org/browser/proteins/2495), Asn^175^ of mouse Lamp2 was reported as a glycosite by mass spectrometry, which further supports our findings.
(f) Our response to 4. (see below) also pointed out that it is loss of Gal-9-bound N-glycan chain (as well as loss of one N-glycan chain) that could affect the functionality of Lamp2. This implies that the N-glycan chain, bound by Gal-9, indeed contributes to Lamp2 function.

(b) **Fig.R3** compares and summarizes our previous and revised models. Our revised model concludes that glycosylation of Lamp2 (and likely bound by Gal-9 via poly LacNAc chains in Asn^175^ of Lamp2) indeed is required for functionality of lysosome and cell homeostasis.

2. How does galectin-9 gain access to the lysosomal compartment in normal cells? Is it through endocytosis involving lamp1/2 or some other surface glycoproteins? The authors do not demonstrate mechanistically that lamp1/2 at the cell membrane are used in uptake of galectin-9, should comment on the possibility that membrane glycol-sphingolipids, which can also contain poly-N-acetyl-lactosamine moieties, might be important.

**Our response:**

We have performed several experiments in CMT93 cells to investigate the underlying mechanisms about

endocytosis of galectins into lysosomes.

10

(a) First, a time-course (0-4hr) chasing of the uptake of exogenous recombinant mouse Gal-9 (rmGal-9, which is S-form) into Gal-9^-/-^ cells (KO cells were used here to avoid the effect of endogenous Gal-9) indicated that the uptake of rmGal-9 into cells reaches its peak at 2hr (revised **Fig.S4A**). As a control, we blocked all surface glycan-binding of galectins by pre-treating cells with a-lactose for 2hr (Sci Reports 6:32024, 2016, Autophagy 11:1373,2015) before rmGal-9 was added. Indeed, there was no uptake after a-lactose pre-treatment. Notably, some basal level of rmGal-9 was detected which could be due to non-specific attachment of rmGal-9 to the cell membrane so they are not endocytosed or increased over time.

(b) We next performed WB with cell fractionation of rmGal-9-treated Gal-9^-/-^ cells at 2hr post uptake (based on the time-course experiment above), with or without prior a-lactose pre-treatment for 2hr, to trace intracellular localization of endocytosed rmGal-9 (revised **Fig.4A**). The result clearly indicated that almost all endocytosed rmGal-9 resides in lysosomes (undetectable in cytosol) at 2hr post uptake and that prior a-lactose pre-treatment indeed significantly reduced lysosomal localization (>50%) of rmGal-9. However, there was still some rmGal-9 detected in lysosome fractions even cells were pre-treated with a-lactose, supporting endocytosis of rmGal-9 into lysosomes can be a glycan-binding independent process.
(c) We next investigated lysosomal localization of Gal-9 when Gal-9 is endogenously-expressed (which is a more physiological condition) and whether glycan-binding of Gal-9 is involved in this condition (revised **Fig.4B**), using Gal-9^-/-^ cells stably reconstituted with Gal-9-WT (glycan-binding sufficient) or Gal-9-RA2 (glycan-binding deficient). The result showed there was no Gal-9 in lysosomes if Gal-9-RA2 was expressed, indicating that glycan-binding of Gal-9 is indeed needed for lysosomal localization of endogenously-expressed Gal-9.

(d) We next investigated whether Lamp1/2 is involved in lysosomal localization of Gal-9, using Lamp1/2^KD^ CMT93 cells (revised **Fig.5A**). The result showed there was slightly decreased lysosomal localization of Gal-9, suggesting Lamp1/2 (however, not sure it’s surface Lamp1/2 or lumenal Lamp1/2) does play a role in either uptake of Gal-9 via Lamp2 binding at the cell surface or has a role in the retention of Gal-9 in lysosomes via Lmap2 binding in the lysosomal lumen.

(e) We next investigated whether poly-LacNAc chains are involved in lysosomal localization of endocytosed rmGal-9, using p3GnT2^KD^Gal-9^-/-^ or p4GalT1^KD^Gal-9^-/-^ CMT93 cells (revised **Fig.6F**). Again, Gal-9^-/-^ cells were used to avoid the effect of endogenous Gal-9. The result showed that endocytosed rmGal-9 (S form) was significantly reduced in the absence of p3GnT2 or p4GalT1, indicating that poly-LacNAc chains play an important role in lysosomal localization of rmGal-9. Intriguingly, the detectable rmGal-9 in rmGal-9-treated p3GnT2^KD^Gal-9-/- and p4GalT1^KD^Gal-9^-/-^ cells suggests Gal-9 may bind to other N-glycans (i.e. the synthesis of those other N-glycans may not be affected by p3GnT2 or p4GalT1 deficiency), such as biantennary pyridylaminated oligosaccharide (BIPA) or a2-3-sialyl-lactose (SiaLac) (refer to revised **Fig.6A**), for its endocytosis and/or lysosomal localization. However, similar to **(d)**, it is not clear whether poly-LacNAc chains at the cell surface or in the lysosomal lumen are needed for the process of endocytosis and lysosomal localization of rmGal-9.

(f) In summary, lysosomal localization of Gal-9 involves endocytosis of Gal-9 in a glycan-binding dependent or independent manner. It also requires Lam p1/2 and poly-LacNAc chains but it is not clear the requirement occurs at the cell surface or in the lysosomal lumen. The above Western blot analyses were very pricey, particularly the cell fractionation studies where large amount of cells (which means a lot of pricey recombinant Gal-9 are needed as well) are needed. We could have done more analyses but due to cost and coronavirus pandemic, purchase and shipment of rmGal-9 from USA to Taiwan are significantly delayed, which prevents us from performing more experiments.

3. A key focal point of the paper is galectin-9 null mice, which is a general, not targeted, deletion of Galectin-9. This raises the question of the origin of galectin-9 in lysosomes, is it provided by lymphocytes, which also have a lot of the protein or is it provided by the acinar cells?

**Our response:**

We agreed with reviewer’s comments here. In the revised manuscript, we provide new evidence showing endogenous Gal-9 from Paneth cells plays an indispensable role in preventing autophagy block and intestinal pathogenesis (revised **Fig.2E-2K**), using Paneth cell-specific Gal-9 conditional knockout mice (Defa6-Cre^+^Gal-9^F/F^). Importantly, while intra-epithelial and lamina propria compartments, both enriched with lymphocytes or other cell types, are in the immediate proximity of Paneth cells-localized crypts, Gal-9 derived or released from those lymphocytes or other cell types appears not accessible or sufficient to rescue nearby Gal-9^-/-^ Paneth cells from autophagy block or cell degeneration (revised **Fig.2E-2K**). Therefore, although we do not have Pancreas-

11

specific Gal-9 conditional knockout mice yet, it is reasonable to speculate that endogenous Gal-9 in acinar cells, which are autophagy-active cells similar to Paneth cells, could also be indispensable for preventing autophagy block or cell degeneration. That is, in the pancreas, we do not expect Gal-9 provided by nearby lymphocytes in acini may significantly or functionally contributes to homeostasis of acinar cells. Furthermore, because we could not provide data related to Pancreas-specific Gal-9 conditional knockout mice, we tone down our conclusion with Gal-9-mediated pancreatitis by moving original Fig.1 to revised **Fig.7** to indicate this fact.

4. Galectin-9 is presumed to interact with the long chain poly-N-acetyl-lactosamine moieties as the authors indicate. If the synthesis of such chains is blocked, without affecting overall N-glycan biosynthesis, are the contributions of galectin-9 muted?

**Our response:**

The answer is Yes. Our new results showed poly-LacNAc chains are needed for full interaction of Gal-9 with Lamp2 (revised **Fig.6D**), for preventing accumulation of MDC^+^ autophagic vacuoles (revised **Fig.6E**), for preventing autophagy block, ER stress, and apoptosis (revised **Fig.6F**), and for executing cargo degradation (revised **Fig.6G**). Importantly, exogenous recombinant mouse Gal-9 (rmGal-9) did fail to rescue those defects in Gal-9^-/-^ cells when β3GnT2 or β4GalT1 was ablated (revised **Fig.6E-G**).

5. The authors refer to intracellular functions, but for this reviewer the authors should make a clear distinction between cytoplasmic localization, where galectin-9 and presumably all galectins are generated on cytoplasmic ribosomes, versus intra-organelle presence in the cytoplasm as in lysosomes.

**Our response:**

We thanked the reviewer to point out this. We have corrected our statements in the revised manuscript when describing “cytosolic galectins” vs “lysosomal galectins”, or cytosolic vs lysosomal localization of galectins.

6. A prior study by Itoh et al (2019), not referenced herein, showed that galectin-9 in prostate cancer cells can

induce apoptosis by entry into lysosomes and presumed association with ligands there.

**Our response:**

We have updated this information in the revised “Introduction” section, as follows.

“Similarly, endocytosed Gal-9 has been shown to accumulate in lysosomes and is involved in apical sorting of

proteins/lipids, as well as in the regulation of cancer cell death via autophagy or ubiquitination.” Reference-21

by Itoh et al (2019) is cited accordingly.

7. Galectin-9 is a tandem repeat galectin, and susceptible to cleavage by thrombin, depending on the isoforms. The authors should identify the isoforms (L or S) here in their studies in pancreatic acinar cells, and whether the galectin-9 within lysosomes is susceptible to proteolysis there.

**Our response:**

By WB with cell fractionation of fresh acinar cells (revised **Fig.7A**), we showed both L and S-forms of Gal-9 exist in the cytosol (abundant) and lysosome (very low). The presence of Gal-9 in lysosomes was further examined by immunofluorescence in steady-state (or fresh) acinar cells, co-stained with Gal-9 and Lamp2 (revised **Fig.S6A**). However, due to technical difficulty (low level of lysosomal Gal-9), we could not obtain sufficient amount of lysosomal Gal-9 from acinar cells to determine if it is susceptible to proteolysis.

8. In the tissues and cells examined in this study there are probably other galectin family members expressed, which may also bind poly-N-acetyl-lactosamine moieties and other N-glycans. The authors should provide information about the presence of other galectins if possible because of potential compensatory or conflicting activities of these other proteins.

**Our response:**

We thanked the reviewer to point out this. Indeed, Gal-1 and Gal-3 are very abundant in CMT93 cells, while expression level of Gal-8 is comparable to Gal-9 (revised **Fig.S3E**). However, all other endogenous galectins (Gal-1, Gal-3, Gal-8) did not exhibit compensatory function in Gal-9^-/-^ CMT93 cells. Similarly, fresh Gal-1^-/-^ and Gal-3^-/-^ crypts did not show lysosome instability (or higher LMP response), in contrast to Gal-9^-/-^ crypts (revised **Fig.1E**, **S2A**). Exogenous recombinant mouse Gal-3 (rmGal-3), in contrast to rmGal-9, failed to rescue formation of LC3^+^Lamp2^+^ autophagosomes during LLOMe-induced lysosome damage (revised **Fig.S3D**). Also, exogenous human recombinant Gal-1 (rhGal-1), rhGal-3, or rhGal-8, in contrast to rhGal-9, failed to reduce the accumulation of MDC^+^ autophagic vacuoles in human colon epithelial Gal-9^KD^ HT-29 cells (revised **Fig.S3F**). Overall, the presence of related galectin family members, such as Gal-1, Gal-3, and Gal-8, in either mouse CMT93 or human HT-29 cells, appears not functionally contributing to activities of Gal-9, suggesting there is no potential compensatory effects, derived from other galectins, on those phenotypes identified in Gal-9^-/-^ cells.

**Reviewer comments, second version:**

Reviewer #1 (Remarks to the Author):

The authors adequately responded to all my concerns


Reviewer #2 (Remarks to the Author):

In their revised manuscript the authors have strengthened their evidence that luminal galectin-9 promotes lysosome stabilization and that this is mediated via an interaction with Lamp2. I appreciate the care with which the authors have addressed my previous comments and concerns. The manuscript does contain some new data and considerable modifications of the original text. As a result, I have some further concerns but these may all be considered as minor points (although should be addressed):
1. The revised manuscript now contains data showing that, as summarised in the abstract, in gut epithelial cells Gal-9 is enriched in lysosomes and predominantly binds M6PR and LAMP2. The M6PR data are important. However, in many cell types M6PR has been shown to be present in pre-lysosomal/endosomal compartments and not lysosomes (see e.g. Griffiths, G. et al. 1988. Cell 52:329; Futter, C.E. et al. 1996. J. Cell Biol. 132: 1011). Indeed, lack of of M6PR has long been used as one of the criteria by which to define lysosomes see e.g. Mellman, I. 1996. Annu. Rev. Cell Dev. Biol. 12:575, who wrote ‘by convention, lysosomes are distinguished from LEs by virtue of the lysosome’s higher density on Percoll, the absence of MPR, and the operational feature of being the final site of accumulation of internalized macromolecules.’ I suggest that the authors use the term endolysosomes/lysosomes, to include the compartment created by fusion of late endosomes with lysosomes, when describing their enriched population of organelles containing both M6PR and LAMP2. Endolysosomes are defined in the excellent review by Huotari, J. & Helenius A. 2011. EMBO J 30: 3481 which provides a reasonably up-to-date description of the compartments in the endosomal/lysosomal system and the heterogeneity of the lysosomal compartment as a whole.
2. Page 8. I do not understand why the authors conclude that Gal-9 is involved in M6PR-mediated lysosomal transport. Their experiments do not address this directly. The data in Fig 4 H/I shows that as expected knockdown of M6PR causes reduction of lysosomal hydrolase activity and reduced degradation (not ‘autophagic degradation’ as stated in the Fig 4I legend) of endocytosed EGF in CMT93 cells but this does not imply any conclusion about Gal-9 function. Indeed, in 4H, the activity of Beta-Hex, an M6PR-trafficked enzyme is the same in the Gal9 -/- cells as in the WT cells.
3. Page 8. The description of M6PR traffic is partial and should be improved. The M6PR is thought to traffic in vesicles between the trans-Golgi network (where it binds M6P-tagged lysosomal enzymes) and endosomal compartments, with lysosomal enzymes being released from the receptor in the acidic lumen of endosomes, rather than itself entering ‘mature’ lysosomes. The empty receptors recycle to the he trans-Golgi network from endosomes.
4. In responding to one of my previous comments the authors have written “In all of our analyses, “n” indicates “the number of mice used in one experiment” or “the number of wells of cell culture used in one experiment”. For example, in organoid culture (revised Fig.1I, 2C), “n” indicates one well of organoid culture which was also derived from one mouse. So “n” also indicates “the number of mice” here. In CMT93 cells, we always have multiple wells for untreated or treated conditions.“n” indicates cells, lysate, or supernatant harvested from several individual well of cell culture so it is not duplicated or triplicated samples from only one well of cell culture (as is pointed out in Fig.3 of J Cell Biol 177:7, 2007, which is ‘’inappropriate use of error bars’’). Therefore, p values calculated from multiple independent wells of cell culture or from multiple mice indeed are statistically meaningful in a single experiment. We calculated p values based on the number of samples (or mice) in one experiment but not the number of experiments.”. I think that a modified version of this response, explaining the different definitions of n used in different experiments for statistical analysis should be added to the section on statistical analysis on p42 within Materials and Methods. That would make the basis of statistical analysis in the different experiments much clearer to readers.


Reviewer #3 (Remarks to the Author):

In this revision, the authors have addressed key criticism regarding their discovery that galectin-9 (Gal-9), a secreted lectin can enter lysosomes via secretion and uptake through binding to N-glycans on the lysosomal protein Lamp2. These interactions contribute to the process of autophagy, or self degradation, whereby cellular material is degraded in survival responses, with an emphasis on pancreatic disorders and intestinal Paneth cells. The study is exciting and interesting and provides novel insights into both galectin-9 functions, lysosomal glycoproteins, and roles of autophagy in lysosomal function and pathogenesis. The authors addressed most but not all issues regarding mechanisms in this study, and the revision is robust on many levels.

There is one point that the authors should address, and which remains unclear to this reviewer.

The presumed interaction of Gal-9 and M6PR is unclear, as the authors propose they directly interact through the N-glycans of the M6PR but the evidence for this is indirect.

The evidence for the localization of the M6PR in lysosomes is not presented at the ultrastructural level, and all evidence in the literature points to the absence of the M6PR in the lysosome, but it is present in acidified endosomes and perhaps so-called ‘prelysomes’.

Indeed, the authors show that knockdown of the M6PR does not affect lysosomal localization of Gal-9, but as expected results in reduced lysosomal enzyme targeting.

It seems a misinterpretation of the data to then conclude that “These results indicate Gal-9 is involved in M6PR-mediated lysosomal transport and consequently affects functionality of lysosome and autophagy”.

The evidence for the localization of the M6PR in lysosomes is not presented at the ultrastructural level.

By contrast, the authors show more convincingly that Lamp1/2 interacts with Gal-9 in lysosomes and that this interaction is important, as the knockdown of Lamp1/2 (Lamp1/2KD) caused reduced Gal-9 lysosomal localization (Fig.5A).

Thus, while the authors have demonstrated that there is an interaction of Gal-9 with poly-N-acetyl-lactosamine and this is involved in regulating autophagy and cargo degradation, the direct involvement of the M6PR in this is ambiguous and the authors should further clarify.

**Author rebuttal, second version:**

**Reviewer-2:** (Remarks to the Author)

In their revised manuscript the authors have strengthened their evidence that luminal galectin-9 promotes lysosome stabilization and that this is mediated via an interaction with Lamp2. I appreciate the care with which the authors have addressed my previous comments and concerns. The manuscript does contain some new data and considerable modifications of the original text. As a result, I have some further concerns but these may all be considered as minor points (although should be addressed).

(1) The revised manuscript now contains data showing that, as summarised in the abstract, in gut epithelial cells Gal-9 is enriched in lysosomes and predominantly binds M6PR and LAMP2. The M6PR data are important. However, in many cell types M6PR has been shown to be present in pre-lysosomal/endosomal compartments and not lysosomes (see e.g. Griffiths, G. et al. 1988. Cell 52:329; Futter, C.E. et al. 1996. J. Cell Biol. 132: 1011). Indeed, lack of of M6PR has long been used as one of the criteria by which to define lysosomes see e.g. Mellman, I. 1996. Annu. Rev. Cell Dev. Biol. 12:575, who wrote ‘by convention, lysosomes are distinguished from LEs by virtue of the lysosome’s higher density on Percoll, the absence of MPR, and the operational feature of being the final site of accumulation of internalized macromolecules.’ I suggest that the authors use the term endolysosomes/lysosomes, to include the compartment created by fusion of late endosomes with lysosomes, when describing their enriched population of organelles containing both M6PR and LAMP2. Endolysosomes are defined in the excellent review by Huotari, J. & Helenius A. 2011. EMBO J 30: 3481 which provides a reasonably up-to-date description of the compartments in the endosomal/lysosomal system and the heterogeneity of the lysosomal compartment as a whole.

**Our response:**

We thank the reviewer’s correction and appreciate his/her professional comments here. To determine whether M6PR is more localized in pre-lysosomal/endosomal compartments in CMT93 cells, we have performed three high-resolution fractionation experiments.

(a) In the 1^st^ experiment, a regular protocol (PLoS One 9:e91465, 2014) for subcellular fractionation of WT vs M6pr^KD^ CMT93 cells was tested (**Fig.R1**, shown below but not in the revised manuscript). The result showed that with this protocol, the so-called lysosome fraction (Lyso) basically contains non-cytosol (by α-Tubulin marker) and some LM fractions (by Syntaxin 6 marker), but this protocol could not distinguish Lamp2^+^M6PR^-^ lysosomes from Lamp2^-^M6PR^+^ early endosomes or Lamp2^+^M6PR^+^ late endosomes (the definition is based on Fig.1 in Annu Rev Cell Dev Biol 12:575, provided by the reviewer-2). As shown below, M6PR is enriched in Lyso fraction which in this case also contains early/late endosomes, as well as in LM fraction which is marked by Syntaxin 6, a marker for the trans-Golgi network and endosomal compartments.

|  | **Fig.R1. The crude subcellular fractionation of CMT93 cells.**  PNS: post nuclear supernatant, Cyto: cytosol fraction, LM: light membrane fraction, Lyso: lysosome fraction. Lamp2 is a marker for late endosome and lysosome. α-Tubulin is a marker for cytosol. Syntaxin 6 is a marker for the trans-Golgi network and endosomal compartments. This fractionation protocol was from PLoS One 9:e91465, 2014. |
| --- | --- |

(b) In the 2^nd^ experiment, we searched and found the protocol which could distinguish Cathepsin-D^+^ lysosomes from EEA1^+^ early endosomes (Fig.3b in Nature Communications 6:8876, 2015). To better distinguish lysosomes from early and late endosomes, we slightly modified the density step-gradient (from the original 2.5%-to-30% to

1

7.5%-to-42%) and optimized the condition (the result of the 2^nd^ experiment was not shown here or in the revised manuscript).

**(c)** In the 3^rd^ experiment using our optimized protocol, the result below showed Lamp2^+^M6PR^-^ lysosomes are in the fraction 1-to-2, Lamp2^+^M6PR^+^ late endosomes are enriched in the fraction 3-to-7, and Lamp2^-^M6PR^+^ early endosomes are enriched in the fraction 9-to-15 (**Fig.R2**). EEA1 ad Rab5 are markers for early endosomes, while Syntaxin 6 is a marker for the trans-Golgi network and endosomal compartments. Calreticulin is a marker for ER. Therefore, based on this result (also shown in **Fig.S5C** in the revised manuscript) and several references provided by the reviewer-2, we have updated our description about the M6PR (on Page-8) and used the term endolysosomes/lysosomes, where applicable (all modified texts in the revised manuscript were labeled in red color) to include the compartments created by fusion of late endosomes with lysosomes, when describing enriched population of such organelles. Also suggested by the reviewer, we have corrected our conclusion to that ‘’Gal-9 is likely not involved in M6PR-mediated endolysosomal transport and cargo degradation”, because 1) lack of the direct evidence showing a role for Gal-9 in those defects identified in M6pr^KD^ cells (revised Fig.4F,G), and 2) lysosomal localization of Gal-9 was not altered in M6pr^KD^ cells (revised Fig.4H).

**Fig.R2. The subcellular fractionation of CMT93 cells by density step-gradient ultra-centrifugation.** PNS: post nuclear supernatant, Lyso: lysosome fraction, LE: late endosome fraction, EE: early endosome fraction. EEA1 and Rab5 are markers for early endosomes. Syntaxin 6 is a marker for the trans-Golgi and endosomal compartments. Calreticulin is a marker for ER. This protocol was slightly modified from Nature Communications 6:8876, 2015.

(2) Page 8. I do not understand why the authors conclude that Gal-9 is involved in M6PR-mediated lysosomal transport. Their experiments do not address this directly. The data in Fig 4 H/I shows that as expected knockdown of M6PR causes reduction of lysosomal hydrolase activity and reduced degradation (not ‘autophagic degradation’ as stated in the Fig 4I legend) of endocytosed EGF in CMT93 cells but this does not imply any conclusion about Gal-9 function. Indeed, in 4H, the activity of Beta-Hex, an M6PR-trafficked enzyme is the same in the Gal9 -/- cells as in the WT cells.

**Our response:**

We thank the reviewer’s comments here. We have corrected our conclusion to that ‘’Gal-9 is likely not involved in M6PR-mediated endolysosomal transport and cargo degradation” on Page-8. Where applicable, we have used the term ‘’cargo degradation or degradation” instead of ‘’autophagic degradation”, in the Fig 4I (now revised Fig.4G) legend and others. All modified texts were labeled in red color.

(3) Page 8. The description of M6PR traffic is partial and should be improved. The M6PR is thought to traffic in vesicles between the trans-Golgi network (where it binds M6P-tagged lysosomal enzymes) and endosomal

2

compartments, with lysosomal enzymes being released from the receptor in the acidic lumen of endosomes, rather than itself entering ‘mature’ lysosomes. The empty receptors recycle to the trans-Golgi network from endosomes.

**Our response:**

We thanked reviewer’s comments here. We have updated the description about M6PR traffic/recycling on Page-

8, with the reference for readers and supported by the new **Fig.S5C**. All modified texts were labeled in red color.

(4) In responding to one of my previous comments the authors have written “In all of our analyses, “n” indicates “the number of mice used in one experiment” or “the number of wells of cell culture used in one experiment”. For example, in organoid culture (revised Fig.1I, 2C), “n” indicates one well of organoid culture which was also derived from one mouse. So “n” also indicates “the number of mice” here. In CMT93 cells, we always have multiple wells for untreated or treated conditions.“n” indicates cells, lysate, or supernatant harvested from several individual well of cell culture so it is not duplicated or triplicated samples from only one well of cell culture (as is pointed out in Fig.3 of J Cell Biol 177:7, 2007, which is ‘’inappropriate use of error bars”). Therefore, p values calculated from multiple independent wells of cell culture or from multiple mice indeed are statistically meaningful in a single experiment. We calculated p values based on the number of samples (or mice) in one experiment but not the number of experiments.”. I think that a modified version of this response, explaining the different definitions of n used in different experiments for statistical analysis should be added to the section on statistical analysis on p42 within Materials and Methods. That would make the basis of statistical analysis in the different experiments much clearer to readers.

**Our response:**

We thank the reviewer’s comments here. We have provided a detailed description about how ‘n’ was represented in each figure on Page 40-42, under the section of ‘Statistical analysis’ within Materials and Methods. All modified or added texts were labeled in red color.

3

**Reviewer-3:** (Remarks to the Author)

In this revision, the authors have addressed key criticism regarding their discovery that galectin-9 (Gal-9), a secreted lectin can enter lysosomes via secretion and uptake through binding to N-glycans on the lysosomal protein Lamp2. These interactions contribute to the process of autophagy, or self degradation, whereby cellular material is degraded in survival responses, with an emphasis on pancreatic disorders and intestinal Paneth cells. The study is exciting and interesting and provides novel insights into both galectin-9 functions, lysosomal glycoproteins, and roles of autophagy in lysosomal function and pathogenesis. The authors addressed most but not all issues regarding mechanisms in this study, and the revision is robust on many levels.

There is one point that the authors should address, and which remains unclear to this reviewer.

The presumed interaction of Gal-9 and M6PR is unclear, as the authors propose they directly interact through

the N-glycans of the M6PR but the evidence for this is indirect.

**Our response:**

While we did show in the original **Fig.4E** that Gal-9 co-immunoprecipitates with M6PR in a N-glycan-dependent manner, using Gal-9^-/-^ CMT93 cells stably reconstituted with N-glycan-binding sufficient wild-type Gal-9 or N-glycan-binding deficient mutant (RA2) Gal-9, knockdown of M6PR did not alter lysosomal localization of Gal-9 (original **Fig.4G**). As suggested by the reviewer, the Gal-9-M6PR interaction could be indirect. Therefore, we have modified our conclusion to ‘’Gal-9 might indirectly associates with M6PR and is likely not involved in M6PR-mediated endolysosomal transport and cargo degradation” on Page-8.

The evidence for the localization of the M6PR in lysosomes is not presented at the ultrastructural level, and all evidence in the literature points to the absence of the M6PR in the lysosome, but it is present in acidified endosomes and perhaps so-called ‘prelysomes’.

Indeed, the authors show that knockdown of the M6PR does not affect lysosomal localization of Gal-9, but as expected results in reduced lysosomal enzyme targeting.

It seems a misinterpretation of the data to then conclude that “These results indicate Gal-9 is involved in M6PR-mediated lysosomal transport and consequently affects functionality of lysosome and autophagy”.

The evidence for the localization of the M6PR in lysosomes is not presented at the ultrastructural level.

By contrast, the authors show more convincingly that Lamp1/2 interacts with Gal-9 in lysosomes and that this interaction is important, as the knockdown of Lamp1/2 (Lamp1/2KD) caused reduced Gal-9 lysosomal localization (Fig.5A).

Thus, while the authors have demonstrated that there is an interaction of Gal-9 with poly-N-acetyl-lactosamine and this is involved in regulating autophagy and cargo degradation, the direct involvement of the M6PR in this is ambiguous and the authors should further clarify.

**Our response:**

We thank the reviewer’s correction and appreciate his/her professional comments here. To determine whether M6PR is more localized in pre-lysosomal/endosomal compartments in CMT93 cells, we have performed three high-resolution fractionation experiments.

**(a)** In the 1^st^ experiment, a regular protocol (PLoS One 9:e91465, 2014) for subcellular fractionation of WT vs M6pr^KD^ CMT93 cells was tested (**Fig.R1**, shown below but not in the revised manuscript). The result showed that with this protocol, the so-called lysosome fraction (Lyso) basically contains non-cytosol (by α-Tubulin marker) and some LM fractions (by Syntaxin 6 marker), but this protocol could not distinguish Lamp2^+^M6PR^-^ lysosomes from Lamp2^-^M6PR^+^ early endosomes or Lamp2^+^M6PR^+^ late endosomes (the definition is based on Fig.1 in Annu Rev Cell Dev Biol 12:575, provided by the reviewer-2). As shown below, M6PR is enriched in Lyso fraction which in this case also contains early/late endosomes, as well as in LM fraction which is marked by Syntaxin 6, a marker for the trans-Golgi network and endosomal compartments.

4

|  | **Fig.R1. The crude subcellular fractionation of CMT93 cells.**  PNS: post nuclear supernatant, Cyto: cytosol fraction, LM: light membrane fraction, Lyso: lysosome fraction. Lamp2 is a marker for late endosome and lysosome. α-Tubulin is a marker for cytosol. Syntaxin 6 is a marker for the trans-Golgi network and endosomal compartments. This fractionation protocol was from PLoS One 9:e91465, 2014. |
| --- | --- |

(b) In the 2^nd^ experiment, we searched and found the protocol which could distinguish Cathepsin-D^+^ lysosomes from EEA1^+^ early endosomes (Fig.3b in Nature Communications 6:8876, 2015). To better distinguish lysosomes from early and late endosomes, we slightly modified the density step-gradient (from the original 2.5%-to-30% to 7.5%-to-42%) and optimized the condition (the result of the 2^nd^ experiment was not shown here or in the revised manuscript).
(c) In the 3^rd^ experiment using our optimized protocol, the result below showed Lamp2^+^M6PR^-^ lysosomes are in the fraction 1-to-2, Lamp2^+^M6PR^+^ late endosomes are enriched in the fraction 3-to-7, and Lamp2^-^M6PR^+^ early endosomes are enriched in the fraction 9-to-15 (**Fig.R2**). EEA1 ad Rab5 are markers for early endosomes, while Syntaxin 6 is a marker for the trans-Golgi network and endosomal compartments. Calreticulin is a marker for ER. Therefore, based on this result (also shown in **Fig.S5C** in the revised manuscript) and several references provided by the reviewer-2, we have updated our description about the M6PR (on Page-8) and used the term endolysosomes/lysosomes, where applicable (all modified texts in the revised manuscript were labeled in red color) to include the compartments created by fusion of late endosomes with lysosomes, when describing enriched population of such organelles. Also suggested by the reviewer, we have corrected our conclusion to that ‘’Gal-9 is likely not involved in M6PR-mediated endolysosomal transport and cargo degradation”, because 1) lack of the direct evidence showing a role for Gal-9 in those defects identified in M6pr^KD^ cells (revised Fig.4F,G), and 2) lysosomal localization of Gal-9 was not altered in M6pr^KD^ cells (revised Fig.4H).

**Fig.R2. The subcellular fractionation of CMT93 cells by density step-gradient ultra-centrifugation.** PNS: post nuclear supernatant, Lyso: lysosome fraction, LE: late endosome fraction, EE: early endosome fraction. EEA1 and Rab5 are markers for early endosomes. Syntaxin 6 is a marker for the trans-Golgi and endosomal compartments. Calreticulin is a marker for ER. This protocol was slightly modified from Nature Communications 6:8876, 2015.

**Reviewer comments, third version:**

Reviewer #2 (Remarks to the Author):

The authors have provided detailed and satisfactory responses to my comments.


Reviewer #3 (Remarks to the Author):

The revised manuscript here demonstrates more fully and more convincingly that galectin-9 in lysosomes taken up by endocytic pathways in a carbohydrate-dependent mechanism that leads to interactions with a major lysosomal glycoprotein Lamp-2 and that interaction leads to enhancement of lysosome function and facilitated autophagy.

A key question in the prior review was the source of exogenous galectin-9 in the intestine and pancreas, and new data shown here indicate that Paneth cells are an important source, using specifically Paneth-targeted galectin-9 deletion in mice (Defa6-Cre+Gal-9F/F). While the source in pancreas was not studied, the authors predict, with some reasonableness, that pancreatic acinar cells may be that source. Furthermore, the authors addressed a key question about the potential nature of the glycans interacting with galectin-9, and used when beta3GnT2 or beta4GalT1 ablation to explore that, which abrogated interactions. And finally, a key question was the specificity of the observations with regard to galectin-9, as this is just one member of a large family of related proteins. The new data here indicate that the effects are specific to galectin-9 and not replicated by other galectins tried, in particular Gal-1, Gal-3, and Gal-8.

Overall, this revised manuscript has addressed the key concerns and presents a robust and convincing set of evidence demonstrating a key role for galectin-9 in lysosomal and autophagy functions.

**Author rebuttal, third version:**

REVIEWERS' COMMENTS:

**Reviewer #2** (Remarks to the Author)

The authors have provided detailed and satisfactory responses to my comments.

**Our response:**

We thank that the reviewer was satisfied with our response to all concerns raised previously.

**Reviewer #3** (Remarks to the Author)

The revised manuscript here demonstrates more fully and more convincingly that galectin-9 in lysosomes taken up by endocytic pathways in a carbohydrate-dependent mechanism that leads to interactions with a major lysosomal glycoprotein Lamp-2 and that interaction leads to enhancement of lysosome function and facilitated autophagy.

A key question in the prior review was the source of exogenous galectin-9 in the intestine and pancreas, and new data shown here indicate that Paneth cells are an important source, using specifically Paneth-targeted galectin-9 deletion in mice (Defa6-Cre+Gal-9F/F). While the source in pancreas was not studied, the authors predict, with some reasonableness, that pancreatic acinar cells may be that source. Furthermore, the authors addressed a key question about the potential nature of the glycans interacting with galectin-9, and used when beta3GnT2 or beta4GalT1 ablation to explore that, which abrogated interactions. And finally, a key question was the specificity of the observations with regard to galectin-9, as this is just one member of a large family of related proteins. The new data here indicate that the effects are specific to galectin-9 and not replicated by other galectins tried, in particular Gal-1, Gal-3, and Gal-8.

Overall, this revised manuscript has addressed the key concerns and presents a robust and convincing set of evidence demonstrating a key role for galectin-9 in lysosomal and autophagy functions.

**Our response:**

We thank the reviewer has raised key questions in the prior review for us to strengthen our manuscript and we

thank the reviewer was satisfied with our response to all concerns raised previously.
